# Probe Signal Values in mRNA Arrays Imply an Excessive Involvement of Neutrophil FCGR1 in Tuberculosis

**DOI:** 10.3389/fmed.2020.00019

**Published:** 2020-02-14

**Authors:** Kang Wu, Meng Li, Zhen-yan Chen, Douglas B. Lowrie, Xiao-Yong Fan

**Affiliations:** ^1^Key Laboratory of Medical Molecular Virology of MOE/MOH, Shanghai Public Health Clinical Center, Fudan University, Shanghai, China; ^2^School of Laboratory Medicine and Life Science, Wenzhou Medical University, Wenzhou, China; ^3^TB Center, Shanghai Emerging and Re-emerging Institute, Shanghai, China; ^4^Department of Life Science, Bengbu Medical College, Bengbu, China

**Keywords:** *Mycobacterium tuberculosis*, whole blood, neutrophils, gene signature, probe signal value, Fc fragment of IgG receptor I

## Abstract

The perturbed genes from transcriptomes are often presented in terms of relative expressions against control samples. However, the probe signal values (PSVs) of genes, implying protein abundances, are often ignored. Here, we explored the PSVs in tuberculosis (TB)-relevant signature genes. The signatures from *Mycobacterium tuberculosis*-infected THP-1 cells were defined as induced (T*Mtb*-i, with a derived T*Mtb*-iNet) and repressed (T*Mtb*-r). The signature from human blood was defined as a pulmonary TB (PTB)-specific signature (PTBsig). The analysis showed that before infection, T*Mtb*-i and T*Mtb*-iNet had lower PSVs and T*Mtb*-r genes had average PSVs. In the blood of healthy donors, PTBsig (divided into up-regulated PTBsigUp and down-regulated PTBsigDn) displayed average PSVs. This was partly due to masking by the cellular heterogeneity of blood; diverse PSVs were seen in constituent cell populations (CD4/8+ T, monocytes and neutrophils). Specifically, the PSVs of PTBsigUp in the neutrophils of healthy donors were higher (implying higher protein abundances), and much higher in the neutrophils of PTB (implying excessive protein abundances). Based on the PSV patterns of PTBsigUp in four cell populations, we identified three representative highly homologous genes (*FCGR1A, FCGR1B*, and the pseudogene *FCGR1CP*, which were often poorly distinguished), of which the summed PSVs were the highest in the neutrophils of PTB patients and healthy donors. The three genes were all up-regulated and responsive to chemotherapy in the blood of PTB, as validated in an RNA-seq-based analysis. This PSV-based study confirms the excessive involvement of neutrophil FCGR1 in PTB.

## Introduction

Transcriptome profiling of tuberculosis (TB)-relevant samples, whether from humans (1–3), model animals (4, 5), or immortalized cell lines (6), have been widely utilized to deepen our understanding of TB in many respects, e.g., pathogenesis, diagnosis, and prognosis. The biological samples used for transcriptome profiling have a wide range of purity, ranging from *in vitro* homogeneous cell lines to highly heterogeneous *in vivo*/*ex vivo* samples. Clearly, the readout from the latter is the sum from the various included kinds of cells, which contribute differently.

Irrespective of whether a hybridization-based platform or a sequencing-based platform (7, 8) has been used, the typical premier readout/illustration of transcriptome data has been the relative gene expression between groups of relevant samples and pre-defined groups of control samples (9, 10). However, in contrast to the relative expression, the actual probe signal values (PSVs) of perturbed signature genes have generally been ignored, even though the PSVs of the perturbed signature genes have been reliably detected (technically unreliable/undetected ones have been filtered out prior to bioinformatics analysis). In this study, the term “probes” mean the fluorescently labeled nucleic acids/genes comprising an RNA sample that would hybridize to the corresponding gene-specific DNA fragment of a gene array in a dose-dependent manner. The term PSV means the normalized and then log_2_-transformed probe signal strength of any gene in a gene array/sample. We hypothesized that analysis of the PSVs underlying published gene signatures might reveal additional biologically relevant information, since PSVs are taken to broadly reflect the abundance of mRNA, which in turn is a critical determinant of cognate protein abundance. Accordingly, we explored and compared the PSVs of signature genes identified from a homogeneous cell line as a model and then from heterogeneous *in vivo* whole blood. The signatures from the *Mtb*-infected THP-1 cell line were previously identified by us as the induced signature (T*Mtb*-i, from genes that were induced/up-regulated after *Mtb* infection) and its derived network-based signature (T*Mtb*-iNet) and a repressed gene signature (T*Mtb*-r) from genes that were repressed/down-regulated after *Mtb* infection (6, 11). The signature from whole blood was the pulmonary TB (PTB)-specific signature PTBsig identified, by Berry et al. (10), through comparing the transcriptome data of whole blood in PTB patients to those in latent TB-infected (LTBI) donors and healthy control (HC) donors. It was divided into the up-regulated and down-regulated sub-signatures, termed PTBsigUp and PTBsigDn, respectively (10, 12).

The cell line-derived signatures T*Mtb*-i and T*Mtb*-iNet are interferon-related signatures and T*Mtb*-r is a functionally undefined signature (6, 12). The whole blood signature PTBsig is similarly an interferon-inducible blood signature and is present in neutrophils rather than in CD4+ T cells, CD8+ T cells, or monocytes (10). In essence, the numerical predominance and larger gene expression in neutrophils inevitably account for the PTBsig in whole blood (12). However, neutrophils appear to mainly contribute to pathology rather than protection against the bacteria in TB. Furthermore, the determinants of the underlying balance of innate and acquired immunity are not currently resolved and are likely to be complex (13–15). This investigation of PSVs indicates that a high degree of expression of Fc receptor for IgG (i.e., Fc fragments of IgG receptor; FCGR1A, FCGR1B) on neutrophils may be a key signature of pulmonary TB.

## Results

### T*Mtb*-i and T*Mtb*-iNet Genes Are at Lower PSVs (Implying Lower/Negligible Protein Abundances) in Homogeneous THP-1 Cells Prior to *Mtb* Infection, Whereas T*Mtb*-r Genes Are at Average PSVs

**Figure 1:**
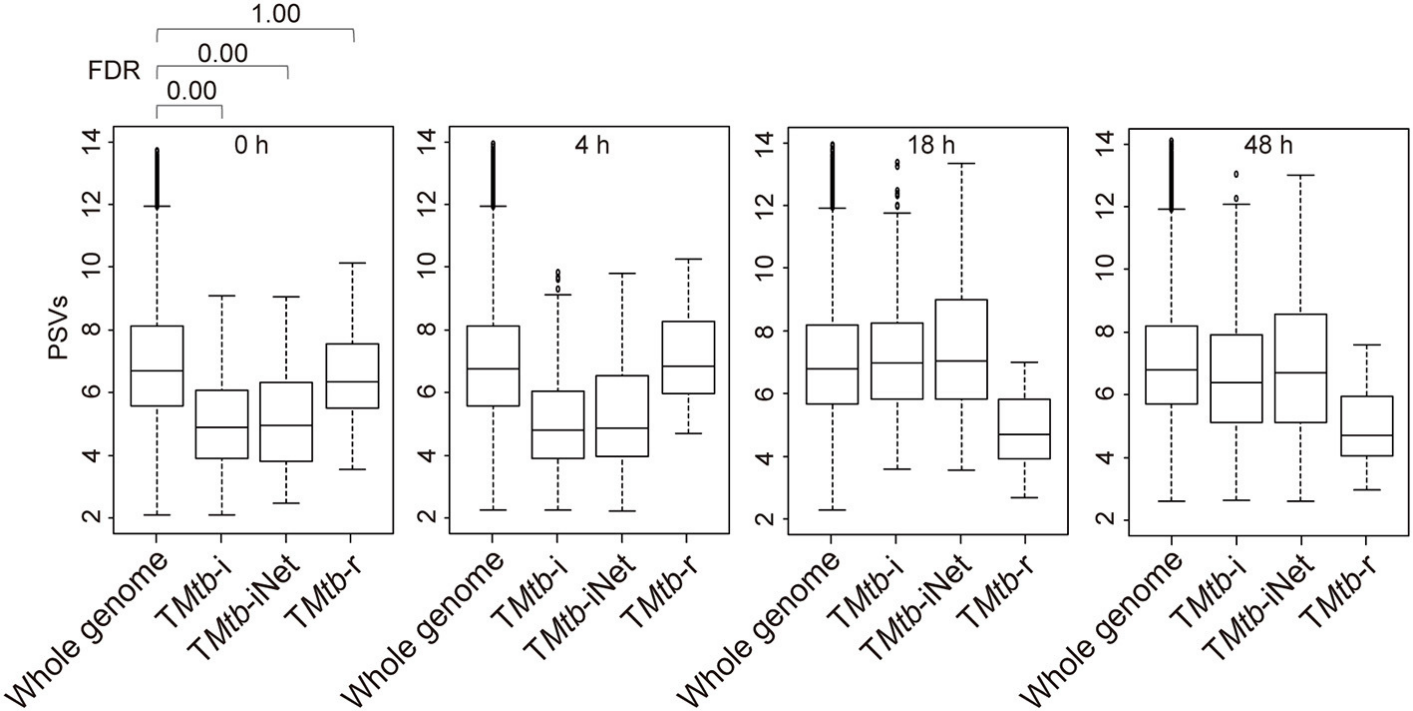
Box plots of the PSVs from THP-1 cells responding to *Mtb* infection. PSVs refer to the normalized and log_2_-transformed probe signals. Whole genome: the entire set of technically reliable/detected PSVs in the gene array. T*Mtb*-i: the induced genes of THP-1 responding to *Mtb* infection (6). T*Mtb*-iNet: a network-based signature derived from T*Mtb*-i based on STRING protein–protein interaction information (11). T*Mtb*-r: the repressed genes of THP-1 responding to *Mtb* infection (6). In the box plots, the top and bottom of the box represent the first and third quartile, respectively, and the dividing line represents the median; the lowest horizontal bar represents the smallest datum; the highest horizontal bar represents 1.5 times the third quartile. Each data value larger than 1.5 times the third quartile is shown as a dot above the highest bar. The false discovery rate (FDR) of differences between signature PSVs and whole genome PSVs is shown above the 0 h data panel; FDR ≤ 0.05 was regarded as indicating a statistically significant difference.

Figure 1 (also see Figure S1) shows that in uninfected THP-1 cells (i.e., at 0 h), T*Mtb*-i and T*Mtb*-iNet genes displayed significantly lower PSVs than the whole genome genes (false discovery rate, FDR = 0 in each case). The PSVs of the T*Mtb*-r genes at 0 h were not significantly different from the whole genome genes (FDR = 1). Similar phenomena were also observed in another dataset, in which THP-1 cells were infected for 72 h (Figure S2). In summary, T*Mtb*-i and T*Mtb*-iNet genes, as a whole, displayed lower than average PSVs (implying lower/negligible protein abundances) in homogeneous THP-1 cells prior to *Mtb* infection, whereas the T*Mtb*-r genes, as a whole, displayed average PSVs (implying average protein abundances) in the THP-1 cells prior to *Mtb* infection.

### PTBsigUp and PTBsigDn Genes Display Average PSVs (Implying Average Protein Abundances) in the Highly Heterogeneous Whole Blood of HC Donors

After exploring the PSVs of signature genes of T*Mtb*-i, T*Mtb*-iNet, and T*Mtb*-r identified in homogeneous THP-1 cells, we then explored the PSVs of signature genes of PTBsig identified in highly heterogeneous whole blood. This analysis would clarify if signature genes, being divided into up-regulated sub-signature (PTBsigUp) and down-regulated sub-signature (PTBsigDn) genes, displayed similar patterns of PSVs in the heterogeneous whole blood of HC donors as compared to the PSVs of T*Mtb*-i, T*Mtb*-iNet, and T*Mtb*-r genes in homogeneous THP-1 cells.

**Figure 2:**
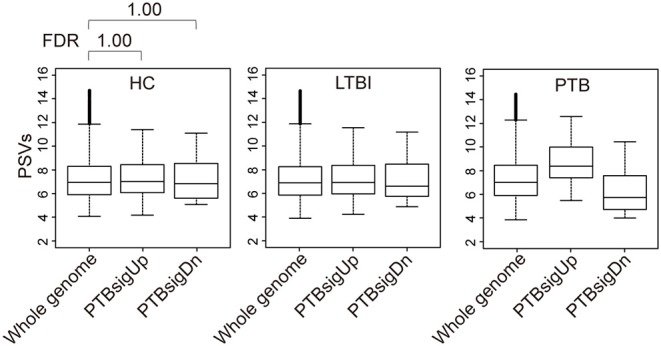
Box plots of the PSVs of PTB-specific signature genes in human whole blood. HC, healthy control donors; LTBI, latent TB infection donors; PTB, pulmonary tuberculosis patients. Whole genome: the entire set of technically reliable/detected PSVs in gene arrays of whole blood. PTBsigUp: the up-regulated genes in the PTB-specific signature PTBsig. PTBsigDn: the down-regulated genes in PTBsig (10). PSV, FDR, and the data plot method are as described in Figure 1.

Figure 2 (also see Figure S3) shows that in whole blood of HC donors, the PSVs of the PTBsigUp and PTBsigDn genes were not significantly different from the PSVs of the filtered whole genome genes (FDR = 1). This finding was cross-validated in an independent dataset (Figure S4). Thus, in contrast to the findings from uninfected THP-1 cells (Figure 1), PTBsigUp and PTBsigDn genes, as a whole, displayed average PSVs (implying average protein abundances) in the whole blood of HC donors. However, whole blood is highly heterogeneous in nucleated cell content with various types of white cells in differing proportions. Accordingly, analysis of PSVs in each of the four main white cell populations separately might reveal differences that were masked in the whole-blood data.

### Separation of HC Blood Into Cell Types Reveals Differences in PTBsigUp and PTBsigDn PSVs Compared to Background Whole Genome PSVs

**Figure 3:**
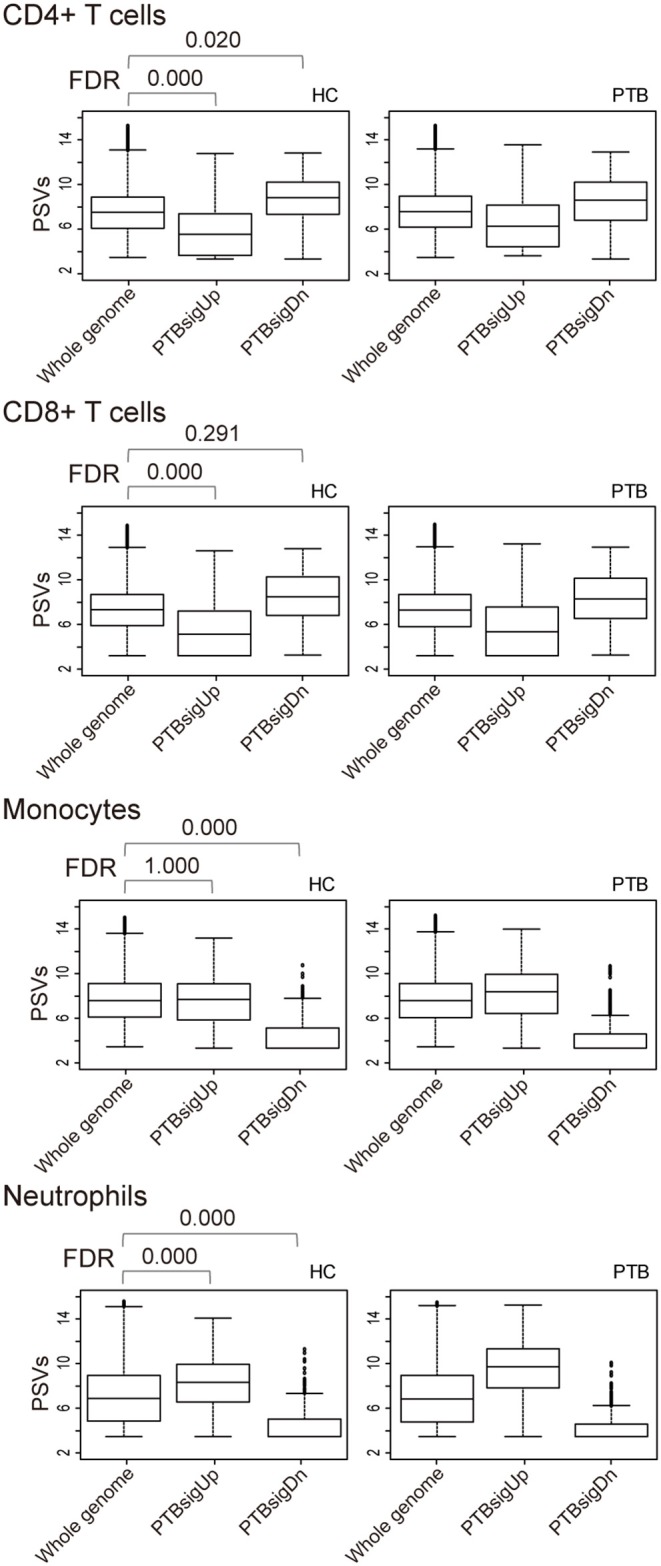
Box plots of the PSVs of PTB-specific signature genes in separated cell populations of human whole blood. HC, healthy control donors; PTB, pulmonary tuberculosis patients. Whole genome, PTBsigUp, and PTBsigDn are as described in Figure 2. PSV, FDR, and the data plot method are as described in Figure 1.

As shown in Figure 3, in comparison to HC donors, the PSVs of PTBsigUp genes in the CD4+ T cells and CD8+ T cells were significantly lower (FDR = 0), whereas the PSVs of PTBsigDn genes in the CD4+ T cells were significantly higher (FDR = 0.020) than the PSVs of the HC filtered whole genome genes. The PSVs of PTBsigDn genes in the CD8+ T cells were not significantly different from the HC whole genome PSVs (FDR = 0.291). In the monocytes, the PSVs of PTBsigUp genes were not significantly different from the PSVs of HC filtered whole genome genes whereas the PSVs of PTBsigDn genes were significantly lower (FDRs = 1 and = 0, respectively). In the neutrophils, the PSVs of PTBsigUp genes were statistically higher than the PSVs of the corresponding HC filtered whole genome genes whereas the PSVs of PTBsigDn genes were statistically lower (both FDRs = 0). The data are available for visual comparison in Figure S5. In summary, some huge and highly significant differences from HC were seen in the PSVs (implying huge differences in protein abundances) of PTBsigUp and PTBsigDn genes within the four different cell populations, differences that were masked in the average PSVs in the whole blood. Notably, neutrophils displayed the highest PTBsigUp PSVs (implying the highest protein abundances) compared to the abundances in the other three kinds of cells.

### The PSV Patterns of *FCGR1* in the Four Cell Populations Are Typical of the PSV Patterns of PTBsigUp Genes in the Four Cell Populations

The up-regulated sub-signature PTBsigUp genes displayed low PSVs in the CD4+ and CD8+ T cells of HC donors, and displayed average and higher PSVs in the monocytes and neutrophils of HC donors, respectively (Figure 3). In the light of this, we aimed to identify from the four cell populations those genes that could be typical representatives of the PSV pattern of PTBsigUp.

**Figure 4:**
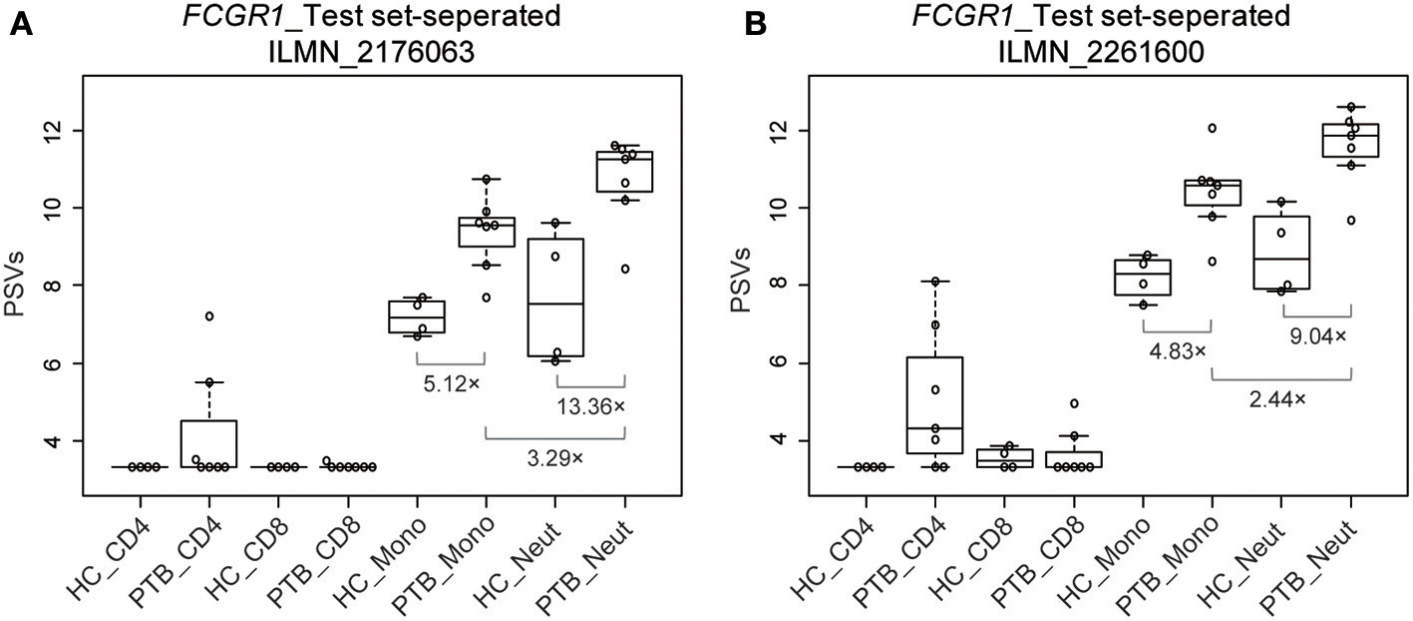
Box plots of the PSVs of *FCGR1* in the separated blood cells. *FCGR1* was revealed by signals from ILMN_2176063 **(A)** and ILMN_2261600 **(B)**. HC_CD4, CD4+ T cells isolated from the whole blood of HC donors; PTB_CD4, CD4+ T cells isolated from the whole blood of PTB patients; other abbreviations are similarly arranged.

We used a combination of three selection criteria: median PSVs in the neutrophils of HC donors (HC_neut) being ≥ 7; log_2_ (fold change) between PTB_neut and HC_neut being ≥ 3; median PSVs of HC_CD4 and HC_CD8 being ≤ 5. By these criteria, we retrieved only two genes, i.e., *FCGR1A* and *FCGR1B*, represented by the two gene-specific DNA fragments of the gene array (i.e., ILMN_2176063 and ILMN_2261600, respectively). Since *FCGR1A, FCGR1B*, and a pseudogene (*FCGR1CP*) are highly homologous (Figure S6), we then aligned the sequences of the two gene-specific DNA fragments of the gene array against all human transcripts through the nucleotide BLAST (blastn) of NCBI. The result showed that the two gene-specific DNA fragments of the gene array were both highly homologous to *FCGR1A, FCGR1B*, and the pseudogene *FCGR1CP* (Tables 1, 2), which indicated that each of the two gene-specific DNA fragments of the gene array actually detected the summed expressions of *FCGR1A, FCGR1B*, and *FCGR1CP* (the three genes together are hereafter termed *FCGR1*). Hence, the two gene-specific DNA fragments of the gene array revealed that *FCGR1* displayed higher PSVs in the whole blood of PTB patients compared to its expression in the whole blood of HC and LTBI donors, and this was markedly reduced during chemotherapy (Figure S7). *FCGR1* displayed low PSVs in the CD4+ and CD8+ T cells from both HC and PTB donors (Figure 4). In contrast, it had higher PSVs in the monocytes and neutrophils of HC donors and was present at much higher levels in the monocytes and neutrophils of PTB patients, especially in neutrophils (i.e., 5.1- and 13.4-fold higher, respectively, for ILMN_2176063; 4.8- and 9.0-fold, respectively, for ILMN_2261600; Figure 4). By implication, there may have been higher expression of the actual receptor on neutrophils than even on monocytes of PTB patients (i.e., 3.29-fold higher for ILMN_2176063 and 2.44-fold for ILMN_2261600). In summary, transcriptome array-based analysis revealed that *FCGR1* can be regarded as a typical representative of PTBsigUp genes in relation to the PSVs patterns in the four cell populations (i.e., CD4+ T cells, CD8+ T cells, monocytes, and neutrophils) and the majority of the receptor may be on neutrophils.

**Table 1:** The sequence alignment of ILMN_2176063 against human transcripts using nucleotide BLAST (blastn) of NCBI.

**Description**	**Identity (%)**	**Accession**
PREDICTED: *Homo sapiens* Fc fragment of IgG receptor Ia (*FCGR1A*), transcript variant X2, mRNA	100.00	XM_005244958.4
PREDICTED: *Homo sapiens* Fc fragment of IgG receptor Ia (*FCGR1A*), transcript variant X1, mRNA	100.00	XM_005244957.3
*Homo sapiens* Fc fragment of IgG receptor Ic, pseudogene (*FCGR1CP*), non-coding RNA	100.00	NR_027484.2
*Homo sapiens* Fc fragment of IgG receptor Ia (*FCGR1A*), mRNA	100.00	NM_000566.3
PREDICTED: *Homo sapiens* Fc fragment of IgG receptor Ib (*FCGR1B*), transcript variant X3, misc_RNA	98.00	XR_001737041.1
PREDICTED: *Homo sapiens* Fc fragment of IgG receptor Ib (*FCGR1B*), transcript variant X2, misc_RNA	98.00	XR_001737040.1
*Homo sapiens* Fc fragment of IgG receptor Ib (*FCGR1B*), transcript variant 4, non-coding RNA	98.00	NR_045213.1
*Homo sapiens* Fc fragment of IgG receptor Ib (*FCGR1B*), transcript variant 3, mRNA	98.00	NM_001244910.1

**Table 2:** The sequence alignment of ILMN_2261600 against human transcripts using nucleotide BLAST (blastn) of NCBI.

**Description**	**Identity (%)**	**Accession**
PREDICTED: *Homo sapiens* Fc fragment of IgG receptor Ia (*FCGR1A*), transcript variant X1, mRNA	100.00	XM_005244957.3
*Homo sapiens* Fc fragment of IgG receptor Ic, pseudogene (*FCGR1CP*), non-coding RNA	100.00	NR_027484.2
*Homo sapiens* Fc fragment of IgG receptor Ib (*FCGR1B*), transcript variant 4, non-coding RNA	100.00	NR_045213.1
*Homo sapiens* Fc fragment of IgG receptor Ib (*FCGR1B*), transcript variant 3, mRNA	100.00	NM_001244910.1
*Homo sapiens* Fc fragment of IgG receptor Ib (*FCGR1B*), transcript variant 1, mRNA	100.00	NM_001017986.3
*Homo sapiens* Fc fragment of IgG receptor Ia (*FCGR1A*), mRNA	100.00	NM_000566.3
PREDICTED: *Homo sapiens* Fc fragment of IgG receptor Ib (*FCGR1B*), transcript variant X3, misc_RNA	98.00	XR_001737041.1
PREDICTED: *Homo sapiens* Fc fragment of IgG receptor Ib (*FCGR1B*), transcript variant X2, misc_RNA	98.00	XR_001737040.1
PREDICTED: *Homo sapiens* Fc fragment of IgG receptor Ib (*FCGR1B*), transcript variant X1, mRNA	98.00	XM_017000661.1

### Confirmation That *FCGR1* Genes (i.e., *FCGR1A, FCGR1B*, and the Pseudogene *FCGR1CP*) Are All Up-Regulated in the Whole Blood of TB Patients

**Figure 5:**
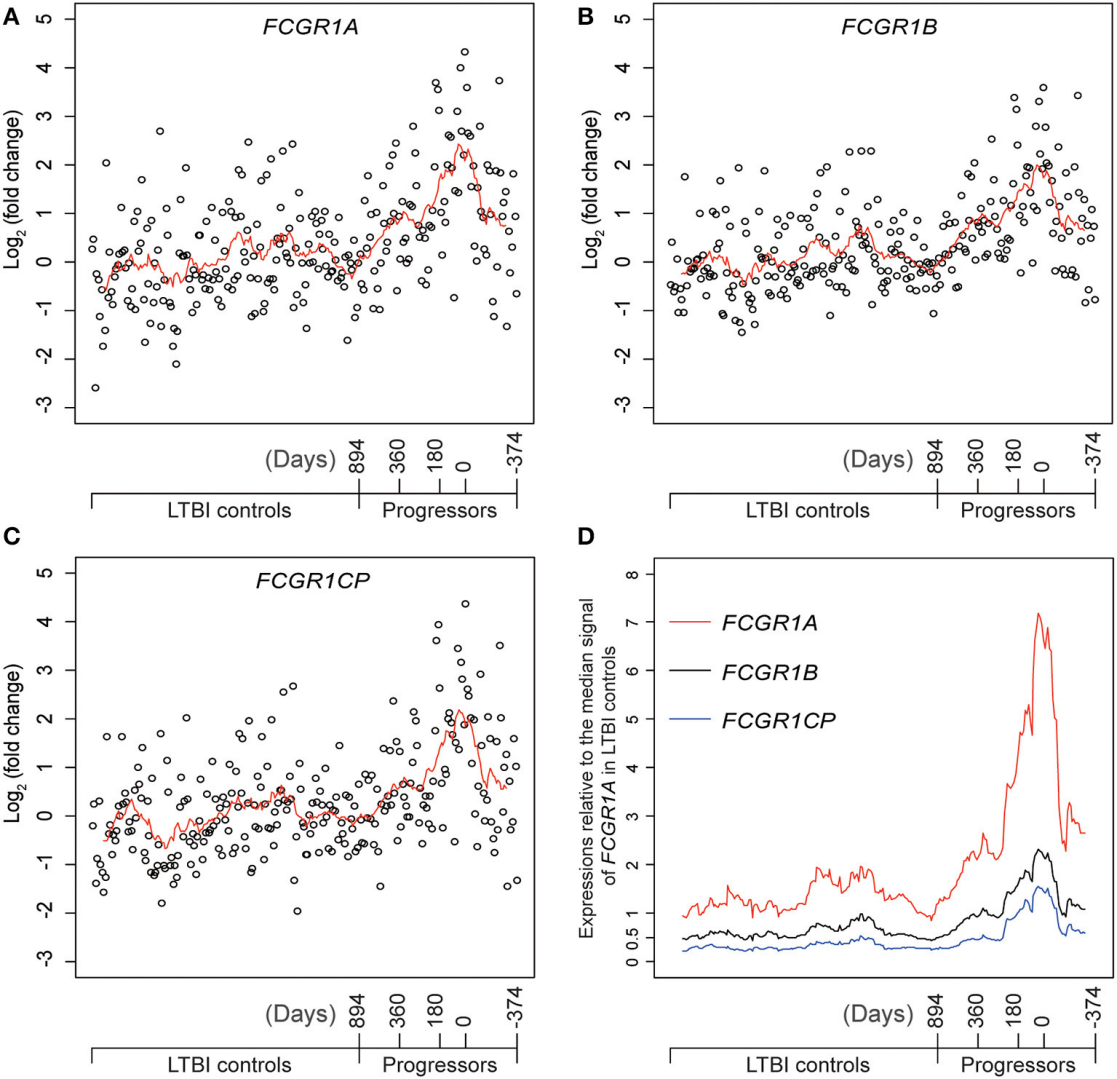
The relative expressions of *FCGR1* in the whole blood of LTBI progressors compared to the whole blood of LTBI controls. **(A–C)** The relative expressions of *FCGR1A, FCGR1B*, and *FCGR1CP*, respectively. Each data point represents the PSV from a single individual: LTBI controls were HC and LTBI non-progressors, plotted in arbitrary sequence from left to right; PSVs from progressors were synchronized to the time of PTB diagnosis, with positive value representing days prior to PTB diagnosis and negative value denoting days post PTB diagnosis. **(D)** The PSVs of *FCGR1A, FCGR1B*, and *FCGR1CP* relative to the median PSV of *FCGR1A* in LTBI controls. The line in each panel represents the moving average trend line with the interval of 15 (i.e., averaging adjacent 15 data points).

Definitive evidence for higher mRNA expression levels of *FCGR1A, FCGR1B*, and the pseudogene *FCGR1CP* in the whole blood of PTB patients than in blood of LTBI and HC donors (Figure S7) was sought by RNA-seq analysis. The promoter sequences of the three genes (from −2,000 bp upstream to 50 bp downstream of the transcription start sites) are highly homologous, indicating that the three genes likely undergo concordant expression in response to a stimulus such as *Mtb* infection (Figure S8). RNA-seq might accurately discriminate differing expression of these highly homologous genes by exploiting differences such as single-nucleotide polymorphisms and/or nucleotide insertion/deletions. For this purpose, we utilized an RNA-seq-based whole blood transcriptome dataset from LTBI donors who had eventually displayed clinical PTB (tagged as progressors) and LTBI donors who did not display clinical PTB (tagged as LTBI controls) (16). As shown in Figures 5A–C and Figure S9, compared to LTBI controls, the expressions of *FCGR1A, FCGR1B*, and *FCGR1CP* were all increasing when progressors were approaching clinical TB and decreasing when progressors were undergoing chemotherapy. In comparison to the median expression in LTBI controls, *FCGR1A* had the highest and *FCGR1CP* had the lowest levels (Figure 5D and Figure S10). In conclusion, all three *FCGR1* genes displayed up-regulation in the whole blood of PTB patients.

## Discussion

This investigation appears to confirm our hypothesis: the customary focus on relative expressions between PSVs in transcriptome arrays, rather than on the absolute values of the PSVs, can lead to loss of meaningful information. Although transcript levels by themselves are not sufficient to predict protein levels in many scenarios (17, 18), they are generally accepted to provide a convenient guide to cell phenotype changes. Accordingly, in interpreting the results, we have extrapolated from PSVs, through implied mRNA levels, to potential protein levels.

The concept that analysis of PSVs could be revelatory was first validated by the re-analysis of the published data from TB-infected THP-1 cell line. As might be expected, significant differences in PSVs were only revealed when the up-regulated and down-regulated signature genes were analyzed separately; the PSV differences otherwise tended to cancel out. The genes that were previously shown to be up-regulated in defining the signature of TB infection (T*Mtb*-i) were here found to be expressed at below-average levels before infection (Figure 1). This was consistent with the mRNA from these signature genes being below the levels needed for translation into protein before infection. For example, *CCL1, IL11, CXCL10, CCL8*, and *CXCL9* are some of these up-regulated signature genes and the cognate proteins in the supernatant of Phorbol 12-myristate 13-acetate (PMA)-differentiated THP-1 cells were barely detectable in a human inflammation antibody array (19). The finding that the mean PSV of the down-regulated signature genes (T*Mtb*-r) was substantially below average after infection suggests that some of these mRNA levels may have dropped below those needed for translation.

In view of the derivation of the PTBsig from whole human blood, it was no surprise to see that the mean PSVs of PTBsigUp and PTBsigDn were significantly different from the average background in PTB (Figure 2). However, much bigger differences, including differences in HC donors, were revealed when data for separate cell types were analyzed (Figure 3). The concealment in whole blood was largely a consequence of the masking effect imposed by differences of opposite polarity in the different cell types. Furthermore, the signatures were evident in HC donors also, where the pattern was similar but of smaller magnitude. The biggest differences were evident in the neutrophils of HC donors, presumably arising through stimulation by cytokines such as IFN-g and G-CSF since un-stimulated neutrophils express little if any surface FCGR1 (20–22). We interpret the presence of the signature in the PSV data from HC subjects as evidence of a background of responses to other infections. This may perhaps limit the practical utility of whole signature PSV data in clinical applications.

However, the selection criteria that we applied to find the most representative PSV signals in the signature led to identification of *FCGR1* as the most robust and analysis of PSVs of the isomers of this gene in the data from separated cell types revealed very large and highly significant differences between HC and PTB datasets (Figure 4). The greatest difference between HC and PTB was in the neutrophil data where, for ILMN_2176063, the PSV of PTB was over 13-fold higher than the PSV of HC. This may point to a practical utility since, even in whole blood data, the PSV of *FCGR1* increased substantially as the status of LTBI transitioned into active PTB disease (Figure 5). It is notable that the up-regulation of *FCGR1A* and/or *FCGR1B* has been repeatedly reported as a component of human TB signatures (23–26). Furthermore, recently, *FCGR1A* was found among blood RNA signatures that prospectively discriminated controllers from progressors early after low-dose *Mtb* infection of cynomolgus macaques (27). However, a caveat is indicated regarding extrapolation from the mRNA and protein levels of FCRG1. The reported *FCGR1A* and/or *FCGR1B* signals from high-throughput approaches/platforms reflect a combination of *FCGR1A, FCGR1B*, and *FCGR1CP* together since they are highly homologous in their promoter regions (Figure S8) and mRNA sequences (Figure S6). Neither mRNA nor protein isomers (Figure S11) were distinguished by the gene arrays or commercial antibodies employed in the various studies. A possible modulatory effect of pseudogene *FCGR1CP* on the final expression of the receptor remains to be explored. Similarly, because there are three highly homologous genes/pseudogenes in the human genome (all located in chromosome 1), whereas there is only one (*Fcgr1* annotated as human *FCGR1A* ortholog) in mouse genome (located in chromosome 3; Table S1), the mouse model is not ideal for precise decipherment of potential functional differences of the three human genes post *Mtb* infection.

Dozens of host-response-based gene signatures, which were identified from human whole blood or peripheral blood mononuclear cells (PBMCs), have been reported to have potential for human TB diagnosis (28). Additional analyses, based on PSVs, could imply/identify the cell types in which the signature genes were sufficiently changed for alteration of cell function. This would aid our limited understanding of TB immunity and immunopathology. It may also guide the cell type-specific implementation of the gene signatures in clinical settings. The role of neutrophils in TB is complex and is likely to vary at different stages of infection and disease (29). Despite their numerical predominance, the functional significance of circulating neutrophils with presumptive high levels of expression of FCGR1 is not known (30). If they reflect the status of neutrophils within TB lesions, then the abundance and high affinity of this IgG receptor may have a key involvement in modulation of innate and acquired immunity in addition to pathology of PTB (31). The predominant infected phagocytes in the airways of TB patients are neutrophils (14) and they are associated with enhanced TB pathology (13). Their FCGR1 receptors may facilitate the phagocytosis that is followed by rapid intracellular replication of *Mtb* and cell necrosis (15). In addition, the cross-linking of the abundant high-affinity receptors by antigen–antibody complexes may generate a cytokine storm that also impairs acquired immunity.

In conclusion, PSVs-based analysis implies an excessive involvement of neutrophil FCGR1 in the impaired balance between protection and pathology in TB.

## Methods

### The Transcriptome Datasets Used in This Study

A total of six publically available gene expression datasets with NCBI GEO (32) accession numbers GSE29628 (6), GSE19439 (10), GSE19443 (10), GSE17477 (33), GSE42830 (34), and GSE79362 (16) were utilized in this study.

GSE29628 contains time-course (i.e., 0, 4, 18, and 48 h) transcriptome data of human macrophage-like cells (THP-1 cells treated with PMA) that had then been infected by the *Mtb* lab strain H37Rv or with one of 11 different *Mtb* W-Beijing strains (6). The array platform of GSE29628 is Affymetrix Human Genome U133 Plus 2.0. Based on the dataset GSE29628, we previously reported a highly prominent induced/up-regulated interferon-related gene signature, termed T*Mtb*-i, in addition to a repressed/down-regulated, function-undefined, minor gene signature, termed T*Mtb*-r. Based on STRING protein–protein interaction information (35), we further refined a network-based signature T*Mtb*-iNet from T*Mtb*-i (11).

GSE19439 contains transcriptome data of whole blood from HC donors, LTBI donors, and PTB patients, who were recruited from London, UK. The array platform of GSE19439 is Illumina HumanHT-12 V3.0 expression beadchip. It served as the training set, and is a SubSeries of SuperSeries GSE19491 (10). Only GSE19439 was utilized in this study because the reported PTB-specific blood signature (PTBsig) was defined from GSE19439, and PTBsig could readily distinguish PTB patients from LTBI and/or HC donors in the other two SubSeries of GSE19491 (which served as test set and validation set).

GSE19443 contains transcriptome data of four cell populations (i.e., CD4+ T cells, CD8+ T cells, monocytes, and neutrophils) separated from whole blood of HC donors and PTB patients who were recruited from London, UK. The array platform of GSE19443 is the same as that of GSE19439 (i.e., Illumina HumanHT-12 V3.0 expression beadchip). GSE19443 served as test set_separated, and is also a SubSeries of SuperSeries GSE19491 (10).

GSE17477 contains the transcriptome data of THP-1 cells, THP-1 cells infected with H37Rv (72 h post-infection), THP-1 cells treated with IFN-γ (2 h), or THP-1 cells infected with H37Rv (72 h post-infection) and then treated with IFN-γ (2 h) (33). The array platform of GSE17477 is Affymetrix Human Genome U133A 2.0, of which the gene-specific DNA fragments are identically represented on Affymetrix Human Genome U133 Plus 2.0 used in GSE29628.

GSE42830 contains the transcriptome data of whole blood from HC donors, TB patients, sarcoid patients, pneumonia patients, or lung cancer patients (34). The platform used for GSE42830 is Illumina HumanHT-12 V4.0 expression beadchip, which shares a majority of gene-specific DNA fragments with the Illumina HumanHT-12 V3.0 expression beadchip used in GSE19439 and GSE19443.

GSE79362 contains RNA-seq-based transcriptome data of whole blood from LTBI controls (people who did not display clinical PTB during the period of investigation after diagnosis) and progressors (LTBI donors who displayed clinical PTB at a later time) (16). See the original research for detailed information of RNA sequencing and the alignment of sequence reads against the human genome (16). The gene expression abundance was monitored based on splice junction counts, which quantify the relative frequency of specific RNA splicing events of expressed genes.

### TB-Relevant Gene Signatures and PSV Analysis

The PSVs of published gene signatures from THP-1 cells (i.e., T*Mtb*-i, T*Mtb*-iNet, T*Mtb*-r) together with the PSVs in control THP-1 cells prior to *Mtb* infection were compared to the PSVs of their cognate filtered whole genome genes in the control samples (6). Likewise, the PSVs of gene signatures PTBsigUp (the up-regulated sub-signature of PTBsig) and PTBsigDn (the down-regulated sub-signature of PTBsig) (12) together with their PSVs in control samples (whole blood of HC donors) were compared to the PSVs of their cognate filtered whole genome genes in control samples (10). Since there might be multiple gene-specific DNA fragments for a single gene in a gene signature, we applied the PSV analysis on the basis of an individual gene-specific DNA fragment for each gene. See the original research (6, 10) for the detailed procedures of normalization of transcriptome data and the generation of filtered whole genome gene data. In brief, THP-1-relevant transcriptome data (i.e., dataset GSE29628) were normalized using Robust Multi-array Averaging (RMA) with quantile normalization in R (Bioconductor) (36). Then, gene-specific DNA fragments that had PSVs consistently below the 95th percentile of all the “Absent” call-flagged signals of the entire dataset were filtered out (6). Illumina BeadStudio software and GeneSpring GX software were utilized for the normalization of whole blood-relevant transcriptome data (i.e., GSE19439) and of the separated cell populations-relevant transcriptome data (i.e., GSE19443). Any PSV <10 was set to 10. A gene-specific DNA fragment was retained when it was called “present” (signal precision <0.01) in >10% of all samples in GSE19439 and had a minimum of 2-fold expression change compared to the median intensity in >10% of all samples in GSE19439 (10).

The dataset GSE17477 (33) was utilized to cross-validate the PSVs of T*Mtb*-i, T*Mtb*-iNet, and T*Mtb*-r genes. The dataset GSE42830 (34) was utilized to cross-validate the PSVs of PTBsigUp and PTBsigDn genes. There are several datasets addressing the transcriptome of THP-1 cells infected with *Mtb* strains (e.g., GSE51029, GSE52819, GSE57028, GSE7870, GSE15539, GSE17477, GSE19052, and GSE6209) and different array platforms were used to generate these datasets. Different array platforms may use different gene-specific DNA sequences to quantify a gene, which might generate platform-specific expression patterns for a gene. To minimize the platform-specific inconsistent expression patterns, we chose the dataset GSE17477, since its array platform was Affymetrix Human Genome U133A 2.0, of which the gene-specific DNA fragments are identically represented on Affymetrix Human Genome U133 Plus 2.0. The Affymetrix Human Genome U133 Plus 2.0 also contains all the gene-specific DNA fragments of Affymetrix Human Genome U133B and many additional gene-specific DNA fragments. The dataset GSE17477 was normalized and filtered exactly the same as for GSE29628 (6). For cross-validating the PSVs of T*Mtb*-i and T*Mtb*-r, we focused on the transcriptome data of THP-1 cells or THP-1 cells infected with H37Rv. The genes of T*Mtb*-i, T*Mtb*-iNet, or T*Mtb*-r could be matched between GSE29628 and GSE17477 through their IDs of gene-specific DNA fragments.

There are a few studies/datasets addressing the transcriptome of PBMCs or whole blood from (pulmonary) TB patients and/or HC donors (1). Of these datasets, only GSE42830 used the Illumina HumanHT-12-relevant platform that contains the transcriptome data of whole blood from both TB patients and HC donors (34), and therefore could be used to validate the PSVs of PTBsigUp and PTBsigDn genes. GSE42830 was normalized and filtered the same way as for GSE19439 (10, 34). For cross-validating the PSVs of PTBsigUp and PTBsigDn genes, we focused on the transcriptome data of whole blood from HC donors and TB patients. The genes of PTBsigUp or PTBsigDn could be matched between GSE19439 and GSE42830 through their IDs of gene-specific DNA fragments.

Also, we retrieved the training set from “GSE79362_primarySampleJunctions.xlsx” and followed the published normalization procedures to normalize the RNA splicing events of expressed genes (16). The expression data of *FCGR1* genes, including *FCGR1A, FCGR1B*, and the pseudogene *FCGR1CP*, were extracted from the normalized training set and their expression in LTBI controls and progressors were analyzed.

### Statistical Analysis

This approach was to test if signature genes, as a whole, displayed lower, higher, or average PSVs, compared to the PSVs of filtered whole genome genes. The reason for applying PSV analysis is that PSVs could broadly reflect the abundances of mRNAs, which in turn are critical determinants of cognate protein abundances. We believe that the information of protein abundance in cells, even before a stimulation (e.g., *Mtb* infection), could imply the degree of functional involvement of that protein, whereas the often adopted relative expression strategy, which just reflects the relative fold change after a stimulation, simply ignores the information of implied protein abundance.

An FDR was defined for assessing the significance of differences (*p*-value) between datasets. FDR was calculated as follows: 10,000 randomly sampled gene sets from cognate filtered whole genome genes with the same set size as the gene signature of interest were generated. Then, the PSVs of each of the 10,000 randomly sampled gene sets were iteratively compared to the PSVs of signature genes using the Kolmogorov–Smirnov test (KS test), a process that generated 10,000 *p*-values. The proportion of *p*-values larger than the empirically determined value 0.0001 in the 10,000 *p*-values was treated as the FDR. If the FDR was equal to or <0.05, then the signature genes' PSVs as a whole were said to be significantly different from the PSVs of the filtered whole genome genes.

## Data Availability Statement

Publicly available datasets were analyzed in this study. This data can be found here: NCBI GEO accession numbers GSE29628, GSE19439, GSE19443, GSE17477, GSE42830, GSE79362.

## Author Contributions

KW, DL, and X-YF conceived and designed the experiments and wrote the paper. KW, DL, ML, and ZC analyzed the data. KW and X-YF interpreted the data. KW acquired the data. DL and X-YF gave overall supervision, critical comments, and reviewed the manuscript. All authors read and approved the final manuscript.

### Conflict of Interest

The authors declare that the research was conducted in the absence of any commercial or financial relationships that could be construed as a potential conflict of interest.
