# Supplementary material for: Probe Signal Values in mRNA Arrays Imply an Excessive Involvement of Neutrophil FCGR1 in Tuberculosis
Source: Front Med (Lausanne). 2020 Feb 14;7:19. doi: 10.3389/fmed.2020.00019 (PMC7033432; doi:10.3389/fmed.2020.00019)
Supplement: Supplementary file 1 [file Data_Sheet_1.pdf]

Supplementary Material

Supplementary Figures

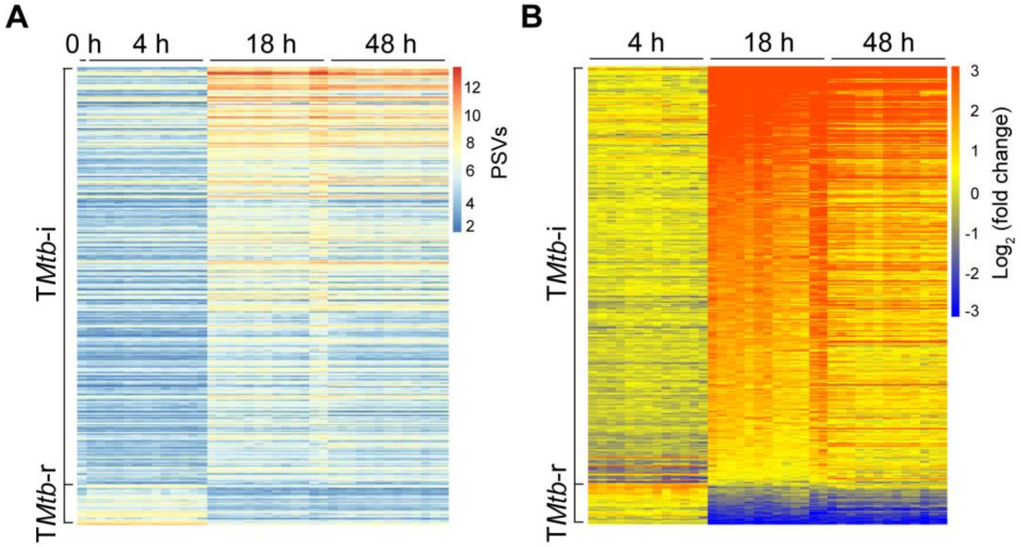

Figure S1: PSVs of the perturbed genes in homogeneous THP-1 cells responding to *Mtb* infection.

(A) The PSVs of T*Mtb*-i and T*Mtb*-r genes.

(B) The relative expression of each gene after *Mtb* infection compared to its expression prior to *Mtb* infection (0 h, panel A). The genes and samples in panel A and panel B are laid out in exactly the same way, except that time point 0 h was omitted in panel B since it served as a control.

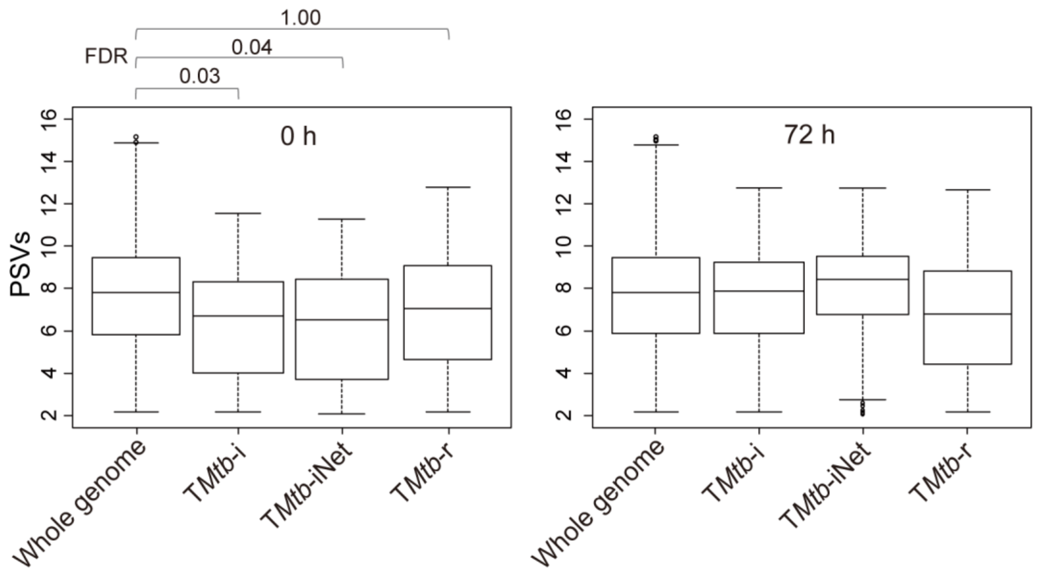

Figure S2: Box plots of the PSVs of THP-1 cells before and 72 h after infection.

T*Mtb*-i data is from matched 212 out of 367 gene-specific DNA fragments, its derived network-based signature T*Mtb*-iNet is from matched 122 out of 165 gene-specific DNA fragments and T*Mtb*-r is from matched 15 out of 32 gene-specific DNA fragments. Data are shown as in Figure 1.

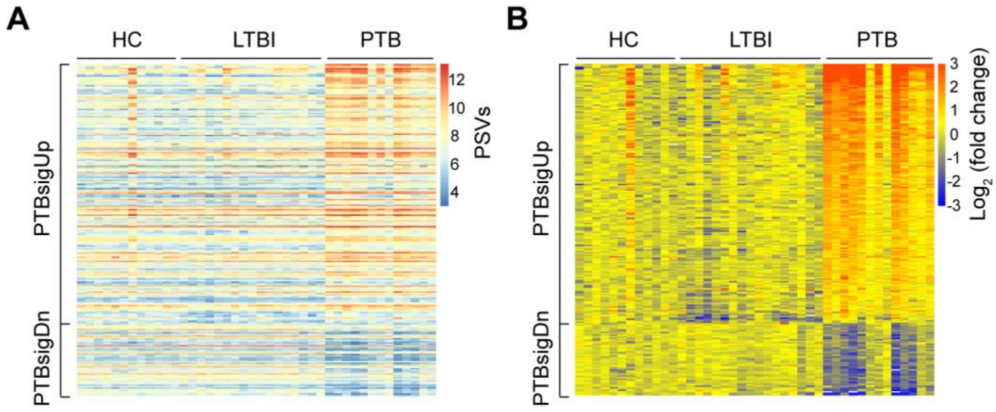

Figure S3: PSVs of PTB-specific signature genes in human whole blood.

(A) PSVs of each gene.

(B) Relative expressions of each gene compared to its median expression level in HC donors.

Data are shown as in Figure S1.

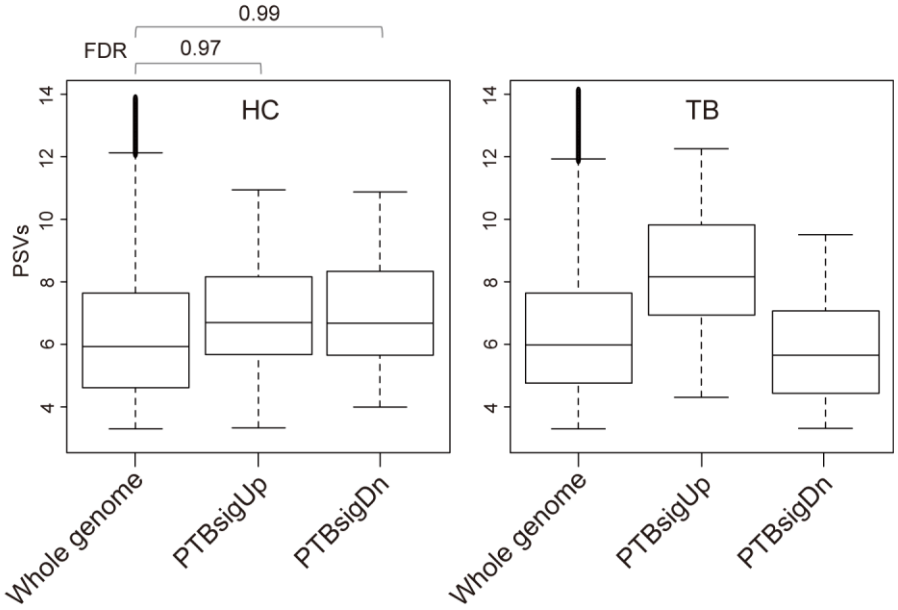

Figure S4: Box plots of the PSVs of the filtered whole genome and signature genes of whole blood.

PTBsigUp data is from matched 302 out of 306 gene-specific DNA fragments and PTBsigDn is from matched 85 out of 87 gene-specific DNA fragments matched between HC donors and TB patients. HC, healthy control donors; TB, tuberculosis patients. The data are shown as in Figure 1.

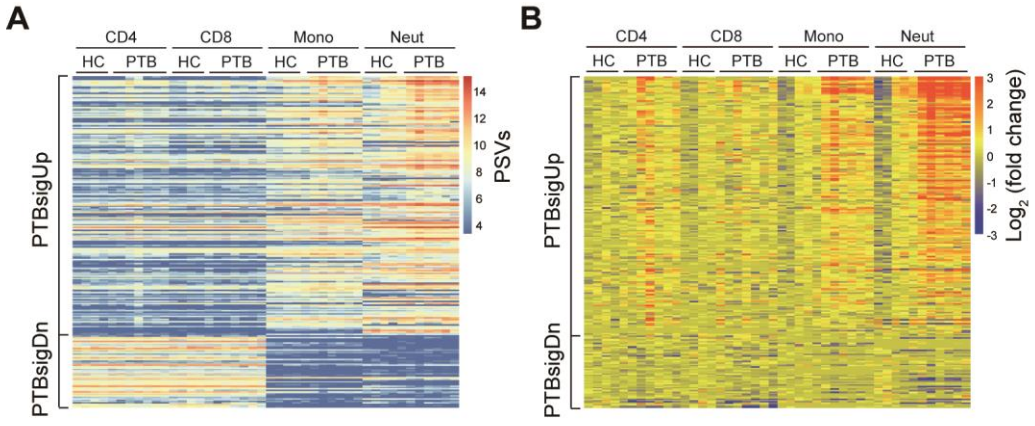

Figure S5: PSVs of PTB-specific signature genes in separated cell populations of human whole blood.

(A) PSVs of each gene.

(B) Relative expressions in different cell populations compared to the cognate median expression level in that cell population from HC donors.

The genes and samples in panel A and panel B are laid out identically.

```
                                                                        Section 1
                           (1) 1           10            20            30            45
    FCGR1A_NM_000566.3     (1) AATATCTTGCATGTTACAGATTTCACTGCTCCCACCAGCTTGGAG
    FCGR1A_XM_005244957.3  (1) AATATCTTGCATGTTACAGATTTCACTGCTCCCACCAGCTTGGAG
    FCGR1A_XM_005244958.4  (1) ---------------------------------------------
    FCGR1B_NM_001244910.1  (1) AATATCTTGCATGTTACAGATTTCACTACTCCCACCAGCTTGGAG
    FCGR1B_NM_001017986.3  (1) AATATCTTGCATGTTACAGATTTCACTACTCCCACCAGCTTGGAG
    FCGR1B_NM_001004340.3  (1) ---------------------------------------------
    FCGR1B_XM_017000662.1  (1) ---------------------------------------------
    FCGR1B_XM_017000661.1  (1) AATATCTTGCATGTTACAGATTTCACTACTCCCACCAGCTTGGAG
    FCGR1B_XR_001737041.1  (1) AATATCTTGCATGTTACAGATTTCACTACTCCCACCAGCTTGGAG
    FCGR1B_XR_001737040.1  (1) AATATCTTGCATGTTACAGATTTCACTACTCCCACCAGCTTGGAG
    FCGR1B_NR_045213.1     (1) AATATCTTGCATGTTACAGATTTCACTACTCCCACCAGCTTGGAG
    FCGR1CP_NR_027484.2    (1) AATATCTTGCATGTTACAGATTTCACTGCTCCCACCAGCTTGGAG
    Consensus              (1) AATATCTTGCATGTTACAGATTTCACTACTCCCACCAGCTTGGAG
                                                                        Section 2
                          (46) 46            60            70            80            90
    FCGR1A_NM_000566.3    (46) ACAACATGTGGTTCTTGACAACTCTGCTCCTTTGGGTTCCAGTTG
    FCGR1A_XM_005244957.3 (46) ACAACATGTGGTTCTTGACAACTCTGCTCCTTTGGGTTCCAGTTG
    FCGR1A_XM_005244958.4  (1) ---------------------------------------------
    FCGR1B_NM_001244910.1 (46) ACAACATGTGGTTCTTGACAACTCTGCTCCTTTGGGTTCCAGTTG
    FCGR1B_NM_001017986.3 (46) ACAACATGTGGTTCTTGACAACTCTGCTCCTTTGGGTTCCAGTTG
    FCGR1B_NM_001004340.3  (1) ---------------------------------------------
    FCGR1B_XM_017000662.1  (1) ---------------------------------------------
    FCGR1B_XM_017000661.1 (46) ACAACATGTGGTTCTTGACAACTCTGCTCCTTTGGGTTCCAGTTG
    FCGR1B_XR_001737041.1 (46) ACAACATGTGGTTCTTGACAACTCTGCTCCTTTGGGTTCCAGTTG
    FCGR1B_XR_001737040.1 (46) ACAACATGTGGTTCTTGACAACTCTGCTCCTTTGGGTTCCAGTTG
    FCGR1B_NR_045213.1    (46) ACAACATGTGGTTCTTGACAACTCTGCTCCTTTGGGTTCCAGTTG
    FCGR1CP_NR_027484.2   (46) ACAACATGTGGTTCTTGACAACTCTGCTCCTTTGGGTTCCAGTTG
    Consensus             (46) ACAACATGTGGTTCTTGACAACTCTGCTCCTTTGGGTTCCAGTTG
                                                                        Section 3
                          (91) 91           100           110           120           135
    FCGR1A_NM_000566.3    (91) ATGGGCAAGTGG---ACACCACAAAGGCAGTGATCACTTTGCAGC
    FCGR1A_XM_005244957.3 (91) ATGGGCAAGTGGTAGACACCACAAAGGCAGTGATCACTTTGCAGC
    FCGR1A_XM_005244958.4  (1) ---------------------------------------------
    FCGR1B_NM_001244910.1 (91) ATGGGCAAGTGG---ACACCACAAAGGCAGTGATCACTTTGCAGC
    FCGR1B_NM_001017986.3 (91) ATGGGCAAGTGG---ACACCACAAAGGCAGTGATCACTTTGCAGC
    FCGR1B_NM_001004340.3  (1) ---------------------------------------------
    FCGR1B_XM_017000662.1  (1) ---------------------------------------------
    FCGR1B_XM_017000661.1 (91) ATGGGCAAGTGGTAGACACCACAAAGGCAGTGATCACTTTGCAGC
    FCGR1B_XR_001737041.1 (91) ATGGGCAAGTGGTAGACACCACAAAGGCAGTGATCACTTTGCAGC
    FCGR1B_XR_001737040.1 (91) ATGGGCAAGTGGTAGACACCACAAAGGCAGTGATCACTTTGCAGC
    FCGR1B_NR_045213.1    (91) ATGGGCAAGTGG---ACACCACAAAGGCAGTGATCACTTTGCAGC
    FCGR1CP_NR_027484.2   (91) ATGGGCAAGTGG---ACACCACAAAGGCAGTGATCACTTTGCAGC
    Consensus             (91) ATGGGCAAGTGG   ACACCACAAAGGCAGTGATCACTTTGCAGC
```

```
                                                                        Section 4
                         (136) 136                150       160       170      180
    FCGR1A_NM_000566.3   (133) CTCCATGGGTCAGCGTGTTCCAAGAGGAAACCGTAACCTTGCACT
  FCGR1A_XM_005244957.3  (136) CTCCATGGGTCAGCGTGTTCCAAGAGGAAACCGTAACCTTGCACT
  FCGR1A_XM_005244958.4    (1) ---------------------------------------------
  FCGR1B_NM_001244910.1  (133) CTCCATGGGTCAGCGTGTTCCAAGAGGAAACCGTAACCTTGCACT
  FCGR1B_NM_001017986.3  (133) CTCCATGGGTCAGCGTGTTCCAAGAGGAAACCGTAACCTTGCACT
  FCGR1B_NM_001004340.3    (1) ---------------------------------------------
  FCGR1B_XM_017000662.1    (1) ---------------------------------------------
  FCGR1B_XM_017000661.1  (136) CTCCATGGGTCAGCGTGTTCCAAGAGGAAACCATAACCTTGCACT
  FCGR1B_XR_001737041.1  (136) CTCCATGGGTCAGCGTGTTCCAAGAGGAAACCATAACCTTGCACT
  FCGR1B_XR_001737040.1  (136) CTCCATGGGTCAGCGTGTTCCAAGAGGAAACCATAACCTTGCACT
     FCGR1B_NR_045213.1  (133) CTCCATGGGTCAGCGTGTTCCAAGAGGAAACCGTAACCTTGCACT
   FCGR1CP_NR_027484.2   (133) CTCCATGGGTCAGCGTGTTCCAAGAGGAAACCGTAACCTTGCACT
              Consensus  (136) CTCCATGGGTCAGCGTGTTCCAAGAGGAAACCGTAACCTTGCACT
                                                                        Section 5
                         (181) 181          190       200       210          225
    FCGR1A_NM_000566.3   (178) GTGAGGTGCTCCATCTGCCTGGGAGCAGCTCTACACAGTGGTTTC
  FCGR1A_XM_005244957.3  (181) GTGAGGTGCTCCATCTGCCTGGGAGCAGCTCTACACAGTGGTTTC
  FCGR1A_XM_005244958.4    (1) ---------------------------------------------
  FCGR1B_NM_001244910.1  (178) GTGAGGTGCTCCATCTGCCTGGGAGCAGCTCCACACAGTGGTTTC
  FCGR1B_NM_001017986.3  (178) GTGAGGTGCTCCATCTGCCTGGGAGCAGCTCCACACAGTGGTTTC
  FCGR1B_NM_001004340.3    (1) ---------------------------------------------
  FCGR1B_XM_017000662.1    (1) ---------------------------------------------
  FCGR1B_XM_017000661.1  (181) GTGAGGTGCTCCATCTGCCTGGGAGCAGCTCCACACAGTGGTTTC
  FCGR1B_XR_001737041.1  (181) GTGAGGTGCTCCATCTGCCTGGGAGCAGCTCCACACAGTGGTTTC
  FCGR1B_XR_001737040.1  (181) GTGAGGTGCTCCATCTGCCTGGGAGCAGCTCCACACAGTGGTTTC
     FCGR1B_NR_045213.1  (178) GTGAGGTGCTCCATCTGCCTGGGAGCAGCTCCACACAGTGGTTTC
   FCGR1CP_NR_027484.2   (178) GTGAGGTGCTCCATCTGCCTGGGAGCAGCTCCACACAGTGGTTTC
              Consensus  (181) GTGAGGTGCTCCATCTGCCTGGGAGCAGCTCCACACAGTGGTTTC
                                                                        Section 6
                         (226) 226                240       250       260      270
    FCGR1A_NM_000566.3   (223) TCAATGGCACAGCCACTCAGACCTCGACCCCCAGCTACAGAATCA
  FCGR1A_XM_005244957.3  (226) TCAATGGCACAGCCACTCAGACCTCGACCCCCAGCTACAGAATCA
  FCGR1A_XM_005244958.4    (1) -----------------------ACCAGCAGAACCTCTTCAAT-A
  FCGR1B_NM_001244910.1  (223) TCAATGGCACAGCCACTCAGACCTCGACCCCCAGCTACAGAATCA
  FCGR1B_NM_001017986.3  (223) TCAATGGCACAGCCACTCAGACCTCGACCCCCAGCTACAGAATCA
  FCGR1B_NM_001004340.3    (1) ----------------------------------------AAT-A
  FCGR1B_XM_017000662.1    (1) ----------------------------------------AAT-A
  FCGR1B_XM_017000661.1  (226) TCAATGGCACAGCCACTCAGACCTCGACCCCCAGCTACAGAATCA
  FCGR1B_XR_001737041.1  (226) TCAATGGCACAGCCACTCAGACCTCGACCCCCAGCTACAGAATCA
  FCGR1B_XR_001737040.1  (226) TCAATGGCACAGCCACTCAGACCTCGACCCCCAGCTACAGAATCA
     FCGR1B_NR_045213.1  (223) TCAATGGCACAGCCACTCAGACCTCGACCCCCAGCTACAGAATCA
   FCGR1CP_NR_027484.2   (223) TCAATGGCACAGCCACTCAGACCTCGACCCCCAGCTACAGAATCA
              Consensus  (226) TCAATGGCACAGCCACTCAGACCTCGACCCCCAGCTACAGAATCA
```

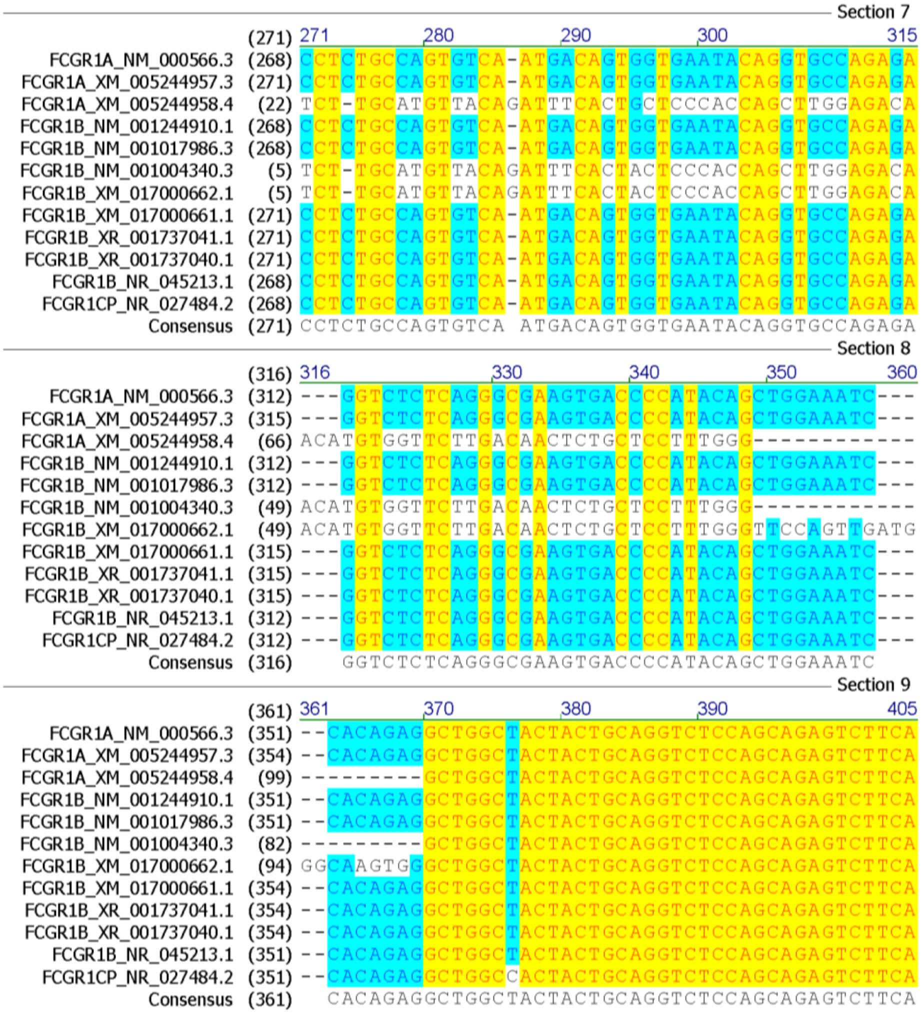
Section 7
(271) 271 280 290 300 315
FCGR1A_NM_000566.3 (268) CCTCTGCCAGTGTCA-ATGACAGTGGTGAATACAGGTGCCAGAGA
FCGR1A_XM_005244957.3 (271) CCTCTGCCAGTGTCA-ATGACAGTGGTGAATACAGGTGCCAGAGA
FCGR1A_XM_005244958.4 (22) TCT-TGCATGTTACAGATTTCACTGCTCCCACCAGCTTGGAGACA
FCGR1B_NM_001244910.1 (268) CCTCTGCCAGTGTCA-ATGACAGTGGTGAATACAGGTGCCAGAGA
FCGR1B_NM_001017986.3 (268) CCTCTGCCAGTGTCA-ATGACAGTGGTGAATACAGGTGCCAGAGA
FCGR1B_NM_001004340.3 (5) TCT-TGCATGTTACAGATTTCACTACTCCCACCAGCTTGGAGACA
FCGR1B_XM_017000662.1 (5) TCT-TGCATGTTACAGATTTCACTACTCCCACCAGCTTGGAGACA
FCGR1B_XM_017000661.1 (271) CCTCTGCCAGTGTCA-ATGACAGTGGTGAATACAGGTGCCAGAGA
FCGR1B_XR_001737041.1 (271) CCTCTGCCAGTGTCA-ATGACAGTGGTGAATACAGGTGCCAGAGA
FCGR1B_XR_001737040.1 (271) CCTCTGCCAGTGTCA-ATGACAGTGGTGAATACAGGTGCCAGAGA
FCGR1B_NR_045213.1 (268) CCTCTGCCAGTGTCA-ATGACAGTGGTGAATACAGGTGCCAGAGA
FCGR1CP_NR_027484.2 (268) CCTCTGCCAGTGTCA-ATGACAGTGGTGAATACAGGTGCCAGAGA
Consensus (271) CCTCTGCCAGTGTCA ATGACAGTGGTGAATACAGGTGCCAGAGA
Section 8
(316) 316 330 340 350 360
FCGR1A_NM_000566.3 (312) ---GGTCTCTCAGGGCGAAGTGACCCCATACAGCTGGAAATC---
FCGR1A_XM_005244957.3 (315) ---GGTCTCTCAGGGCGAAGTGACCCCATACAGCTGGAAATC---
FCGR1A_XM_005244958.4 (66) ACATGTGGTTCTTGACAACTCTGCTCCTTTGGG------------
FCGR1B_NM_001244910.1 (312) ---GGTCTCTCAGGGCGAAGTGACCCCATACAGCTGGAAATC---
FCGR1B_NM_001017986.3 (312) ---GGTCTCTCAGGGCGAAGTGACCCCATACAGCTGGAAATC---
FCGR1B_NM_001004340.3 (49) ACATGTGGTTCTTGACAACTCTGCTCCTTTGGG------------
FCGR1B_XM_017000662.1 (49) ACATGTGGTTCTTGACAACTCTGCTCCTTTGGGTTCCAGTTGATG
FCGR1B_XM_017000661.1 (315) ---GGTCTCTCAGGGCGAAGTGACCCCATACAGCTGGAAATC---
FCGR1B_XR_001737041.1 (315) ---GGTCTCTCAGGGCGAAGTGACCCCATACAGCTGGAAATC---
FCGR1B_XR_001737040.1 (315) ---GGTCTCTCAGGGCGAAGTGACCCCATACAGCTGGAAATC---
FCGR1B_NR_045213.1 (312) ---GGTCTCTCAGGGCGAAGTGACCCCATACAGCTGGAAATC---
FCGR1CP_NR_027484.2 (312) ---GGTCTCTCAGGGCGAAGTGACCCCATACAGCTGGAAATC---
Consensus (316) GGTCTCTCAGGGCGAAGTGACCCCATACAGCTGGAAATC
Section 9
(361) 361 370 380 390 405
FCGR1A_NM_000566.3 (351) --CACAGAGGCTGGCTACTACTGCAGGTCTCCAGCAGAGTCTTCA
FCGR1A_XM_005244957.3 (354) --CACAGAGGCTGGCTACTACTGCAGGTCTCCAGCAGAGTCTTCA
FCGR1A_XM_005244958.4 (99) ---------GCTGGCTACTACTGCAGGTCTCCAGCAGAGTCTTCA
FCGR1B_NM_001244910.1 (351) --CACAGAGGCTGGCTACTACTGCAGGTCTCCAGCAGAGTCTTCA
FCGR1B_NM_001017986.3 (351) --CACAGAGGCTGGCTACTACTGCAGGTCTCCAGCAGAGTCTTCA
FCGR1B_NM_001004340.3 (82) ---------GCTGGCTACTACTGCAGGTCTCCAGCAGAGTCTTCA
FCGR1B_XM_017000662.1 (94) GGCAAGTGGGCTGGCTACTACTGCAGGTCTCCAGCAGAGTCTTCA
FCGR1B_XM_017000661.1 (354) --CACAGAGGCTGGCTACTACTGCAGGTCTCCAGCAGAGTCTTCA
FCGR1B_XR_001737041.1 (354) --CACAGAGGCTGGCTACTACTGCAGGTCTCCAGCAGAGTCTTCA
FCGR1B_XR_001737040.1 (354) --CACAGAGGCTGGCTACTACTGCAGGTCTCCAGCAGAGTCTTCA
FCGR1B_NR_045213.1 (351) --CACAGAGGCTGGCTACTACTGCAGGTCTCCAGCAGAGTCTTCA
FCGR1CP_NR_027484.2 (351) --CACAGAGGCTGGCCACTACTGCAGGTCTCCAGCAGAGTCTTCA
Consensus (361) CACAGAGGCTGGCTACTACTGCAGGTCTCCAGCAGAGTCTTCA

```
                                                                    Section 10
                         (406) 406                  420          430          440         450
    FCGR1A_NM_000566.3   (394) CGGAAGGAGAACCTCTGGCCTTGAGGTGTCATGCGTGGAAGGATA
  FCGR1A_XM_005244957.3  (397) CGGAAGGAGAACCTCTGGCCTTGAGGTGTCATGCGTGGAAGGATA
  FCGR1A_XM_005244958.4  (135) CGGAAGGAGAACCTCTGGCCTTGAGGTGTCATGCGTGGAAGGATA
  FCGR1B_NM_001244910.1  (394) TGGAAGGAGAACCTCTGGCCTTGAGGTGTCATGCGTGGAAGGATA
  FCGR1B_NM_001017986.3  (394) TGGAAGGAGAACCTCTGGCCTTGAGGTGTCATGCGTGGAAGGATA
  FCGR1B_NM_001004340.3  (118) TGGAAGGAGAACCTCTGGCCTTGAGGTGTCATGCGTGGAAGGATA
  FCGR1B_XM_017000662.1  (139) TGGAAGGAGAACCTCTGGCCTTGAGGTGTCATGCGTGGAAGGATA
  FCGR1B_XM_017000661.1  (397) TGGAAGGAGAACCTCTGGCCTTGAGGTGTCATGCGTGGAAGGATA
  FCGR1B_XR_001737041.1  (397) TGGAAGGAGAACCTCTGGCCTTGAGGTGTCATGCGTGGAAGGATA
  FCGR1B_XR_001737040.1  (397) TGGAAGGAGAACCTCTGGCCTTGAGGTGTCATGCGTGGAAGGATA
     FCGR1B_NR_045213.1  (394) TGGAAGGAGAACCTCTGGCCTTGAGGTGTCATGCGTGGAAGGATA
    FCGR1CP_NR_027484.2  (394) CGGAAGGAGAACCTCTGGCCTTGAGGTGTCATGCGTGGAAGGATA
              Consensus  (406) TGGAAGGAGAACCTCTGGCCTTGAGGTGTCATGCGTGGAAGGATA
                                                                    Section 11
                         (451) 451          460          470          480            495
    FCGR1A_NM_000566.3   (439) AGCTGGTGTACAATGTGCTTTACTATCGAAATGGCAAAGCCTTTA
  FCGR1A_XM_005244957.3  (442) AGCTGGTGTACAATGTGCTTTACTATCGAAATGGCAAAGCCTTTA
  FCGR1A_XM_005244958.4  (180) AGCTGGTGTACAATGTGCTTTACTATCGAAATGGCAAAGCCTTTA
  FCGR1B_NM_001244910.1  (439) AGCTGGTGTACAATGTGCTTTACTATCGAAATGGCAAAGCCTTTA
  FCGR1B_NM_001017986.3  (439) AGCTGGTGTACAATGTGCTTTACTATCGAAATGGCAAAGCCTTTA
  FCGR1B_NM_001004340.3  (163) AGCTGGTGTACAATGTGCTTTACTATCGAAATGGCAAAGCCTTTA
  FCGR1B_XM_017000662.1  (184) AGCTGGTGTACAATGTGCTTTACTATCGAAATGGCAAAGCCTTTA
  FCGR1B_XM_017000661.1  (442) AGCTGGTGTACAATGTGCTTTACTATCGAAATGGCAAAGCCTTTA
  FCGR1B_XR_001737041.1  (442) AGCTGGTGTACAATGTGCTTTACTATCGAAATGGCAAAGCCTTTA
  FCGR1B_XR_001737040.1  (442) AGCTGGTGTACAATGTGCTTTACTATCGAAATGGCAAAGCCTTTA
     FCGR1B_NR_045213.1  (439) AGCTGGTGTACAATGTGCTTTACTATCGAAATGGCAAAGCCTTTA
    FCGR1CP_NR_027484.2  (439) AGCTGGTGTACAATGTGCTTTACTATCGAAATGGCAAAGCCTTTA
              Consensus  (451) AGCTGGTGTACAATGTGCTTTACTATCGAAATGGCAAAGCCTTTA
                                                                    Section 12
                         (496) 496                  510          520          530         540
    FCGR1A_NM_000566.3   (484) AGTTTTTCCACTGGAATTCTAACCTCACCATTCTGAAAACCAACA
  FCGR1A_XM_005244957.3  (487) AGTTTTTCCACTGGAATTCTAACCTCACCATTCTGAAAACCAACA
  FCGR1A_XM_005244958.4  (225) AGTTTTTCCACTGGAATTCTAACCTCACCATTCTGAAAACCAACA
  FCGR1B_NM_001244910.1  (484) AGTTTTTCCACTGGAATTCTAACCTCACCATTCTGAAAACCAACA
  FCGR1B_NM_001017986.3  (484) AGTTTTTCCACTGGAATTCTAACCTCACCATTCTGAAAACCAACA
  FCGR1B_NM_001004340.3  (208) AGTTTTTCCACTGGAATTCTAACCTCACCATTCTGAAAACCAACA
  FCGR1B_XM_017000662.1  (229) AGTTTTTCCACTGGAATTCTAACCTCACCATTCTGAAAACCAACA
  FCGR1B_XM_017000661.1  (487) AGTTTTTCCACTGGAATTCTAACCTCACCATTCTGAAAACCAACA
  FCGR1B_XR_001737041.1  (487) AGTTTTTCCACTGGAATTCTAACCTCACCATTCTGAAAACCAACA
  FCGR1B_XR_001737040.1  (487) AGTTTTTCCACTGGAATTCTAACCTCACCATTCTGAAAACCAACA
     FCGR1B_NR_045213.1  (484) AGTTTTTCCACTGGAATTCTAACCTCACCATTCTGAAAACCAACA
    FCGR1CP_NR_027484.2  (484) AGTTTTTCCACTGGAATTCTAACCTCACCATTCTGAAAACCAACA
              Consensus  (496) AGTTTTTCCACTGGAATTCTAACCTCACCATTCTGAAAACCAACA
```

```
                                                                              Section 13
                            (541) 541         550         560         570            585
FCGR1A_NM_000566.3          (529) TAAGTCACAATGGCACCTACCATTGCTCAGGCATGGGAAAGCATC
FCGR1A_XM_005244957.3       (532) TAAGTCACAATGGCACCTACCATTGCTCAGGCATGGGAAAGCATC
FCGR1A_XM_005244958.4       (270) TAAGTCACAATGGCACCTACCATTGCTCAGGCATGGGAAAGCATC
FCGR1B_NM_001244910.1       (529) TAAGTCACAATGGCACCTACCATTGCTCAGGCATGGGAAAGCATC
FCGR1B_NM_001017986.3       (529) TAAGTCACAATGGCACCTACCATTGCTCAGGCATGGGAAAGCATC
FCGR1B_NM_001004340.3       (253) TAAGTCACAATGGCACCTACCATTGCTCAGGCATGGGAAAGCATC
FCGR1B_XM_017000662.1       (274) TAAGTCACAATGGCACCTACCATTGCTCAGGCATGGGAAAGCATC
FCGR1B_XM_017000661.1       (532) TAAGTCACAATGGCACCTACCATTGCTCAGGCATGGGAAAGCATC
FCGR1B_XR_001737041.1       (532) TAAGTCACAATGGCACCTACCATTGCTCAGGCATGGGAAAGCATC
FCGR1B_XR_001737040.1       (532) TAAGTCACAATGGCACCTACCATTGCTCAGGCATGGGAAAGCATC
FCGR1B_NR_045213.1          (529) TAAGTCACAATGGCACCTACCATTGCTCAGGCATGGGAAAGCATC
FCGR1CP_NR_027484.2         (529) TAAGTCACAATGGCACCTACCATTGCTCAGGCAAGGGAAAGCATC
Consensus                   (541) TAAGTCACAATGGCACCTACCATTGCTCAGGCATGGGAAAGCATC
                                                                              Section 14
                            (586) 586           600         610         620        630
FCGR1A_NM_000566.3          (574) GCTACACATCAGCAGGAATATCTGT---CACTGTGAAAGAGCTAT
FCGR1A_XM_005244957.3       (577) GCTACACATCAGCAGGAATATCTGT---CACTGTGAAAGAGCTAT
FCGR1A_XM_005244958.4       (315) GCTACACATCAGCAGGAATATCTGT---CACTGTGAAAGAGCTAT
FCGR1B_NM_001244910.1       (574) GCTACACATCAGCAGGAATATCACAATACACTGTGAAAGAGCTAT
FCGR1B_NM_001017986.3       (574) GCTACACATCAGCAGGAATATCACAATACACTGTGAAAG------
FCGR1B_NM_001004340.3       (298) GCTACACATCAGCAGGAATATCACAATACACTGTGAAAG------
FCGR1B_XM_017000662.1       (319) GCTACACATCAGCAGGAATATCACAATACACTGTGAAAG------
FCGR1B_XM_017000661.1       (577) GCTACACATCAGCAGGAATATCACAATACACTGTGAAAG------
FCGR1B_XR_001737041.1       (577) GCTACACATCAGCAGGAATATCACAATACACTGTGAAAGAGCTAT
FCGR1B_XR_001737040.1       (577) GCTACACATCAGCAGGAATATCACAATACACTGTGAAAGAGCTAT
FCGR1B_NR_045213.1          (574) GCTACACATCAGCAGGAATATCACAATACACTGTGAAAGAGCTAT
FCGR1CP_NR_027484.2         (574) ACTACACATCAGCAGGAATATCACAATACACTGTGAAAGAGCTAT
Consensus                   (586) GCTACACATCAGCAGGAATATCACAATACACTGTGAAAGAGCTAT
                                                                              Section 15
                            (631) 631         640         650         660            675
FCGR1A_NM_000566.3          (616) TTCCAGCTCCAGTGCTGAATGCATCTGTGACATCCCCACTCCTGG
FCGR1A_XM_005244957.3       (619) TTCCAGCTCCAGTGCTGAATGCATCTGTGACATCCCCACTCCTGG
FCGR1A_XM_005244958.4       (357) TTCCAGCTCCAGTGCTGAATGCATCTGTGACATCCCCACTCCTGG
FCGR1B_NM_001244910.1       (619) TTCCAGCTCCAGTGCTGAATGCATCTGTGACATCCCCACTCCTGG
FCGR1B_NM_001017986.3       (613) ---------------------------------------------
FCGR1B_NM_001004340.3       (337) ---------------------------------------------
FCGR1B_XM_017000662.1       (358) ---------------------------------------------
FCGR1B_XM_017000661.1       (616) ---------------------------------------------
FCGR1B_XR_001737041.1       (622) TTCCAGCTCCAGTGCTGAATGCATCTGTGACATCCCCACTCCTGG
FCGR1B_XR_001737040.1       (622) TTCCAGCTCCAGTGCTGAATGCATCTGTGACATCCCCACTCCTGG
FCGR1B_NR_045213.1          (619) TTCCAGCTCCAGTGCTGAATGCATCTGTGACATCCCCACTCCTGG
FCGR1CP_NR_027484.2         (619) TTCCAGCTCCAGTGCTGAATGCATCTGTGACATCCCCACTCCTGG
Consensus                   (631) TTCCAGCTCCAGTGCTGAATGCATCTGTGACATCCCCACTCCTGG
```

```
                                                                  Section 16
                          (676) 676          690       700       710       720
    FCGR1A_NM_000566.3    (661) AGGGGAATCTGGTCACCCTGAGCTGTGAAACAAAGTTGCTCTTGC
    FCGR1A_XM_005244957.3 (664) AGGGGAATCTGGTCACCCTGAGCTGTGAAACAAAGTTGCTCTTGC
    FCGR1A_XM_005244958.4 (402) AGGGGAATCTGGTCACCCTGAGCTGTGAAACAAAGTTGCTCTTGC
    FCGR1B_NM_001244910.1 (664) AGGGGAATCTGGTCACCCTGAGCTGTGAAACAAAGTTGCTCTTGC
    FCGR1B_NM_001017986.3 (613) ---------------------------------------------
    FCGR1B_NM_001004340.3 (337) ---------------------------------------------
    FCGR1B_XM_017000662.1 (358) ---------------------------------------------
    FCGR1B_XM_017000661.1 (616) ---------------------------------------------
    FCGR1B_XR_001737041.1 (667) AGGGGAATCTGGTCACCCTGAGCTGTGAAACAAAGTTGCTCTTGC
    FCGR1B_XR_001737040.1 (667) AGGGGAATCTGGTCACCCTGAGCTGTGAAACAAAGTTGCTCTTGC
    FCGR1B_NR_045213.1    (664) AGGGGAATCTGGTCACCCTGAGCTGTGAAACAAAGTTGCTCTTGC
    FCGR1CP_NR_027484.2   (664) -GGGGAATCTGGTCACCCTGAGCTGTGAAACAAAGTTGCTCTTGC
    Consensus             (676) AGGGGAATCTGGTCACCCTGAGCTGTGAAACAAAGTTGCTCTTGC
                                                                  Section 17
                          (721) 721      730       740       750            765
    FCGR1A_NM_000566.3    (706) AGAGGCCTGGTTTGCAGCTTTACTTCTCCTTCTACATGGGCAGCA
    FCGR1A_XM_005244957.3 (709) AGAGGCCTGGTTTGCAGCTTTACTTCTCCTTCTACATGGGCAGCA
    FCGR1A_XM_005244958.4 (447) AGAGGCCTGGTTTGCAGCTTTACTTCTCCTTCTACATGGGCAGCA
    FCGR1B_NM_001244910.1 (709) AGAGGCCTGGTTTGTAGCTTTACTTCTCCTTCTACATGGGCAGCA
    FCGR1B_NM_001017986.3 (613) ---------------------------------------------
    FCGR1B_NM_001004340.3 (337) ---------------------------------------------
    FCGR1B_XM_017000662.1 (358) ---------------------------------------------
    FCGR1B_XM_017000661.1 (616) ---------------------------------------------
    FCGR1B_XR_001737041.1 (712) AGAGGCCTGGTTTGTAGCTTTACTTCTCCTTCTACATGGGCAGCA
    FCGR1B_XR_001737040.1 (712) AGAGGCCTGGTTTGTAGCTTTACTTCTCCTTCTACATGGGCAGCA
    FCGR1B_NR_045213.1    (709) AGAGGCCTGGTTTGTAGCTTTACTTCTCCTTCTACATGGGCAGCA
    FCGR1CP_NR_027484.2   (708) AGAGGCCTGGTTTGCAGCTTTACTTCTCCTTCTACATGGGCAGCA
    Consensus             (721) AGAGGCCTGGTTTG AGCTTTACTTCTCCTTCTACATGGGCAGCA
                                                                  Section 18
                          (766) 766          780       790       800       810
    FCGR1A_NM_000566.3    (751) AGACCCTGCGAGGCAGGAACACATCCTCTGAATACCAAATACTAA
    FCGR1A_XM_005244957.3 (754) AGACCCTGCGAGGCAGGAACACATCCTCTGAATACCAAATACTAA
    FCGR1A_XM_005244958.4 (492) AGACCCTGCGAGGCAGGAACACATCCTCTGAATACCAAATACTAA
    FCGR1B_NM_001244910.1 (754) AGACCCTGCGAGGCAGGAACACATCCTCTGAATACCAAATACTAA
    FCGR1B_NM_001017986.3 (613) ---------------------------------------------
    FCGR1B_NM_001004340.3 (337) ---------------------------------------------
    FCGR1B_XM_017000662.1 (358) ---------------------------------------------
    FCGR1B_XM_017000661.1 (616) ---------------------------------------------
    FCGR1B_XR_001737041.1 (757) AGACCCTGCGAGGCAGGAACACATCCTCTGAATACCAAATACTAA
    FCGR1B_XR_001737040.1 (757) AGACCCTGCGAGGCAGGAACACATCCTCTGAATACCAAATACTAA
    FCGR1B_NR_045213.1    (754) AGACCCTGCGAGGCAGGAACACATCCTCTGAATACCAAATACTAA
    FCGR1CP_NR_027484.2   (753) AGACCCTGCGAGGCAGGAACACATCCTCTGAATACTAAATACTAA
    Consensus             (766) AGACCCTGCGAGGCAGGAACACATCCTCTGAATACCAAATACTAA
```

```
                                                                    Section 19
                        (811) 811         820       830       840           855
FCGR1A_NM_000566.3      (796) CTGCTAGAAGAGAAGACTCTGGGTTATACTGGTGCGAGGCTGCCA
FCGR1A_XM_005244957.3   (799) CTGCTAGAAGAGAAGACTCTGGGTTATACTGGTGCGAGGCTGCCA
FCGR1A_XM_005244958.4   (537) CTGCTAGAAGAGAAGACTCTGGGTTATACTGGTGCGAGGCTGCCA
FCGR1B_NM_001244910.1   (799) CTGCTAGAAGAGAAGACTCTGGGTTATACTGGTGCGAGGCTGCCA
FCGR1B_NM_001017986.3   (613) ---------------------------------------------
FCGR1B_NM_001004340.3   (337) ---------------------------------------------
FCGR1B_XM_017000662.1   (358) ---------------------------------------------
FCGR1B_XM_017000661.1   (616) ---------------------------------------------
FCGR1B_XR_001737041.1   (802) CTGCTAGAAGAGAAGACTCTGGGTTATACTGGTGCGAGGCTGCCA
FCGR1B_XR_001737040.1   (802) CTGCTAGAAGAGAAGACTCTGGGTTATACTGGTGCGAGGCTGCCA
FCGR1B_NR_045213.1      (799) CTGCTAGAAGAGAAGACTCTGGGTTATACTGGTGCGAGGCTGCCA
FCGR1CP_NR_027484.2     (798) CTGCTAGAAGAGAAGACTCTGGGTTATACTGGTGCGAGGCTGCCA
Consensus               (811) CTGCTAGAAGAGAAGACTCTGGGTTATACTGGTGCGAGGCTGCCA
                                                                    Section 20
                        (856) 856           870       880       890       900
FCGR1A_NM_000566.3      (841) CAGAGGATGGAAATGTCCTTAAGCGCAGCCCTGAGTTGGAGCTTC
FCGR1A_XM_005244957.3   (844) CAGAGGATGGAAATGTCCTTAAGCGCAGCCCTGAGTTGGAGCTTC
FCGR1A_XM_005244958.4   (582) CAGAGGATGGAAATGTCCTTAAGCGCAGCCCTGAGTTGGAGCTTC
FCGR1B_NM_001244910.1   (844) CAGAGGATGGAAATGTCCTTAAGCACAGCCCTGAGTTGGAGCTTC
FCGR1B_NM_001017986.3   (613) ---------------------------------------------
FCGR1B_NM_001004340.3   (337) ---------------------------------------------
FCGR1B_XM_017000662.1   (358) ---------------------------------------------
FCGR1B_XM_017000661.1   (616) ---------------------------------------------
FCGR1B_XR_001737041.1   (847) CAGAGGATGGAAATGTCCTTAAGCACAGCCCTGAGTTGGAGCTTC
FCGR1B_XR_001737040.1   (847) CAGAGGATGGAAATGTCCTTAAGCACAGCCCTGAGTTGGAGCTTC
FCGR1B_NR_045213.1      (844) CAGAGGATGGAAATGTCCTTAAGCACAGCCCTGAGTTGGAGCTTC
FCGR1CP_NR_027484.2     (843) CAGAGGATGGAAATGTCCTTAAGCGCAGCCCTGAGTTGGAGCTTC
Consensus               (856) CAGAGGATGGAAATGTCCTTAAGC CAGCCCTGAGTTGGAGCTTC
                                                                    Section 21
                        (901) 901         910       920       930           945
FCGR1A_NM_000566.3      (886) AAGTGCTTG------------------------------------
FCGR1A_XM_005244957.3   (889) AAGTGCTTG------------------------------------
FCGR1A_XM_005244958.4   (627) AAGTGCTTG------------------------------------
FCGR1B_NM_001244910.1   (889) AAGTGCTTGGTGAGAATGACGGGAAGCCACTGGCACAGAAGAAGG
FCGR1B_NM_001017986.3   (613) ---------------------------------------------
FCGR1B_NM_001004340.3   (337) ---------------------------------------------
FCGR1B_XM_017000662.1   (358) ---------------------------------------------
FCGR1B_XM_017000661.1   (616) ---------------------------------------------
FCGR1B_XR_001737041.1   (892) AAGTGCTTGGTGAGAATGACGGGAAGCCACTGGCACAGAAGAAGG
FCGR1B_XR_001737040.1   (892) AAGTGCTTG------------------------------------
FCGR1B_NR_045213.1      (889) AAGTGCTTG------------------------------------
FCGR1CP_NR_027484.2     (888) AAGTGCTTG------------------------------------
Consensus               (901) AAGTGCTTG
```

```
                                                                              Section 22
                          (946) 946           960       970       980       990
FCGR1A_NM_000566.3        (895) ---------------------------GCCTCCAGTTACCA----
FCGR1A_XM_005244957.3     (898) ---------------------------GCCTCCAGTTACCA----
FCGR1A_XM_005244958.4     (636) ---------------------------GCCTCCAGTTACCA----
FCGR1B_NM_001244910.1     (934) GAC------------------------TCCCTTATCTCCCATGGG
FCGR1B_NM_001017986.3     (613) ---------------------------GCCTCCAGTTACCA----
FCGR1B_NM_001004340.3     (337) ---------------------------GCCTCCAGTTACCA----
FCGR1B_XM_017000662.1     (358) ---------------------------GCCTCCAGTTACCA----
FCGR1B_XM_017000661.1     (616) ---------------------------GCCTCCAGTTACCA----
FCGR1B_XR_001737041.1     (937) GACTCCCTTATCTCCCATGGGACTGAGGCCTCCAGTTACCA----
FCGR1B_XR_001737040.1     (901) ---------------------------GCCTCCAGTTACCA----
FCGR1B_NR_045213.1        (898) ---------------------------GCCTCCAGTTACCA----
FCGR1CP_NR_027484.2       (897) ---------------------------GCCTCCAGTTACCA----
Consensus                 (946)                            GCCTCCAGTTACCA
                                                                              Section 23
                          (991) 991      1000      1010      1020           1035
FCGR1A_NM_000566.3        (909) ACTCCTGTCTGGTTTCATGTCCTTTTCTATCTGGCA-GTGGGAAT
FCGR1A_XM_005244957.3     (912) ACTCCTGTCTGGTTTCATGTCCTTTTCTATCTGGCA-GTGGGAAT
FCGR1A_XM_005244958.4     (650) ACTCCTGTCTGGTTTCATGTCCTTTTCTATCTGGCA-GTGGGAAT
FCGR1B_NM_001244910.1     (955) ACTGAGGTTTG--TTCAAGGGTTTTTGGCCCAGACAAGAGGGGAA
FCGR1B_NM_001017986.3     (627) ACTCCTGTCTGGTTTCATGTCCTTTTCTATCTGGCA-GTGGGAAT
FCGR1B_NM_001004340.3     (351) ACTCCTGTCTGGTTTCATGTCCTTTTCTATCTGGCA-GTGGGAAT
FCGR1B_XM_017000662.1     (372) ACTCCTGTCTGGTTTCATGTCCTTTTCTATCTGGCA-GTGGGAAT
FCGR1B_XM_017000661.1     (630) ACTCCTGTCTGGTTTCATGTCCTTTTCTATCTGGCA-GTGGGAAT
FCGR1B_XR_001737041.1     (978) ACTCCTGTCTGGTTTCATGTCCTTTTCTATCTGGCA-GTGGGAAT
FCGR1B_XR_001737040.1     (915) ACTCCTGTCTGGTTTCATGTCCTTTTCTATCTGGCA-GTGGGAAT
FCGR1B_NR_045213.1        (912) ACTCCTGTCTGGTTTCATGTCCTTTTCTATCTGGCA-GTGGGAAT
FCGR1CP_NR_027484.2       (911) ACTCCTGTCTGGTTTCATGTCCTTTTCTATCTGGCA-GTGGGAAT
Consensus                 (991) ACTCCTGTCTGGTTTCATGTCCTTTTCTATCTGGCA GTGGGAAT
                                                                              Section 24
                         (1036) 1036          1050      1060      1070      1080
FCGR1A_NM_000566.3        (953) AATGTTTTTAGTGAACACTGTTCTCTGGG--TGACAATACGTAAA
FCGR1A_XM_005244957.3     (956) AATGTTTTTAGTGAACACTGTTCTCTGGG--TGACAATACGTAAA
FCGR1A_XM_005244958.4     (694) AATGTTTTTAGTGAACACTGTTCTCTGGG--TGACAATACGTAAA
FCGR1B_NM_001244910.1     (998) AGTCTCTTCAGGAAAAGCCCACAAGCAGGCCTTTCCATCCTTGAT
FCGR1B_NM_001017986.3     (671) AATGTTTTTAGTGAACACTGTTCTCTGGG--TGACAATACGTAAA
FCGR1B_NM_001004340.3     (395) AATGTTTTTAGTGAACACTGTTCTCTGGG--TGACAATACGTAAA
FCGR1B_XM_017000662.1     (416) AATGTTTTTAGTGAACACTGTTCTCTGGG--TGACAATACGTAAA
FCGR1B_XM_017000661.1     (674) AATGTTTTTAGTGAACACTGTTCTCTGGG--TGACAATACGTAAA
FCGR1B_XR_001737041.1    (1022) AATGTTTTTAGTGAACACTGTTCTCTGGG--TGACAATACGTAAA
FCGR1B_XR_001737040.1     (959) AATGTTTTTAGTGAACACTGTTCTCTGGG--TGACAATACGTAAA
FCGR1B_NR_045213.1        (956) AATGTTTTTAGTGAACACTGTTCTCTGGG--TGACAATACGTAAA
FCGR1CP_NR_027484.2       (955) AATGTTTTTAGTGAACACTGTTCTCTGGG--TGACAATACGTAAA
Consensus                (1036) AATGTTTTTAGTGAACACTGTTCTCTGGG  TGACAATACGTAAA
```

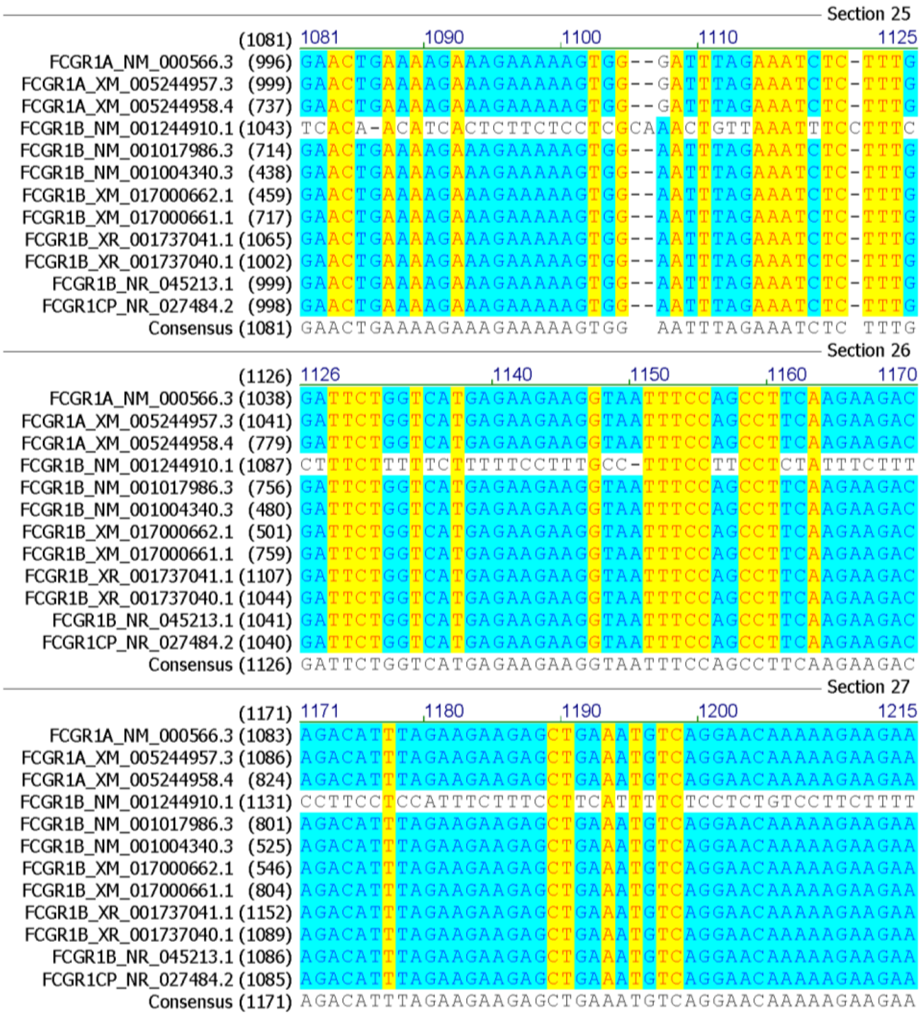
Section 25
(1081) 1081 1090 1100 1110 1125
FCGR1A_NM_000566.3 (996) GAACTGAAAAGAAAGAAAAAGTGG--GATTTAGAAATCTC-TTTG
FCGR1A_XM_005244957.3 (999) GAACTGAAAAGAAAGAAAAAGTGG--GATTTAGAAATCTC-TTTG
FCGR1A_XM_005244958.4 (737) GAACTGAAAAGAAAGAAAAAGTGG--GATTTAGAAATCTC-TTTG
FCGR1B_NM_001244910.1 (1043) TCACA-ACATCACTCTTCTCCTCGCAAACTGTTAAATTTCCTTTC
FCGR1B_NM_001017986.3 (714) GAACTGAAAAGAAAGAAAAAGTGG--AATTTAGAAATCTC-TTTG
FCGR1B_NM_001004340.3 (438) GAACTGAAAAGAAAGAAAAAGTGG--AATTTAGAAATCTC-TTTG
FCGR1B_XM_017000662.1 (459) GAACTGAAAAGAAAGAAAAAGTGG--AATTTAGAAATCTC-TTTG
FCGR1B_XM_017000661.1 (717) GAACTGAAAAGAAAGAAAAAGTGG--AATTTAGAAATCTC-TTTG
FCGR1B_XR_001737041.1 (1065) GAACTGAAAAGAAAGAAAAAGTGG--AATTTAGAAATCTC-TTTG
FCGR1B_XR_001737040.1 (1002) GAACTGAAAAGAAAGAAAAAGTGG--AATTTAGAAATCTC-TTTG
FCGR1B_NR_045213.1 (999) GAACTGAAAAGAAAGAAAAAGTGG--AATTTAGAAATCTC-TTTG
FCGR1CP_NR_027484.2 (998) GAACTGAAAAGAAAGAAAAAGTGG--AATTTAGAAATCTC-TTTG
Consensus (1081) GAACTGAAAAGAAAGAAAAAGTGG AATTTAGAAATCTC TTTG
Section 26
(1126) 1126 1140 1150 1160 1170
FCGR1A_NM_000566.3 (1038) GATTCTGGTCATGAGAAGAAGGTAATTTCCAGCCTTCAAGAAGAC
FCGR1A_XM_005244957.3 (1041) GATTCTGGTCATGAGAAGAAGGTAATTTCCAGCCTTCAAGAAGAC
FCGR1A_XM_005244958.4 (779) GATTCTGGTCATGAGAAGAAGGTAATTTCCAGCCTTCAAGAAGAC
FCGR1B_NM_001244910.1 (1087) CTTTCTTTTTCTTTTTCCTTTGCC-TTTCCTTCCTCTATTTCTTT
FCGR1B_NM_001017986.3 (756) GATTCTGGTCATGAGAAGAAGGTAATTTCCAGCCTTCAAGAAGAC
FCGR1B_NM_001004340.3 (480) GATTCTGGTCATGAGAAGAAGGTAATTTCCAGCCTTCAAGAAGAC
FCGR1B_XM_017000662.1 (501) GATTCTGGTCATGAGAAGAAGGTAATTTCCAGCCTTCAAGAAGAC
FCGR1B_XM_017000661.1 (759) GATTCTGGTCATGAGAAGAAGGTAATTTCCAGCCTTCAAGAAGAC
FCGR1B_XR_001737041.1 (1107) GATTCTGGTCATGAGAAGAAGGTAATTTCCAGCCTTCAAGAAGAC
FCGR1B_XR_001737040.1 (1044) GATTCTGGTCATGAGAAGAAGGTAATTTCCAGCCTTCAAGAAGAC
FCGR1B_NR_045213.1 (1041) GATTCTGGTCATGAGAAGAAGGTAATTTCCAGCCTTCAAGAAGAC
FCGR1CP_NR_027484.2 (1040) GATTCTGGTCATGAGAAGAAGGTAATTTCCAGCCTTCAAGAAGAC
Consensus (1126) GATTCTGGTCATGAGAAGAAGGTAATTTCCAGCCTTCAAGAAGAC
Section 27
(1171) 1171 1180 1190 1200 1215
FCGR1A_NM_000566.3 (1083) AGACATTTAGAAGAAGAGCTGAAATGTCAGGAACAAAAAGAAGAA
FCGR1A_XM_005244957.3 (1086) AGACATTTAGAAGAAGAGCTGAAATGTCAGGAACAAAAAGAAGAA
FCGR1A_XM_005244958.4 (824) AGACATTTAGAAGAAGAGCTGAAATGTCAGGAACAAAAAGAAGAA
FCGR1B_NM_001244910.1 (1131) CCTTCCTCCATTTCTTTCCTTCATTTTCTCCTCTGTCCTTCTTTT
FCGR1B_NM_001017986.3 (801) AGACATTTAGAAGAAGAGCTGAAATGTCAGGAACAAAAAGAAGAA
FCGR1B_NM_001004340.3 (525) AGACATTTAGAAGAAGAGCTGAAATGTCAGGAACAAAAAGAAGAA
FCGR1B_XM_017000662.1 (546) AGACATTTAGAAGAAGAGCTGAAATGTCAGGAACAAAAAGAAGAA
FCGR1B_XM_017000661.1 (804) AGACATTTAGAAGAAGAGCTGAAATGTCAGGAACAAAAAGAAGAA
FCGR1B_XR_001737041.1 (1152) AGACATTTAGAAGAAGAGCTGAAATGTCAGGAACAAAAAGAAGAA
FCGR1B_XR_001737040.1 (1089) AGACATTTAGAAGAAGAGCTGAAATGTCAGGAACAAAAAGAAGAA
FCGR1B_NR_045213.1 (1086) AGACATTTAGAAGAAGAGCTGAAATGTCAGGAACAAAAAGAAGAA
FCGR1CP_NR_027484.2 (1085) AGACATTTAGAAGAAGAGCTGAAATGTCAGGAACAAAAAGAAGAA
Consensus (1171) AGACATTTAGAAGAAGAGCTGAAATGTCAGGAACAAAAAGAAGAA

```
                                                                                Section 28
                           (1216) 1216          1230      1240      1250          1260
      FCGR1A_NM_000566.3 (1128) CAGCTGCAGGAAGGGGTGCACCGGAAGGAGCCCCAGGGGGCCACG
   FCGR1A_XM_005244957.3 (1131) CAGCTGCAGGAAGGGGTGCACCGGAAGGAGCCCCAGGGGGCCACG
   FCGR1A_XM_005244958.4  (869) CAGCTGCAGGAAGGGGTGCACCGGAAGGAGCCCCAGGGGGCCACG
   FCGR1B_NM_001244910.1 (1176) CTTCTCCTTCATTTTATTTTCCCCTCCCTCCCACTCTTCCCTCCA
   FCGR1B_NM_001017986.3  (846) CAGCTGCAGGAAGGGGTGCACCGGAAGGAGCCCCAGGGGGCCACG
   FCGR1B_NM_001004340.3  (570) CAGCTGCAGGAAGGGGTGCACCGGAAGGAGCCCCAGGGGGCCACG
   FCGR1B_XM_017000662.1  (591) CAGCTGCAGGAAGGGGTGCACCGGAAGGAGCCCCAGGGGGCCACG
   FCGR1B_XM_017000661.1  (849) CAGCTGCAGGAAGGGGTGCACCGGAAGGAGCCCCAGGGGGCCACG
   FCGR1B_XR_001737041.1 (1197) CAGCTGCAGGAAGGGGTGCACCGGAAGGAGCCCCAGGGGGCCACG
   FCGR1B_XR_001737040.1 (1134) CAGCTGCAGGAAGGGGTGCACCGGAAGGAGCCCCAGGGGGCCACG
      FCGR1B_NR_045213.1 (1131) CAGCTGCAGGAAGGGGTGCACCGGAAGGAGCCCCAGGGGGCCACG
     FCGR1CP_NR_027484.2 (1130) CAGCTGCAGGAAGGGGTGCACCGGAAGGAGCCCCAGGGGGCCACG
               Consensus (1216) CAGCTGCAGGAAGGGGTGCACCGGAAGGAGCCCCAGGGGGCCACG
                                                                                Section 29
                           (1261) 1261     1270      1280      1290            1305
      FCGR1A_NM_000566.3 (1173) TAGCAG--CGGCTCAGTGGGTGGCCATCGATCTGGACCGTCCCCT
   FCGR1A_XM_005244957.3 (1176) TAGCAG--CGGCTCAGTGGGTGGCCATCGATCTGGACCGTCCCCT
   FCGR1A_XM_005244958.4  (914) TAGCAG--CGGCTCAGTGGGTGGCCATCGATCTGGACCGTCCCCT
   FCGR1B_NM_001244910.1 (1221) CTCCATGACCCCTCCTTCTCTCTCTCTCTCTCTCCCTGCTTCCCT
   FCGR1B_NM_001017986.3  (891) TAGCAG--CGGCTCAGTTGGTGGCCATCGATCTGGACCGTCCCCT
   FCGR1B_NM_001004340.3  (615) TAGCAG--CGGCTCAGTTGGTGGCCATCGATCTGGACCGTCCCCT
   FCGR1B_XM_017000662.1  (636) TAGCAG--CGGCTCAGTTGGTGGCCATCGATCTGGACCGTCCCCT
   FCGR1B_XM_017000661.1  (894) TAGCAG--CGGCTCAGTTGGTGGCCATCGATCTGGACCGTCCCCT
   FCGR1B_XR_001737041.1 (1242) TAGCAG--CGGCTCAGTTGGTGGCCATCGATCTGGACCGTCCCCT
   FCGR1B_XR_001737040.1 (1179) TAGCAG--CGGCTCAGTTGGTGGCCATCGATCTGGACCGTCCCCT
      FCGR1B_NR_045213.1 (1176) TAGCAG--CGGCTCAGTTGGTGGCCATCGATCTGGACCGTCCCCT
     FCGR1CP_NR_027484.2 (1175) TAGCAG--CGGCTCAGTGGGTGGCCATCGATCTGGACCGTCCCCT
               Consensus (1261) TAGCAG  CGGCTCAGTTGGTGGCCATCGATCTGGACCGTCCCCT
                                                                                Section 30
                           (1306) 1306          1320      1330      1340          1350
      FCGR1A_NM_000566.3 (1216) GCCCACTTGCTCCCCGTGAGCACTGCGTACAAACATCCAA-AAGT
   FCGR1A_XM_005244957.3 (1219) GCCCACTTGCTCCCCGTGAGCACTGCGTACAAACATCCAA-AAGT
   FCGR1A_XM_005244958.4  (957) GCCCACTTGCTCCCCGTGAGCACTGCGTACAAACATCCAA-AAGT
   FCGR1B_NM_001244910.1 (1266) GCCTCCCTCCTCCTCACCAACAATCTCACCAACAATTTATCAAGT
   FCGR1B_NM_001017986.3  (934) GCCCACTTGCTCCCCGTGAGCACTGCGTACAAACATCCAA-AAGT
   FCGR1B_NM_001004340.3  (658) GCCCACTTGCTCCCCGTGAGCACTGCGTACAAACATCCAA-AAGT
   FCGR1B_XM_017000662.1  (679) GCCCACTTGCTCCCCGTGAGCACTGCGTACAAACATCCAA-AAGT
   FCGR1B_XM_017000661.1  (937) GCCCACTTGCTCCCCGTGAGCACTGCGTACAAACATCCAA-AAGT
   FCGR1B_XR_001737041.1 (1285) GCCCACTTGCTCCCCGTGAGCACTGCGTACAAACATCCAA-AAGT
   FCGR1B_XR_001737040.1 (1222) GCCCACTTGCTCCCCGTGAGCACTGCGTACAAACATCCAA-AAGT
      FCGR1B_NR_045213.1 (1219) GCCCACTTGCTCCCCGTGAGCACTGCGTACAAACATCCAA-AAGT
     FCGR1CP_NR_027484.2 (1218) GTCCACTTGCTCCCCGTGAGCACTGCGTACAAACATCCAA-AAGT
               Consensus (1306) GCCCACTTGCTCCCCGTGAGCACTGCGTACAAACATCCAA AAGT
```

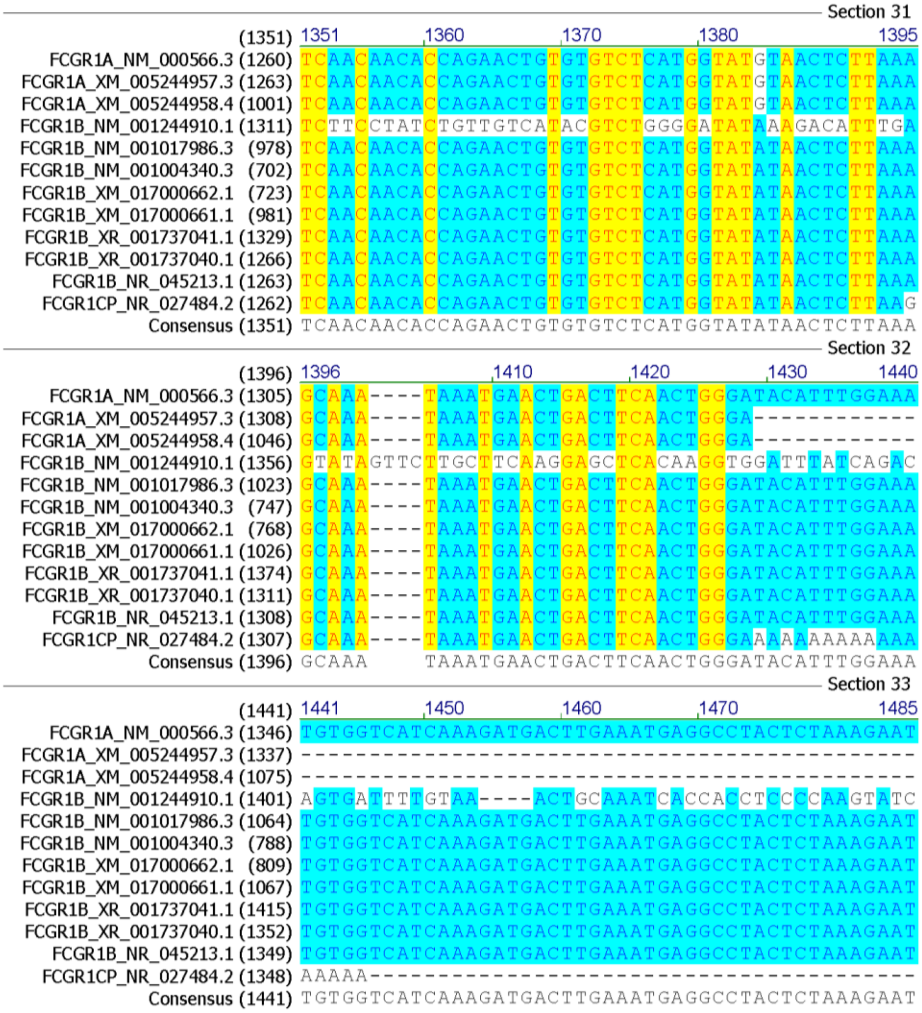
Section 31
(1351) 1351 1360 1370 1380 1395
FCGR1A_NM_000566.3 (1260) TCAACAACACCAGAACTGTGTGTCTCATGGTATGTAACTCTTAAA
FCGR1A_XM_005244957.3 (1263) TCAACAACACCAGAACTGTGTGTCTCATGGTATGTAACTCTTAAA
FCGR1A_XM_005244958.4 (1001) TCAACAACACCAGAACTGTGTGTCTCATGGTATGTAACTCTTAAA
FCGR1B_NM_001244910.1 (1311) TCTTCCTATCTGTTGTCATACGTCTGGGGATATAAAGACATTTGA
FCGR1B_NM_001017986.3 (978) TCAACAACACCAGAACTGTGTGTCTCATGGTATATAACTCTTAAA
FCGR1B_NM_001004340.3 (702) TCAACAACACCAGAACTGTGTGTCTCATGGTATATAACTCTTAAA
FCGR1B_XM_017000662.1 (723) TCAACAACACCAGAACTGTGTGTCTCATGGTATATAACTCTTAAA
FCGR1B_XM_017000661.1 (981) TCAACAACACCAGAACTGTGTGTCTCATGGTATATAACTCTTAAA
FCGR1B_XR_001737041.1 (1329) TCAACAACACCAGAACTGTGTGTCTCATGGTATATAACTCTTAAA
FCGR1B_XR_001737040.1 (1266) TCAACAACACCAGAACTGTGTGTCTCATGGTATATAACTCTTAAA
FCGR1B_NR_045213.1 (1263) TCAACAACACCAGAACTGTGTGTCTCATGGTATATAACTCTTAAA
FCGR1CP_NR_027484.2 (1262) TCAACAACACCAGAACTGTGTGTCTCATGGTATATAACTCTTAAG
Consensus (1351) TCAACAACACCAGAACTGTGTGTCTCATGGTATATAACTCTTAAA
Section 32
(1396) 1396 1410 1420 1430 1440
FCGR1A_NM_000566.3 (1305) GCAAA----TAAATGAACTGACTTCAACTGGGATACATTTGGAAA
FCGR1A_XM_005244957.3 (1308) GCAAA----TAAATGAACTGACTTCAACTGGGA------------
FCGR1A_XM_005244958.4 (1046) GCAAA----TAAATGAACTGACTTCAACTGGGA------------
FCGR1B_NM_001244910.1 (1356) GTATAGTTCTTGCTTCAAGGAGCTCACAAGGTGGATTTATCAGAC
FCGR1B_NM_001017986.3 (1023) GCAAA----TAAATGAACTGACTTCAACTGGGATACATTTGGAAA
FCGR1B_NM_001004340.3 (747) GCAAA----TAAATGAACTGACTTCAACTGGGATACATTTGGAAA
FCGR1B_XM_017000662.1 (768) GCAAA----TAAATGAACTGACTTCAACTGGGATACATTTGGAAA
FCGR1B_XM_017000661.1 (1026) GCAAA----TAAATGAACTGACTTCAACTGGGATACATTTGGAAA
FCGR1B_XR_001737041.1 (1374) GCAAA----TAAATGAACTGACTTCAACTGGGATACATTTGGAAA
FCGR1B_XR_001737040.1 (1311) GCAAA----TAAATGAACTGACTTCAACTGGGATACATTTGGAAA
FCGR1B_NR_045213.1 (1308) GCAAA----TAAATGAACTGACTTCAACTGGGATACATTTGGAAA
FCGR1CP_NR_027484.2 (1307) GCAAA----TAAATGAACTGACTTCAACTGGGAAAAAAAAAAAAA
Consensus (1396) GCAAA TAAATGAACTGACTTCAACTGGGATACATTTGGAAA
Section 33
(1441) 1441 1450 1460 1470 1485
FCGR1A_NM_000566.3 (1346) TGTGGTCATCAAAGATGACTTGAAATGAGGCCTACTCTAAAGAAT
FCGR1A_XM_005244957.3 (1337) ---
FCGR1A_XM_005244958.4 (1075) ---
FCGR1B_NM_001244910.1 (1401) AGTGATTTTGTAA----ACTGCAAATCACCACCTCCCCAAGTATC
FCGR1B_NM_001017986.3 (1064) TGTGGTCATCAAAGATGACTTGAAATGAGGCCTACTCTAAAGAAT
FCGR1B_NM_001004340.3 (788) TGTGGTCATCAAAGATGACTTGAAATGAGGCCTACTCTAAAGAAT
FCGR1B_XM_017000662.1 (809) TGTGGTCATCAAAGATGACTTGAAATGAGGCCTACTCTAAAGAAT
FCGR1B_XM_017000661.1 (1067) TGTGGTCATCAAAGATGACTTGAAATGAGGCCTACTCTAAAGAAT
FCGR1B_XR_001737041.1 (1415) TGTGGTCATCAAAGATGACTTGAAATGAGGCCTACTCTAAAGAAT
FCGR1B_XR_001737040.1 (1352) TGTGGTCATCAAAGATGACTTGAAATGAGGCCTACTCTAAAGAAT
FCGR1B_NR_045213.1 (1349) TGTGGTCATCAAAGATGACTTGAAATGAGGCCTACTCTAAAGAAT
FCGR1CP_NR_027484.2 (1348) AAAAA--
Consensus (1441) TGTGGTCATCAAAGATGACTTGAAATGAGGCCTACTCTAAAGAAT

```
                                                                              Section 34
                         (1486) 1486          1500      1510      1520          1530
    FCGR1A_NM_000566.3 (1391) TCT--TGAAAAACTTACAAGTCAAGCCTAGCCTGATAATCCTAT-
 FCGR1A_XM_005244957.3 (1337) ---------------------------------------------
 FCGR1A_XM_005244958.4 (1075) ---------------------------------------------
 FCGR1B_NM_001244910.1 (1442) TCTGTTTAACTGAGGCAGAGGGATTGTGAGCTCTAGAACAAAGTC
 FCGR1B_NM_001017986.3 (1109) TCT--TGAAAAACTTACAAGTCAAGCCTAGCCTGATAATCCTAT-
 FCGR1B_NM_001004340.3  (833) TCT--TGAAAAACTTACAAGTCAAGCCTAGCCTGATAATCCTAT-
 FCGR1B_XM_017000662.1  (854) TCT--TGAAAAACTTACAAGTCAAGCCTAGCCTGATAATCCTAT-
 FCGR1B_XM_017000661.1 (1112) TCT--TGAAAAACTTACAAGTCAAGCCTAGCCTGATAATCCTAT-
 FCGR1B_XR_001737041.1 (1460) TCT--TGAAAAACTTACAAGTCAAGCCTAGCCTGATAATCCTAT-
 FCGR1B_XR_001737040.1 (1397) TCT--TGAAAAACTTACAAGTCAAGCCTAGCCTGATAATCCTAT-
    FCGR1B_NR_045213.1 (1394) TCT--TGAAAAACTTACAAGTCAAGCCTAGCCTGATAATCCTAT-
   FCGR1CP_NR_027484.2 (1353) ---------------------------------------------
             Consensus (1486) TCT  TGAAAAACTTACAAGTCAAGCCTAGCCTGATAATCCTAT
                                                                              Section 35
                         (1531) 1531      1540      1550      1560          1575
    FCGR1A_NM_000566.3 (1433) TACATAGTTTGAAAAATAGTATTTTATTTCTCAGAACAAGGTAAA
 FCGR1A_XM_005244957.3 (1337) ---------------------------------------------
 FCGR1A_XM_005244958.4 (1075) ---------------------------------------------
 FCGR1B_NM_001244910.1 (1487) TCCTTGGGGGGGAAAAAAGTTCATCTTCAACCCAAATTCATTTCA
 FCGR1B_NM_001017986.3 (1151) TACATAGTTTGAAAAATAGTATTTTATTTCTCAGAACAAGGTAAA
 FCGR1B_NM_001004340.3  (875) TACATAGTTTGAAAAATAGTATTTTATTTCTCAGAACAAGGTAAA
 FCGR1B_XM_017000662.1  (896) TACATAGTTTGAAAAATAGTATTTTATTTCTCAGAACAAGGTAAA
 FCGR1B_XM_017000661.1 (1154) TACATAGTTTGAAAAATAGTATTTTATTTCTCAGAACAAGGTAAA
 FCGR1B_XR_001737041.1 (1502) TACATAGTTTGAAAAATAGTATTTTATTTCTCAGAACAAGGTAAA
 FCGR1B_XR_001737040.1 (1439) TACATAGTTTGAAAAATAGTATTTTATTTCTCAGAACAAGGTAAA
    FCGR1B_NR_045213.1 (1436) TACATAGTTTGAAAAATAGTATTTTATTTCTCAGAACAAGGTAAA
   FCGR1CP_NR_027484.2 (1353) ---------------------------------------------
             Consensus (1531) TACATAGTTTGAAAAATAGTATTTTATTTCTCAGAACAAGGTAAA
                                                                              Section 36
                         (1576) 1576          1590      1600      1610          1620
    FCGR1A_NM_000566.3 (1478) AAGGTGAGTGGGTGCATATGTA-CAGAAGATTAAGACAGAGAAAC
 FCGR1A_XM_005244957.3 (1337) ---------------------------------------------
 FCGR1A_XM_005244958.4 (1075) ---------------------------------------------
 FCGR1B_NM_001244910.1 (1532) AGTATTAAATGGCACAGAGATATCAGTTGTCTCTG-----GAACT
 FCGR1B_NM_001017986.3 (1196) AAGGTGAGTGGGTGCATATGTA-CAGAAGATTAAGACAGAGAAAC
 FCGR1B_NM_001004340.3  (920) AAGGTGAGTGGGTGCATATGTA-CAGAAGATTAAGACAGAGAAAC
 FCGR1B_XM_017000662.1  (941) AAGGTGAGTGGGTGCATATGTA-CAGAAGATTAAGACAGAGAAAC
 FCGR1B_XM_017000661.1 (1199) AAGGTGAGTGGGTGCATATGTA-CAGAAGATTAAGACAGAGAAAC
 FCGR1B_XR_001737041.1 (1547) AAGGTGAGTGGGTGCATATGTA-CAGAAGATTAAGACAGAGAAAC
 FCGR1B_XR_001737040.1 (1484) AAGGTGAGTGGGTGCATATGTA-CAGAAGATTAAGACAGAGAAAC
    FCGR1B_NR_045213.1 (1481) AAGGTGAGTGGGTGCATATGTA-CAGAAGATTAAGACAGAGAAAC
   FCGR1CP_NR_027484.2 (1353) ---------------------------------------------
             Consensus (1576) AAGGTGAGTGGGTGCATATGTA CAGAAGATTAAGACAGAGAAAC
```

```
                                                               Section 37
                         (1621) 1621        1630       1640       1650       1665
       FCGR1A_NM_000566.3 (1522) AGACAGAAAGAGACACACACACAGCCAGGAGTGGGTAGATTTCAG
    FCGR1A_XM_005244957.3 (1337) ---------------------------------------------
    FCGR1A_XM_005244958.4 (1075) ---------------------------------------------
    FCGR1B_NM_001244910.1 (1572) AGGGAGTAAG-----------------------------------
    FCGR1B_NM_001017986.3 (1240) AGACAGAAAGAGACACACACACAGCCAGGAGTGGGTAGATTTCAG
    FCGR1B_NM_001004340.3  (964) AGACAGAAAGAGACACACACACAGCCAGGAGTGGGTAGATTTCAG
    FCGR1B_XM_017000662.1  (985) AGACAGAAAGAGACACACACACAGCCAGGAGTGGGTAGATTTCAG
    FCGR1B_XM_017000661.1 (1243) AGACAGAAAGAGACACACACACAGCCAGGAGTGGGTAGATTTCAG
    FCGR1B_XR_001737041.1 (1591) AGACAGAAAGAGACACACACACAGCCAGGAGTGGGTAGATTTCAG
    FCGR1B_XR_001737040.1 (1528) AGACAGAAAGAGACACACACACAGCCAGGAGTGGGTAGATTTCAG
       FCGR1B_NR_045213.1 (1525) AGACAGAAAGAGACACACACACAGCCAGGAGTGGGTAGATTTCAG
      FCGR1CP_NR_027484.2 (1353) ---------------------------------------------
                Consensus (1621) AGACAGAAAGAGACACACACACAGCCAGGAGTGGGTAGATTTCAG
                                                               Section 38
                         (1666) 1666              1680       1690       1700       1710
       FCGR1A_NM_000566.3 (1567) GGAGACAAGAGGGAATAGTATAGACAATAAGGAAGGAAATAGTAC
    FCGR1A_XM_005244957.3 (1337) ---------------------------------------------
    FCGR1A_XM_005244958.4 (1075) ---------------------------------------------
    FCGR1B_NM_001244910.1 (1582) ---------------------------------------------
    FCGR1B_NM_001017986.3 (1285) GGAGACAAGAGGGAATAGTATAGACAATAAGGAAGGAAATAGTAC
    FCGR1B_NM_001004340.3 (1009) GGAGACAAGAGGGAATAGTATAGACAATAAGGAAGGAAATAGTAC
    FCGR1B_XM_017000662.1 (1030) GGAGACAAGAGGGAATAGTATAGACAATAAGGAAGGAAATAGTAC
    FCGR1B_XM_017000661.1 (1288) GGAGACAAGAGGGAATAGTATAGACAATAAGGAAGGAAATAGTAC
    FCGR1B_XR_001737041.1 (1636) GGAGACAAGAGGGAATAGTATAGACAATAAGGAAGGAAATAGTAC
    FCGR1B_XR_001737040.1 (1573) GGAGACAAGAGGGAATAGTATAGACAATAAGGAAGGAAATAGTAC
       FCGR1B_NR_045213.1 (1570) GGAGACAAGAGGGAATAGTATAGACAATAAGGAAGGAAATAGTAC
      FCGR1CP_NR_027484.2 (1353) ---------------------------------------------
                Consensus (1666) GGAGACAAGAGGGAATAGTATAGACAATAAGGAAGGAAATAGTAC
                                                               Section 39
                         (1711) 1711        1720       1730       1740       1755
       FCGR1A_NM_000566.3 (1612) TTACAAATGACTCCTAAGGGACTGTGAGACTGAGAGGGCTCACGC
    FCGR1A_XM_005244957.3 (1337) ---------------------------------------------
    FCGR1A_XM_005244958.4 (1075) ---------------------------------------------
    FCGR1B_NM_001244910.1 (1582) ---------------------------------------------
    FCGR1B_NM_001017986.3 (1330) TTACAAATGACTCCTAAGGGACTGTGAGACTGAGAGGGCTCACGC
    FCGR1B_NM_001004340.3 (1054) TTACAAATGACTCCTAAGGGACTGTGAGACTGAGAGGGCTCACGC
    FCGR1B_XM_017000662.1 (1075) TTACAAATGACTCCTAAGGGACTGTGAGACTGAGAGGGCTCACGC
    FCGR1B_XM_017000661.1 (1333) TTACAAATGACTCCTAAGGGACTGTGAGACTGAGAGGGCTCACGC
    FCGR1B_XR_001737041.1 (1681) TTACAAATGACTCCTAAGGGACTGTGAGACTGAGAGGGCTCACGC
    FCGR1B_XR_001737040.1 (1618) TTACAAATGACTCCTAAGGGACTGTGAGACTGAGAGGGCTCACGC
       FCGR1B_NR_045213.1 (1615) TTACAAATGACTCCTAAGGGACTGTGAGACTGAGAGGGCTCACGC
      FCGR1CP_NR_027484.2 (1353) ---------------------------------------------
                Consensus (1711) TTACAAATGACTCCTAAGGGACTGTGAGACTGAGAGGGCTCACGC
```

```
                                                                      Section 40
                         (1756) 1756          1770      1780      1790       1800
     FCGR1A_NM_000566.3 (1657) CTCTGTGTTCAGGATACTTAGTTCATGGCTTTTCTCTTTGACTTT
   FCGR1A_XM_005244957.3 (1337) ---------------------------------------------
   FCGR1A_XM_005244958.4 (1075) ---------------------------------------------
   FCGR1B_NM_001244910.1 (1582) ---------------------------------------------
   FCGR1B_NM_001017986.3 (1375) CTCTGTGTTCAGGATACTTAGTTCATGGCTTTTCTCTTTGACTTT
   FCGR1B_NM_001004340.3 (1099) CTCTGTGTTCAGGATACTTAGTTCATGGCTTTTCTCTTTGACTTT
   FCGR1B_XM_017000662.1 (1120) CTCTGTGTTCAGGATACTTAGTTCATGGCTTTTCTCTTTGACTTT
   FCGR1B_XM_017000661.1 (1378) CTCTGTGTTCAGGATACTTAGTTCATGGCTTTTCTCTTTGACTTT
   FCGR1B_XR_001737041.1 (1726) CTCTGTGTTCAGGATACTTAGTTCATGGCTTTTCTCTTTGACTTT
   FCGR1B_XR_001737040.1 (1663) CTCTGTGTTCAGGATACTTAGTTCATGGCTTTTCTCTTTGACTTT
      FCGR1B_NR_045213.1 (1660) CTCTGTGTTCAGGATACTTAGTTCATGGCTTTTCTCTTTGACTTT
     FCGR1CP_NR_027484.2 (1353) ---------------------------------------------
               Consensus (1756) CTCTGTGTTCAGGATACTTAGTTCATGGCTTTTCTCTTTGACTTT
                                                                      Section 41
                         (1801) 1801     1810      1820      1830           1845
     FCGR1A_NM_000566.3 (1702) ACTAAAAGAGAATGTCTCCATACGCGTTCTAGGCATACAAGGGGG
   FCGR1A_XM_005244957.3 (1337) ---------------------------------------------
   FCGR1A_XM_005244958.4 (1075) ---------------------------------------------
   FCGR1B_NM_001244910.1 (1582) ---------------------------------------------
   FCGR1B_NM_001017986.3 (1420) ACTAAAAGAGAATGTCTCCATACGCGTTCTAGGCATACAAGGGGG
   FCGR1B_NM_001004340.3 (1144) ACTAAAAGAGAATGTCTCCATACGCGTTCTAGGCATACAAGGGGG
   FCGR1B_XM_017000662.1 (1165) ACTAAAAGAGAATGTCTCCATACGCGTTCTAGGCATACAAGGGGG
   FCGR1B_XM_017000661.1 (1423) ACTAAAAGAGAATGTCTCCATACGCGTTCTAGGCATACAAGGGGG
   FCGR1B_XR_001737041.1 (1771) ACTAAAAGAGAATGTCTCCATACGCGTTCTAGGCATACAAGGGGG
   FCGR1B_XR_001737040.1 (1708) ACTAAAAGAGAATGTCTCCATACGCGTTCTAGGCATACAAGGGGG
      FCGR1B_NR_045213.1 (1705) ACTAAAAGAGAATGTCTCCATACGCGTTCTAGGCATACAAGGGGG
     FCGR1CP_NR_027484.2 (1353) ---------------------------------------------
               Consensus (1801) ACTAAAAGAGAATGTCTCCATACGCGTTCTAGGCATACAAGGGGG
                                                                      Section 42
                         (1846) 1846          1860      1870      1880       1890
     FCGR1A_NM_000566.3 (1747) TAACTCATGATGAGAAATGGATGTGTTATTCTTGCCCTCTCTTTT
   FCGR1A_XM_005244957.3 (1337) ---------------------------------------------
   FCGR1A_XM_005244958.4 (1075) ---------------------------------------------
   FCGR1B_NM_001244910.1 (1582) ---------------------------------------------
   FCGR1B_NM_001017986.3 (1465) TAACTCATGATGAGAAATGGATGTGTTATTCTTGCCCTCTCTTTT
   FCGR1B_NM_001004340.3 (1189) TAACTCATGATGAGAAATGGATGTGTTATTCTTGCCCTCTCTTTT
   FCGR1B_XM_017000662.1 (1210) TAACTCATGATGAGAAATGGATGTGTTATTCTTGCCCTCTCTTTT
   FCGR1B_XM_017000661.1 (1468) TAACTCATGATGAGAAATGGATGTGTTATTCTTGCCCTCTCTTTT
   FCGR1B_XR_001737041.1 (1816) TAACTCATGATGAGAAATGGATGTGTTATTCTTGCCCTCTCTTTT
   FCGR1B_XR_001737040.1 (1753) TAACTCATGATGAGAAATGGATGTGTTATTCTTGCCCTCTCTTTT
      FCGR1B_NR_045213.1 (1750) TAACTCATGATGAGAAATGGATGTGTTATTCTTGCCCTCTCTTTT
     FCGR1CP_NR_027484.2 (1353) ---------------------------------------------
               Consensus (1846) TAACTCATGATGAGAAATGGATGTGTTATTCTTGCCCTCTCTTTT
```

```
                                                                              Section 43
                          (1891) 1891         1900          1910          1920          1935
     FCGR1A_NM_000566.3 (1792) GAGGCTCTCTCATAACCCCTCTATTTCTAGAGACAACAAAAATGC
  FCGR1A_XM_005244957.3 (1337) ---------------------------------------------
  FCGR1A_XM_005244958.4 (1075) ---------------------------------------------
  FCGR1B_NM_001244910.1 (1582) ---------------------------------------------
  FCGR1B_NM_001017986.3 (1510) GAGGCTCTCTCATAACCCCTCTATTTCTAGAGACAACAAAAATGT
  FCGR1B_NM_001004340.3 (1234) GAGGCTCTCTCATAACCCCTCTATTTCTAGAGACAACAAAAATGT
  FCGR1B_XM_017000662.1 (1255) GAGGCTCTCTCATAACCCCTCTATTTCTAGAGACAACAAAAATGT
  FCGR1B_XM_017000661.1 (1513) GAGGCTCTCTCATAACCCCTCTATTTCTAGAGACAACAAAAATGT
  FCGR1B_XR_001737041.1 (1861) GAGGCTCTCTCATAACCCCTCTATTTCTAGAGACAACAAAAATGT
  FCGR1B_XR_001737040.1 (1798) GAGGCTCTCTCATAACCCCTCTATTTCTAGAGACAACAAAAATGT
     FCGR1B_NR_045213.1 (1795) GAGGCTCTCTCATAACCCCTCTATTTCTAGAGACAACAAAAATGT
    FCGR1CP_NR_027484.2 (1353) ---------------------------------------------
               Consensus (1891) GAGGCTCTCTCATAACCCCTCTATTTCTAGAGACAACAAAAATGT
                                                                              Section 44
                          (1936) 1936          1950          1960          1970          1980
     FCGR1A_NM_000566.3 (1837) TGCCAGTCCTAGGCCCCTGCCCTGTAGGAAGGCAGAATGTAACTG
  FCGR1A_XM_005244957.3 (1337) ---------------------------------------------
  FCGR1A_XM_005244958.4 (1075) ---------------------------------------------
  FCGR1B_NM_001244910.1 (1582) ---------------------------------------------
  FCGR1B_NM_001017986.3 (1555) TGCCAGTCCTAGGCCCCTGCCCTGTAGGAAGGCAGAATGTAACTG
  FCGR1B_NM_001004340.3 (1279) TGCCAGTCCTAGGCCCCTGCCCTGTAGGAAGGCAGAATGTAACTG
  FCGR1B_XM_017000662.1 (1300) TGCCAGTCCTAGGCCCCTGCCCTGTAGGAAGGCAGAATGTAACTG
  FCGR1B_XM_017000661.1 (1558) TGCCAGTCCTAGGCCCCTGCCCTGTAGGAAGGCAGAATGTAACTG
  FCGR1B_XR_001737041.1 (1906) TGCCAGTCCTAGGCCCCTGCCCTGTAGGAAGGCAGAATGTAACTG
  FCGR1B_XR_001737040.1 (1843) TGCCAGTCCTAGGCCCCTGCCCTGTAGGAAGGCAGAATGTAACTG
     FCGR1B_NR_045213.1 (1840) TGCCAGTCCTAGGCCCCTGCCCTGTAGGAAGGCAGAATGTAACTG
    FCGR1CP_NR_027484.2 (1353) ---------------------------------------------
               Consensus (1936) TGCCAGTCCTAGGCCCCTGCCCTGTAGGAAGGCAGAATGTAACTG
                                                                              Section 45
                          (1981) 1981         1990          2000          2010          2025
     FCGR1A_NM_000566.3 (1882) TTCTGTTTGTTTAACGATTAAGTCCAAATCTCCAAGTGCGGCACT
  FCGR1A_XM_005244957.3 (1337) ---------------------------------------------
  FCGR1A_XM_005244958.4 (1075) ---------------------------------------------
  FCGR1B_NM_001244910.1 (1582) ---------------------------------------------
  FCGR1B_NM_001017986.3 (1600) TTCTTTTTGTTTAACGATTAAGTCCAAATCTCCAAGTGCGGCACT
  FCGR1B_NM_001004340.3 (1324) TTCTTTTTGTTTAACGATTAAGTCCAAATCTCCAAGTGCGGCACT
  FCGR1B_XM_017000662.1 (1345) TTCTTTTTGTTTAACGATTAAGTCCAAATCTCCAAGTGCGGCACT
  FCGR1B_XM_017000661.1 (1603) TTCTTTTTGTTTAACGATTAAGTCCAAATCTCCAAGTGCGGCACT
  FCGR1B_XR_001737041.1 (1951) TTCTTTTTGTTTAACGATTAAGTCCAAATCTCCAAGTGCGGCACT
  FCGR1B_XR_001737040.1 (1888) TTCTTTTTGTTTAACGATTAAGTCCAAATCTCCAAGTGCGGCACT
     FCGR1B_NR_045213.1 (1885) TTCTTTTTGTTTAACGATTAAGTCCAAATCTCCAAGTGCGGCACT
    FCGR1CP_NR_027484.2 (1353) ---------------------------------------------
               Consensus (1981) TTCTTTTTGTTTAACGATTAAGTCCAAATCTCCAAGTGCGGCACT
```

Section 46

(2026)	2026 2040 2050 2060 2070
FCGR1A_NM_000566.3 (1927)	GCAAAGAGACGCTTCAAGTGGGGAGAAGCGGCGATACCATAGAGT
FCGR1A_XM_005244957.3 (1337)	---
FCGR1A_XM_005244958.4 (1075)	---
FCGR1B_NM_001244910.1 (1582)	---
FCGR1B_NM_001017986.3 (1645)	GCAAAGAGACGCTTCAAGTGGGGAGAAGCGGCGATATCATAGAGT
FCGR1B_NM_001004340.3 (1369)	GCAAAGAGACGCTTCAAGTGGGGAGAAGCGGCGATATCATAGAGT
FCGR1B_XM_017000662.1 (1390)	GCAAAGAGACGCTTCAAGTGGGGAGAAGCGGCGATATCATAGAGT
FCGR1B_XM_017000661.1 (1648)	GCAAAGAGACGCTTCAAGTGGGGAGAAGCGGCGATATCATAGAGT
FCGR1B_XR_001737041.1 (1996)	GCAAAGAGACGCTTCAAGTGGGGAGAAGCGGCGATATCATAGAGT
FCGR1B_XR_001737040.1 (1933)	GCAAAGAGACGCTTCAAGTGGGGAGAAGCGGCGATATCATAGAGT
FCGR1B_NR_045213.1 (1930)	GCAAAGAGACGCTTCAAGTGGGGAGAAGCGGCGATATCATAGAGT
FCGR1CP_NR_027484.2 (1353)	---
Consensus (2026)	GCAAAGAGACGCTTCAAGTGGGGAGAAGCGGCGATATCATAGAGT

Section 47

(2071)	2071 2080 2090 2100 2115
FCGR1A_NM_000566.3 (1972)	CCAGATCTTGCCTCCAGAGATTTGCTTTACCTTCCTGATTTTCTG
FCGR1A_XM_005244957.3 (1337)	---
FCGR1A_XM_005244958.4 (1075)	---
FCGR1B_NM_001244910.1 (1582)	---
FCGR1B_NM_001017986.3 (1690)	CCAGATCTTGCCTCCAGAGATTTGCTTTACCTTCCTGATTTTCTG
FCGR1B_NM_001004340.3 (1414)	CCAGATCTTGCCTCCAGAGATTTGCTTTACCTTCCTGATTTTCTG
FCGR1B_XM_017000662.1 (1435)	CCAGATCTTGCCTCCAGAGATTTGCTTTACCTTCCTGATTTTCTG
FCGR1B_XM_017000661.1 (1693)	CCAGATCTTGCCTCCAGAGATTTGCTTTACCTTCCTGATTTTCTG
FCGR1B_XR_001737041.1 (2041)	CCAGATCTTGCCTCCAGAGATTTGCTTTACCTTCCTGATTTTCTG
FCGR1B_XR_001737040.1 (1978)	CCAGATCTTGCCTCCAGAGATTTGCTTTACCTTCCTGATTTTCTG
FCGR1B_NR_045213.1 (1975)	CCAGATCTTGCCTCCAGAGATTTGCTTTACCTTCCTGATTTTCTG
FCGR1CP_NR_027484.2 (1353)	---
Consensus (2071)	CCAGATCTTGCCTCCAGAGATTTGCTTTACCTTCCTGATTTTCTG

Section 48

(2116)	2116 2130 2140 2150 2160
FCGR1A_NM_000566.3 (2017)	GTTACTAATTAGCTTCAGGATACGCTGCTCTCATACTTGGGCTGT
FCGR1A_XM_005244957.3 (1337)	---
FCGR1A_XM_005244958.4 (1075)	---
FCGR1B_NM_001244910.1 (1582)	---
FCGR1B_NM_001017986.3 (1735)	GTTACTAATTAGCTTCAGGATACGCTGCTCTCATACTTGGGCTGT
FCGR1B_NM_001004340.3 (1459)	GTTACTAATTAGCTTCAGGATACGCTGCTCTCATACTTGGGCTGT
FCGR1B_XM_017000662.1 (1480)	GTTACTAATTAGCTTCAGGATACGCTGCTCTCATACTTGGGCTGT
FCGR1B_XM_017000661.1 (1738)	GTTACTAATTAGCTTCAGGATACGCTGCTCTCATACTTGGGCTGT
FCGR1B_XR_001737041.1 (2086)	GTTACTAATTAGCTTCAGGATACGCTGCTCTCATACTTGGGCTGT
FCGR1B_XR_001737040.1 (2023)	GTTACTAATTAGCTTCAGGATACGCTGCTCTCATACTTGGGCTGT
FCGR1B_NR_045213.1 (2020)	GTTACTAATTAGCTTCAGGATACGCTGCTCTCATACTTGGGCTGT
FCGR1CP_NR_027484.2 (1353)	---
Consensus (2116)	GTTACTAATTAGCTTCAGGATACGCTGCTCTCATACTTGGGCTGT

```
                                                    Section 49
                     (2161) 2161     2170      2180      2190      2205
 FCGR1A_NM_000566.3 (2062) AGTTTGGAGACAAAATATTTTCCTGCCACTGTGTAACATAGCTGA
 FCGR1A_XM_005244957.3 (1337) ---------------------------------------------
 FCGR1A_XM_005244958.4 (1075) ---------------------------------------------
 FCGR1B_NM_001244910.1 (1582) ---------------------------------------------
 FCGR1B_NM_001017986.3 (1780) AGTTTGGAGACAAAATATTTTCCTGCCACTGTGTAACATAGCTGA
 FCGR1B_NM_001004340.3 (1504) AGTTTGGAGACAAAATATTTTCCTGCCACTGTGTAACATAGCTGA
 FCGR1B_XM_017000662.1 (1525) AGTTTGGAGACAAAATATTTTCCTGCCACTGTGTAACATAGCTGA
 FCGR1B_XM_017000661.1 (1783) AGTTTGGAGACAAAATATTTTCCTGCCACTGTGTAACATAGCTGA
 FCGR1B_XR_001737041.1 (2131) AGTTTGGAGACAAAATATTTTCCTGCCACTGTGTAACATAGCTGA
 FCGR1B_XR_001737040.1 (2068) AGTTTGGAGACAAAATATTTTCCTGCCACTGTGTAACATAGCTGA
 FCGR1B_NR_045213.1 (2065) AGTTTGGAGACAAAATATTTTCCTGCCACTGTGTAACATAGCTGA
 FCGR1CP_NR_027484.2 (1353) ---------------------------------------------
 Consensus (2161) AGTTTGGAGACAAAATATTTTCCTGCCACTGTGTAACATAGCTGA
                                                    Section 50
                     (2206) 2206           2220      2230      2240     2250
 FCGR1A_NM_000566.3 (2107) GGTAAAAACTGAACTATGTAAATGACTCTACTAAAAGTTTAGGGA
 FCGR1A_XM_005244957.3 (1337) ---------------------------------------------
 FCGR1A_XM_005244958.4 (1075) ---------------------------------------------
 FCGR1B_NM_001244910.1 (1582) ---------------------------------------------
 FCGR1B_NM_001017986.3 (1825) GGTAAAAACTGAACTATGTAAATGACTCTACTAAAAGTTTAGGGA
 FCGR1B_NM_001004340.3 (1549) GGTAAAAACTGAACTATGTAAATGACTCTACTAAAAGTTTAGGGA
 FCGR1B_XM_017000662.1 (1570) GGTAAAAACTGAACTATGTAAATGACTCTACTAAAAGTTTAGGGA
 FCGR1B_XM_017000661.1 (1828) GGTAAAAACTGAACTATGTAAATGACTCTACTAAAAGTTTAGGGA
 FCGR1B_XR_001737041.1 (2176) GGTAAAAACTGAACTATGTAAATGACTCTACTAAAAGTTTAGGGA
 FCGR1B_XR_001737040.1 (2113) GGTAAAAACTGAACTATGTAAATGACTCTACTAAAAGTTTAGGGA
 FCGR1B_NR_045213.1 (2110) GGTAAAAACTGAACTATGTAAATGACTCTACTAAAAGTTTAGGGA
 FCGR1CP_NR_027484.2 (1353) ---------------------------------------------
 Consensus (2206) GGTAAAAACTGAACTATGTAAATGACTCTACTAAAAGTTTAGGGA
                                                    Section 51
                     (2251) 2251     2260      2270      2280       2295
 FCGR1A_NM_000566.3 (2152) AAAAAAACAGGAGGAGTATGACACAAAAAAAAAAAAAAAAAAAAA
 FCGR1A_XM_005244957.3 (1337) ---------------------------------------------
 FCGR1A_XM_005244958.4 (1075) ---------------------------------------------
 FCGR1B_NM_001244910.1 (1582) ---------------------------------------------
 FCGR1B_NM_001017986.3 (1870) AAAAAAACAGGAGGAGTATGACACACACAGCAAAAAAAAAAAAAA
 FCGR1B_NM_001004340.3 (1594) AAAAAAACAGGAGGAGTATGACACACACAGCAAAAAAAAAAAAAA
 FCGR1B_XM_017000662.1 (1615) AAAAAAACAGGAGGAGTATGACACACACAGCAA------------
 FCGR1B_XM_017000661.1 (1873) AAAAAAACAGGAGGAGTATGACACACACAGCAA------------
 FCGR1B_XR_001737041.1 (2221) AAAAAAACAGGAGGAGTATGACACACACAGCAA------------
 FCGR1B_XR_001737040.1 (2158) AAAAAAACAGGAGGAGTATGACACACACAGCAA------------
 FCGR1B_NR_045213.1 (2155) AAAAAAACAGGAGGAGTATGACACACACAGCAAAAAAAAAAAAAA
 FCGR1CP_NR_027484.2 (1353) ---------------------------------------------
 Consensus (2251) AAAAAAACAGGAGGAGTATGACACACACAGCAA

                                                    Section 52
                     (2296) 2296           2310      2320      2330     2340
 FCGR1A_NM_000566.3 (2197) AAAAAAAAAAAAAAAAAAAAAAAAAAAAAAAAAAAAAAAAAAAAA
 FCGR1A_XM_005244957.3 (1337) ---------------------------------------------
 FCGR1A_XM_005244958.4 (1075) ---------------------------------------------
 FCGR1B_NM_001244910.1 (1582) ---------------------------------------------
 FCGR1B_NM_001017986.3 (1915) AAA------------------------------------------
 FCGR1B_NM_001004340.3 (1639) AAA------------------------------------------
 FCGR1B_XM_017000662.1 (1648) ---------------------------------------------
 FCGR1B_XM_017000661.1 (1906) ---------------------------------------------
 FCGR1B_XR_001737041.1 (2254) ---------------------------------------------
 FCGR1B_XR_001737040.1 (2191) ---------------------------------------------
 FCGR1B_NR_045213.1 (2200) AAA------------------------------------------
 FCGR1CP_NR_027484.2 (1353) ---------------------------------------------
 Consensus (2296)
                                                    Section 53
                     (2341) 2341     2350            2367
 FCGR1A_NM_000566.3 (2242) AAAAAAAAAAAAAAAAAAAAAAAAAAA
 FCGR1A_XM_005244957.3 (1337) ---------------------------
 FCGR1A_XM_005244958.4 (1075) ---------------------------
 FCGR1B_NM_001244910.1 (1582) ---------------------------
 FCGR1B_NM_001017986.3 (1918) ---------------------------
 FCGR1B_NM_001004340.3 (1642) ---------------------------
 FCGR1B_XM_017000662.1 (1648) ---------------------------
 FCGR1B_XM_017000661.1 (1906) ---------------------------
 FCGR1B_XR_001737041.1 (2254) ---------------------------
 FCGR1B_XR_001737040.1 (2191) ---------------------------
 FCGR1B_NR_045213.1 (2203) ---------------------------
 FCGR1CP_NR_027484.2 (1353) ---------------------------
 Consensus (2341)
```

Figure S6. Sequence alignment of transcripts of *FCGR1A*, *FCGR1B*, and *FCGR1CP*.

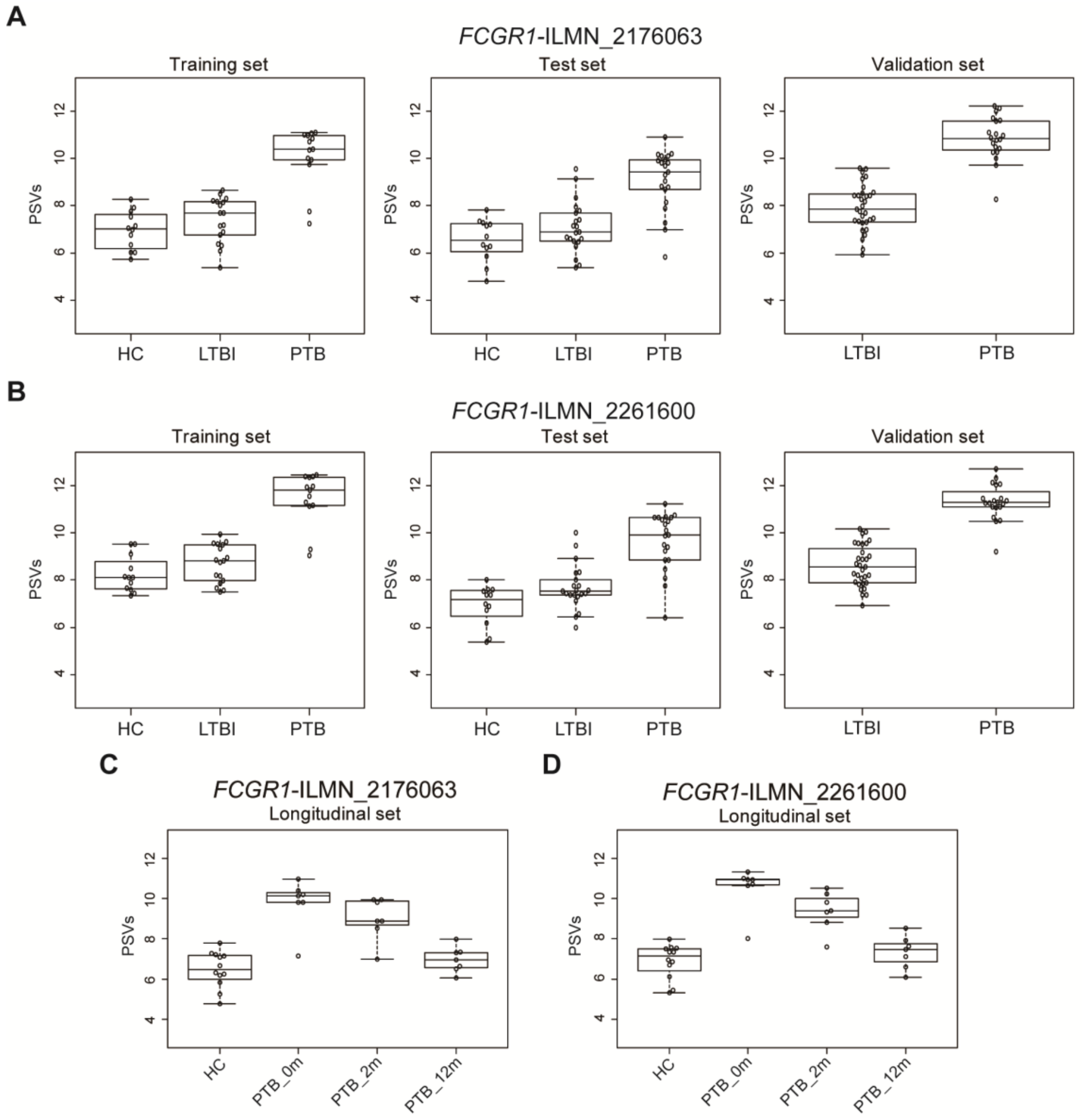

Figure S7: The box plots of the PSVs of *FCGR1* in whole blood.

(A & B) The PSVs from ILMN_2176063 and ILMN_2261600 respectively in whole blood of HC donors, LTBI donors and PTB patients from different cohorts.

(C & D) The PSVs from ILMN_2176063 and ILMN_2261600 respectively in whole blood of HC donors or PTB patients at different stages of chemotherapy. PTB_0m, PTB patients without chemotherapy; PTB_2m, PTB patients at 2 months post drug initiation; PTB_12m, PTB patients at 12 months post drug initiation.

```
                                                                                     Section 1
                                          (1) 1          10         20                 38
  promoter of FCGR1A other transcripts    (1) ----------------------------------TATA
  promoter of FCGR1A XM_005244958.4       (1) -----------------ATTTTATCCTTCCAGGCTATA
  promoter of FCGR1B all transcripts      (1) CTTATTAGAAAGAAAGTATTTTATCCTTCCAGGCTATA
  promoter of FCGR1CP                     (1) CTTATTAGAAAGAAAGTATTTTATCCTTCCAGGCTATA
  Consensus                               (1) CTTATTAGAAAGAAAGTATTTTATCCTTCCAGGCTATA
                                                                                     Section 2
                                         (39) 39         50         60                 76
  promoter of FCGR1A other transcripts    (5) GATCTTACATTCCTCTTACACAACAATCCTAATTCCCT
  promoter of FCGR1A XM_005244958.4      (22) GATCTTACATTCCTCTTACACAACAATCCTAATTCCCT
  promoter of FCGR1B all transcripts     (39) GATCTTACATTCCTCTTACACAACAATCCTAATTCCCT
  promoter of FCGR1CP                    (39) GATCTTACATTCCTCTTACACAACAATCCTAATTCCCT
  Consensus                              (39) GATCTTACATTCCTCTTACACAACAATCCTAATTCCCT
                                                                                     Section 3
                                         (77) 77         90         100               114
  promoter of FCGR1A other transcripts   (43) CATGTTATCCTGATCAATGAAATGATTTCACTTATTTT
  promoter of FCGR1A XM_005244958.4      (60) CATGTTATCCTGATCAATGAAATGATTTCACTTATTTT
  promoter of FCGR1B all transcripts     (77) CATGTTATCCTGATCAATGAAATGATTTCACTTATTTT
  promoter of FCGR1CP                    (77) CATGTTATCCTGATCAATGAAATGATTTCACTTATTTT
  Consensus                              (77) CATGTTATCCTGATCAATGAAATGATTTCACTTATTTT
                                                                                     Section 4
                                        (115) 115  120        130        140          152
  promoter of FCGR1A other transcripts   (81) TCAAAGAAGTAGCTATTTTACTACAAAGCAGGAATCGC
  promoter of FCGR1A XM_005244958.4      (98) TCAAAGAAGTAGCTATTTTACTACAAAGCAGGAATCGC
  promoter of FCGR1B all transcripts    (115) TCAAAGAAGTAGCTATTTTACTACAAAGCAGGAATCAC
  promoter of FCGR1CP                   (115) TCAAAGAAGTAGCTATTTTACTACAAAGCAGGAATCGC
  Consensus                             (115) TCAAAGAAGTAGCTATTTTACTACAAAGCAGGAATCGC
                                                                                     Section 5
                                        (153) 153    160        170        180        190
  promoter of FCGR1A other transcripts  (119) AAGGCATGGGGTGGGCACTGTTATACTCCATCAGTGAG
  promoter of FCGR1A XM_005244958.4     (136) AAGGCATGGGGTGGGCACTGTTATACTCCATCAGTGAG
  promoter of FCGR1B all transcripts    (153) AAGGCATGGGGTGGGCACTGTTATACTCCATCAGTGAG
  promoter of FCGR1CP                   (153) AAGGCATGGGGTGGGCACTGTTATACTCCATCAGTGAG
  Consensus                             (153) AAGGCATGGGGTGGGCACTGTTATACTCCATCAGTGAG
                                                                                     Section 6
                                        (191) 191        200        210                228
  promoter of FCGR1A other transcripts  (157) TACGTAAAGGAAATCAAACTGTCTGATTTTGAATCCTG
  promoter of FCGR1A XM_005244958.4     (174) TACGTAAAGGAAATCAAACTGTCTGATTTTGAATCCTG
  promoter of FCGR1B all transcripts    (191) TATGTAAAGGAAATCAAACTGTCTGATTTTGAATCCTG
  promoter of FCGR1CP                   (191) TATGTAAAGGAAATCAAACTGTCTGATTTTGAATCCTG
  Consensus                             (191) TATGTAAAGGAAATCAAACTGTCTGATTTTGAATCCTG
                                                                                     Section 7
                                        (229) 229          240        250              266
  promoter of FCGR1A other transcripts  (195) CTTCTATTTTTTACAACCTTGGACAAATTACTGATTTC
  promoter of FCGR1A XM_005244958.4     (212) CTTCTATTTTTTACAACCTTGGACAAATTACTGATTTC
  promoter of FCGR1B all transcripts    (229) CTTCTATTTTTCACAACCTTGGACAAATTACTGATTTC
  promoter of FCGR1CP                   (229) CTTCTATTTTTTACAACCTTGGACAAATTACTGATTTC
  Consensus                             (229) CTTCTATTTTTTACAACCTTGGACAAATTACTGATTTC
```

Section 8		
	(267)	267 280 290 304
promoter of FCGR1A other transcripts	(233)	CTCTTTCACTAGGTTTATCATTTGTAAAATGGAGATAC
promoter of FCGR1A XM_005244958.4	(250)	CTCTTTCACTAGGTTTATCATTTGTAAAATGGAGATAC
promoter of FCGR1B all transcripts	(267)	CTCTTTCACTAGGTTTATCATTTGTAAAATGGAGATAC
promoter of FCGR1CP	(267)	CTCTTTCACTAGGTTTATCATTTGTAAAATGGAGATAC
Consensus	(267)	CTCTTTCACTAGGTTTATCATTTGTAAAATGGAGATAC

Section 9		
	(305)	305 310 320 330 342
promoter of FCGR1A other transcripts	(271)	TAGTGTCTACTCATGGATTATTGTGAGGCTTAAATAAG
promoter of FCGR1A XM_005244958.4	(288)	TAGTGTCTACTCATGGATTATTGTGAGGCTTAAATAAG
promoter of FCGR1B all transcripts	(305)	TAGTGTCTACTCATGGATTATTGTGAGGCTTAAATAAG
promoter of FCGR1CP	(305)	TAGTGTCTACTCATGGATTATTGTGAGGCTTAAATAAG
Consensus	(305)	TAGTGTCTACTCATGGATTATTGTGAGGCTTAAATAAG

Section 10		
	(343)	343 350 360 370 380
promoter of FCGR1A other transcripts	(309)	AAAACATGTATCAAGCATATTTGTCTGGCATGTAATAA
promoter of FCGR1A XM_005244958.4	(326)	AAAACATGTATCAAGCATATTTGTCTGGCATGTAATAA
promoter of FCGR1B all transcripts	(343)	AAAACATGTATCAAGCATATTTGTCTGGCTTGTAATAA
promoter of FCGR1CP	(343)	AAAACATGTATCAAGCATATTTGTCTGGCATGTAATAA
Consensus	(343)	AAAACATGTATCAAGCATATTTGTCTGGCATGTAATAA

Section 11		
	(381)	381 390 400 418
promoter of FCGR1A other transcripts	(347)	GCACTCTAAAATTTATGCAGTGATGAGGATAATGATCA
promoter of FCGR1A XM_005244958.4	(364)	GCACTCTAAAATTTATGCAGTGATGAGGATAATGATCA
promoter of FCGR1B all transcripts	(381)	GCACTCTAAAATTTATGCAGTGATGAGGATAATGATCA
promoter of FCGR1CP	(381)	GCACTCTAAAATTTATGCAGCGATGAGGATAATGATCC
Consensus	(381)	GCACTCTAAAATTTATGCAGTGATGAGGATAATGATCA

Section 12		
	(419)	419 430 440 456
promoter of FCGR1A other transcripts	(385)	TAGACAATCTGATAGCATCTTATCCAAAGGAAAAAATG
promoter of FCGR1A XM_005244958.4	(402)	TAGACAATCTGATAGCATCTTATCCAAAGGAAAAAATG
promoter of FCGR1B all transcripts	(419)	TAGACAATCTGATAGCATCTTATCCAAAGGAAAAAATG
promoter of FCGR1CP	(419)	TAGACAATCTGATAGCATCTTATCCAAAGGAAAAAATG
Consensus	(419)	TAGACAATCTGATAGCATCTTATCCAAAGGAAAAAATG

Section 13		
	(457)	457 470 480 494
promoter of FCGR1A other transcripts	(423)	CGCATTTCCTTTGAGTAAGCACTACTGATTCTAGGAAT
promoter of FCGR1A XM_005244958.4	(440)	CGCATTTCCTTTGAGTAAGCACTACTGATTCTAGGAAT
promoter of FCGR1B all transcripts	(457)	CGCATTTCCTTTGAGTAAGCACTACTGATTCTAGGAAT
promoter of FCGR1CP	(457)	CGCATTTCCTTTGAGTAAGCACTACTGATTCTAGGAAT
Consensus	(457)	CGCATTTCCTTTGAGTAAGCACTACTGATTCTAGGAAT

Section 14		
	(495)	495 500 510 520 532
promoter of FCGR1A other transcripts	(461)	GTGTCCTAAGGAAAAAATAAGTCAAATGTTTAAAGGTA
promoter of FCGR1A XM_005244958.4	(478)	GTGTCCTAAGGAAAAAATAAGTCAAATGTTTAAAGGTA
promoter of FCGR1B all transcripts	(495)	GTGTCCTAAGGAAAAAATAAGTCAAATGTTTAAAGGTA
promoter of FCGR1CP	(495)	GTGTCCTAAGGAAAAAATAAGTCAAATGTTTAAAGGTA
Consensus	(495)	GTGTCCTAAGGAAAAAATAAGTCAAATGTTTAAAGGTA

Section 15

		533 540 550 560 570
	(533)	
promoter of FCGR1A other transcripts	(499)	GTCACAAGACTATTTTGGTAGTAAGCAGTCTTTTTTAC
promoter of FCGR1A XM_005244958.4	(516)	GTCACAAGACTATTTTGGTAGTAAGCAGTCTTTTTTAC
promoter of FCGR1B all transcripts	(533)	GTCACAAGACTATTTTGGTAGTAAGCAGTCTTTTTTAC
promoter of FCGR1CP	(533)	GTCACAAGACTATTTTGGTAGTAAGCAGTCTTTTTTAC
Consensus	(533)	GTCACAAGACTATTTTGGTAGTAAGCAGTCTTTTTTAC

Section 16

		571 580 590 608
	(571)	
promoter of FCGR1A other transcripts	(537)	TGGTCACACAGTAAAAAATTAGAAATAACCTACATGTT
promoter of FCGR1A XM_005244958.4	(554)	TGGTCACACAGTAAAAAATTAGAAATAACCTACATGTT
promoter of FCGR1B all transcripts	(571)	TGGTCACACAGTAAAAAATTAGAAATAACCTACATGTT
promoter of FCGR1CP	(571)	TGGTCACACAGTAAAAAATTAGAAATAACCTACATGTT
Consensus	(571)	TGGTCACACAGTAAAAAATTAGAAATAACCTACATGTT

Section 17

		609 620 630 646
	(609)	
promoter of FCGR1A other transcripts	(575)	CATGAAGAGAAGTTGGTGAAATAAATTATGGTACATGC
promoter of FCGR1A XM_005244958.4	(592)	CATGAAGAGAAGTTGGTGAAATAAATTATGGTACATGC
promoter of FCGR1B all transcripts	(609)	CATGAAGAGAAGTTGGTGAAATAAATTATGGTACATGC
promoter of FCGR1CP	(609)	CATGAAGAGAAGTTGGTGAAATAAATTATGGTACATGC
Consensus	(609)	CATGAAGAGAAGTTGGTGAAATAAATTATGGTACATGC

Section 18

		647 660 670 684
	(647)	
promoter of FCGR1A other transcripts	(613)	ATACAGTAGAAAACTTTGGAGACAAAATAGTAAAATAG
promoter of FCGR1A XM_005244958.4	(630)	ATACAGTAGAAAACTTTGGAGACAAAATAGTAAAATAG
promoter of FCGR1B all transcripts	(647)	ATACAGTAGAAAACTTTGGAGACAAAATAGTAAAATAG
promoter of FCGR1CP	(647)	ATACAGTAGAAAACTTTGGAGACAAAATAGTAAAATAG
Consensus	(647)	ATACAGTAGAAAACTTTGGAGACAAAATAGTAAAATAG

Section 19

		685 690 700 710 722
	(685)	
promoter of FCGR1A other transcripts	(651)	TTACAGATATAGGCTTTGAATATAAATTTTAACAAGCT
promoter of FCGR1A XM_005244958.4	(668)	TTACAGATATAGGCTTTGAATATAAATTTTAACAAGCT
promoter of FCGR1B all transcripts	(685)	TTACAGATATAGGCTTTGAATATAAATTTTAACAAGCT
promoter of FCGR1CP	(685)	TTACAGATATAGGCTTTGAATATAAATTTTAACAAGCT
Consensus	(685)	TTACAGATATAGGCTTTGAATATAAATTTTAACAAGCT

Section 20

		723 730 740 750 760
	(723)	
promoter of FCGR1A other transcripts	(689)	GAGGCTTCTCTCGTCTCTTCAGAACACCCTGAGTTCTT
promoter of FCGR1A XM_005244958.4	(706)	GAGGCTTCTCTCGTCTCTTCAGAACACCCTGAGTTCTT
promoter of FCGR1B all transcripts	(723)	GAGGCTTCTCTCATCTCTTCAGAACACCCTGAGTTCTT
promoter of FCGR1CP	(723)	GAGGCTTCTCTCGTCTCTTCAGAACACCCTGAGTTCTT
Consensus	(723)	GAGGCTTCTCTCGTCTCTTCAGAACACCCTGAGTTCTT

Section 21

		761 770 780 798
	(761)	
promoter of FCGR1A other transcripts	(727)	CCCGGCCTTTCTTCCTTGCTTTTCATTTGTGTCAGTCA
promoter of FCGR1A XM_005244958.4	(744)	CCCGGCCTTTCTTCCTTGCTTTTCATTTGTGTCAGTCA
promoter of FCGR1B all transcripts	(761)	CCCGGCCTTTCTTCCTTGCTTTTCATTTGTGTCAGTCA
promoter of FCGR1CP	(761)	CCCGGCCTTTCTTCCTTGCTTTTCATTTGTGTCAGTCA
Consensus	(761)	CCCGGCCTTTCTTCCTTGCTTTTCATTTGTGTCAGTCA

```
                                                     Section 22
                                        (799) 799        810       820             836
promoter of FCGR1A other transcripts    (765) GCTCTTGCTCGTATAGCAATCCTCAAATCTCAGTAGCA
promoter of FCGR1A XM_005244958.4       (782) GCTCTTGCTCGTATAGCAATCCTCAAATCTCAGTAGCA
promoter of FCGR1B all transcripts      (799) GCTCTTGCTTGTATAGCAATCTTCAAATCTCAGTAGCA
promoter of FCGR1CP                     (799) GCTCTTGCTTGTACAGCAATCTTCAAATCTCAGTAGCA
Consensus                               (799) GCTCTTGCTTGTATAGCAATCTTCAAATCTCAGTAGCA
                                                     Section 23
                                        (837) 837          850       860           874
promoter of FCGR1A other transcripts    (803) TATAAAGGTAAGCATTTAGTTCTCACTTACAAGTTTGC
promoter of FCGR1A XM_005244958.4       (820) TATAAAGGTAAGCATTTAGTTCTCACTTACAAGTTTGC
promoter of FCGR1B all transcripts      (837) TATAAAGGTAAGCATTTAGTTCTCACTTACAAGTTTGC
promoter of FCGR1CP                     (837) TATAAAGGTAAGCATTTAGTTCTCACTTACAAGTTTGC
Consensus                               (837) TATAAAGGTAAGCATTTAGTTCTCACTTACAAGTTTGC
                                                     Section 24
                                        (875) 875  880       890       900         912
promoter of FCGR1A other transcripts    (841) AGGGCAGGTGAGGCAGCTCTGCCTCAAGCCACAATGTC
promoter of FCGR1A XM_005244958.4       (858) AGGGCAGGTGAGGCAGCTCTGCCTCAAGCCACAATGTC
promoter of FCGR1B all transcripts      (875) AGGGCAGGTGAGGCAGGTCTGCCTCAAGCCACAATGTC
promoter of FCGR1CP                     (875) AGGGCAGGTGAGGCAGCTCTGCCTCAAGCCACAATGTC
Consensus                               (875) AGGGCAGGTGAGGCAGCTCTGCCTCAAGCCACAATGTC
                                                     Section 25
                                        (913) 913    920       930       940       950
promoter of FCGR1A other transcripts    (879) TAGGACAGCTCTGTTTCGCACTGTGGCTGACTAGCTAA
promoter of FCGR1A XM_005244958.4       (896) TAGGACAGCTCTGTTTCGCACTGTGGCTGACTAGCTAA
promoter of FCGR1B all transcripts      (913) TAGGACAGCTCTGTTTCACACGGTGGCTGACTAGCTAA
promoter of FCGR1CP                     (913) TAGGACAGCTCTGTTTCGCACGGTGGCTGACTAGCTAA
Consensus                               (913) TAGGACAGCTCTGTTTCGCACTGTGGCTGACTAGCTAA
                                                     Section 26
                                        (951) 951     960       970                988
promoter of FCGR1A other transcripts    (917) AAAATGTTCTTGTCTCAGCAATCACAGGAGAACAAGAG
promoter of FCGR1A XM_005244958.4       (934) AAAATGTTCTTGTCTCAGCAATCACAGGAGAACAAGAG
promoter of FCGR1B all transcripts      (951) AAAATGTTCTTGTCTCAGCAATCACAGGAGAACAAGAG
promoter of FCGR1CP                     (951) AAAATGTTCTTGTCTCAGCAATCACAGGAGAACAAGAG
Consensus                               (951) AAAATGTTCTTGTCTCAGCAATCACAGGAGAACAAGAG
                                                     Section 27
                                        (989) 989        1000      1010           1026
promoter of FCGR1A other transcripts    (955) GGTGAGAGATAATGTGTGAAGCTTCCTAAGTGCTAAGT
promoter of FCGR1A XM_005244958.4       (972) GGTGAGAGATAATGTGTGAAGCTTCCTAAGTGCTAAGT
promoter of FCGR1B all transcripts      (989) GGTGAGAGATAATGTGTGAAGCTTCCTAAGTGCTAAGT
promoter of FCGR1CP                     (989) GGTGAGAGATAATGTGTGAAGCTTCCTAAGTGCTAAGT
Consensus                               (989) GGTGAGAGATAATGTGTGAAGCTTCCTAAGTGCTAAGT
                                                     Section 28
                                       (1027) 1027         1040      1050          1064
promoter of FCGR1A other transcripts    (993) TTGAAATTAGCACCATGTCATTTCCATTCACATTTCGC
promoter of FCGR1A XM_005244958.4      (1010) TTGAAATTAGCACCATGTCATTTCCATTCACATTTCGC
promoter of FCGR1B all transcripts     (1027) TTGAAATTAGCACCATGTCATTTCCATTCACATTTCGC
promoter of FCGR1CP                    (1027) TTGAAATTAGCACCATGTCATTTCCATTCACATTTCGC
Consensus                              (1027) TTGAAATTAGCACCATGTCATTTCCATTCACATTTCGC
```

```
                                                                     Section 29
                                         (1065) 1065 1070          1080          1090           1102
promoter of FCGR1A other transcripts     (1031) TGGCCAATGAAAGTCTCATGATGAGCCACAGTCAAGAG
promoter of FCGR1A XM_005244958.4        (1048) TGGCCAATGAAAGTCTCATGATGAGCCACAGTCAAGAG
promoter of FCGR1B all transcripts       (1065) TGGCCAATGAAAGTCTCATGATGAGCCACAGTCAAGAG
promoter of FCGR1CP                      (1065) TGGCCAATGAAAGTCTCATGATGAGCCACAGTCAAGAG
Consensus                                (1065) TGGCCAATGAAAGTCTCATGATGAGCCACAGTCAAGAG
                                                                     Section 30
                                         (1103) 1103   1110          1120          1130     1140
promoter of FCGR1A other transcripts     (1069) TCAGAGACATACCCTCATGAGGCTGTGGAGAGAGTGTA
promoter of FCGR1A XM_005244958.4        (1086) TCAGAGACATACCCTCATGAGGCTGTGGAGAGAGTGTA
promoter of FCGR1B all transcripts       (1103) TCAGAGATGTACCCTCATGAGGCTGTGGAGAGAGTGTA
promoter of FCGR1CP                      (1103) TCAGAGACGTACCCTCATGAGGCTGTGGAGAGAGTGTA
Consensus                                (1103) TCAGAGACGTACCCTCATGAGGCTGTGGAGAGAGTGTA
                                                                     Section 31
                                         (1141) 1141     1150          1160                 1178
promoter of FCGR1A other transcripts     (1107) GATTCAGGGAGCGGTGAGAAACTGGGGCCAGTACACCA
promoter of FCGR1A XM_005244958.4        (1124) GATTCAGGGAGCGGTGAGAAACTGGGGCCAGTACACCA
promoter of FCGR1B all transcripts       (1141) GATTCAGGGAGCGGTGAGAAACTGGGGCCAGTACACCA
promoter of FCGR1CP                      (1141) GATTCAGGGAGCGGTGAGAAACTGGGGCCAGTACACCA
Consensus                                (1141) GATTCAGGGAGCGGTGAGAAACTGGGGCCAGTACACCA
                                                                     Section 32
                                         (1179) 1179         1190          1200             1216
promoter of FCGR1A other transcripts     (1145) ACTTACAGCATGATTTGTCATCATTTTTCATTCTCTGC
promoter of FCGR1A XM_005244958.4        (1162) ACTTACAGCATGATTTGTCATCATTTTTCATTCTCTGC
promoter of FCGR1B all transcripts       (1179) ATCTACAGCATGATTTGTCATCATTTTTCATTCTCTGC
promoter of FCGR1CP                      (1179) ATCTACAGCATGATTTGTCATCATTTTTCATTCTCTGC
Consensus                                (1179) ATTTACAGCATGATTTGTCATCATTTTTCATTCTCTGC
                                                                     Section 33
                                         (1217) 1217           1230          1240           1254
promoter of FCGR1A other transcripts     (1183) TAGTAAGATTGCCAAAGTCTCTCTGCACTCATTGAATT
promoter of FCGR1A XM_005244958.4        (1200) TAGTAAGATTGCCAAAGTCTCTCTGCACTCATTGAATT
promoter of FCGR1B all transcripts       (1217) TAGTAAGATTGCCAAAGTCTCTCTGCACTCATTGAATT
promoter of FCGR1CP                      (1217) TAGTAAGATTGCCAAAGTCTCTCTGCACTCATTGAATT
Consensus                                (1217) TAGTAAGATTGCCAAAGTCTCTCTGCACTCATTGAATT
                                                                     Section 34
                                         (1255) 1255 1260          1270          1280       1292
promoter of FCGR1A other transcripts     (1221) CATTTAGCTCTCTTTAGCTCTCTTTTTTTAGCTCTCAT
promoter of FCGR1A XM_005244958.4        (1238) CATTTAGCTCTCTTTAGCTCTCTTTTTTTAGCTCTCAT
promoter of FCGR1B all transcripts       (1255) CATCC---------------------------------
promoter of FCGR1CP                      (1255) CATCC---------------------------------
Consensus                                (1255) CATTTAGCTCTCTTTAGCTCTCTTTTTTTAGCTCTCAT
                                                                     Section 35
                                         (1293) 1293   1300          1310          1320     1330
promoter of FCGR1A other transcripts     (1259) TTAGTTTCCATTTAGCTCTCTTTCTCTCTTACCACAAT
promoter of FCGR1A XM_005244958.4        (1276) TTAGTTTCCATTTAGCTCTCTTTCTCTCTTACCACAAT
promoter of FCGR1B all transcripts       (1260) -TAGTTTCCATTTAGCTCTCTTTCTCTCTTACCACAAT
promoter of FCGR1CP                      (1260) -TAGTTTCCATTTAGCTCTCTTTCTCTCTTACCACAAT
Consensus                                (1293) TTAGTTTCCATTTAGCTCTCTTTCTCTCTTACCACAAT
```

```
                                                                                              Section 36
                                        (1331) 1331      ,1340      ,1350                    1368
promoter of FCGR1A other transcripts (1297) ACCTAAAAATCTTTTTATGGGGTTACAACTCTGTCTCA
  promoter of FCGR1A XM_005244958.4 (1314) ACCTAAAAATCTTTTTATGGGGTTACAACTCTGTCTCA
    promoter of FCGR1B all transcripts (1297) ACCTAAAAATCTTTTTATGGGGTTACAACTCTGTCTCA
              promoter of FCGR1CP (1297) ACCTAAAAATCTTTTTATGGGGTTACAACTCTGTCTCA
                        Consensus (1331) ACCTAAAAATCTTTTTATGGGGTTACAACTCTGTCTCA
                                                                                              Section 37
                                        (1369) 1369      ,1380      ,1390                    1406
promoter of FCGR1A other transcripts (1335) GGAGCCACCTCAGTTTGGTGGCACAGAGCTCCCCACTT
  promoter of FCGR1A XM_005244958.4 (1352) GGAGCCACCTCAGTTTGGTGGCACAGAGCTCCCCACTT
    promoter of FCGR1B all transcripts (1335) GGAGCCACCTCAGTTTGGTGGCACAGAGCTCCCCACTT
              promoter of FCGR1CP (1335) GGAGCCACCTCAGTTTGGTGGCACAGAGCTCCCCACTT
                        Consensus (1369) GGAGCCACCTCAGTTTGGTGGCACAGAGCTCCCCACTT
                                                                                              Section 38
                                        (1407) 1407          ,1420      ,1430                1444
promoter of FCGR1A other transcripts (1373) GTACTCTGAAACTCTTTTCTTGAAGATATTGCTATCCA
  promoter of FCGR1A XM_005244958.4 (1390) GTACTCTGAAACTCTTTTCTTGAAGATATTGCTATCCA
    promoter of FCGR1B all transcripts (1373) GTACTCTGAAACTCTTTTCTTGAAGATATTGCTATCCA
              promoter of FCGR1CP (1373) GTACTCTGAAACTCTTTTCTTGAAGATATTGTTATCCA
                        Consensus (1407) GTACTCTGAAACTCTTTTCTTGAAGATATTGCTATCCA
                                                                                              Section 39
                                        (1445) 1445 ,1450      ,1460      ,1470             1482
promoter of FCGR1A other transcripts (1411) TGTGCATGAGTTATTTTTCTTCTCCATAGCATCCACAT
  promoter of FCGR1A XM_005244958.4 (1428) TGTGCATGAGTTATTTTTCTTCTCCATAGCATCCACAT
    promoter of FCGR1B all transcripts (1411) TGTGCATGAGTTATTTTTCTTCTCCATAGCATCCACAT
              promoter of FCGR1CP (1411) TGTGCATGAGTTATTTTTCTTCTCCATAGCATCCACAT
                        Consensus (1445) TGTGCATGAGTTATTTTTCTTCTCCATAGCATCCACAT
                                                                                              Section 40
                                        (1483) 1483   ,1490      ,1500      ,1510           1520
promoter of FCGR1A other transcripts (1449) TAAACAATTAATTACCTTGGATTTAGCCAAAATAAACA
  promoter of FCGR1A XM_005244958.4 (1466) TAAACAATTAATTACCTTGGATTTAGCCAAAATAAACA
    promoter of FCGR1B all transcripts (1449) TAAACAATTAATTACCTTGGATTTAGCCAAAATAAACA
              promoter of FCGR1CP (1449) TAAACAATTAATTACCTTGGATTTAGCCAAAATAAACA
                        Consensus (1483) TAAACAATTAATTACCTTGGATTTAGCCAAAATAAACA
                                                                                              Section 41
                                        (1521) 1521      ,1530      ,1540                    1558
promoter of FCGR1A other transcripts (1487) CTCCAAATTCATGATGTCTTTCAGGAAGAGAGTTTGTT
  promoter of FCGR1A XM_005244958.4 (1504) CTCCAAATTCATGATGTCTTTCAGGAAGAGAGTTTGTT
    promoter of FCGR1B all transcripts (1487) CTCCAAATTCATGATGTCTTTCAGGAAGAGAGTTTGTT
              promoter of FCGR1CP (1487) CTCCAAATTCATGATGTCTTTCAGGAAGAGAGTTTGTT
                        Consensus (1521) CTCCAAATTCATGATGTCTTTCAGGAAGAGAGTTTGTT
                                                                                              Section 42
                                        (1559) 1559      ,1570      ,1580                    1596
promoter of FCGR1A other transcripts (1525) TTCTTTTTTAACCAAAATGGGAGATTGATCCTGTCTGT
  promoter of FCGR1A XM_005244958.4 (1542) TTCTTTTTTAACCAAAATGGGAGATTGATCCTGTCTGT
    promoter of FCGR1B all transcripts (1525) TTCTTTTTTAACCAAAATGGGAGATTGATCCTGTCTGT
              promoter of FCGR1CP (1525) TTCTTTTTTAACCAAAATGGGAGATTGATCCTGTCTGT
                        Consensus (1559) TTCTTTTTTAACCAAAATGGGAGATTGATCCTGTCTGT
```

```
                                                                       Section 43
                                     (1597) 1597        1610      1620          1634
promoter of FCGR1A other transcripts (1563) GTCCCCTAGAGGAGCAGAGGCTGGTTTGTATTAGGGCA
promoter of FCGR1A XM_005244958.4    (1580) GTCCCCTAGAGGAGCAGAGGCTGGTTTGTATTAGGGCA
promoter of FCGR1B all transcripts   (1563) GTCCCCTAGAGGAGCAGAGGCTGGTTTGTATTAGGGCA
promoter of FCGR1CP                  (1563) GTCCCCTAGAGGAGCAGAGGCTGGTTTGTATTAGGGCA
Consensus                            (1597) GTCCCCTAGAGGAGCAGAGGCTGGTTTGTATTAGGGCA
                                                                       Section 44
                                     (1635) 1635 1640      1650      1660       1672
promoter of FCGR1A other transcripts (1601) GCTCTTGTAGATGAGTAACTTTTCCCATGGCCTCATAG
promoter of FCGR1A XM_005244958.4    (1618) GCTCTTGTAGATGAGTAACTTTTCCCATGGCCTCATAG
promoter of FCGR1B all transcripts   (1601) GCTCTTGTAGATGAGTAACTTTTCCCATGGCCTCATAG
promoter of FCGR1CP                  (1601) GCTCTTGTAGATGAGTAACTTTTCCCATGGCCTCATAG
Consensus                            (1635) GCTCTTGTAGATGAGTAACTTTTCCCATGGCCTCATAG
                                                                       Section 45
                                     (1673) 1673   1680      1690      1700     1710
promoter of FCGR1A other transcripts (1639) AGGCTGATATAGAAACTTCTGGATTCAAATGATTGTTT
promoter of FCGR1A XM_005244958.4    (1656) AGGCTGATATAGAAACTTCTGGATTCAAATGATTGTTT
promoter of FCGR1B all transcripts   (1639) AGGCTGATATAGAAACTTCTGGATTCAAATGATTGTTT
promoter of FCGR1CP                  (1639) AGGCTGATATAGATACTTCTGGATTCAAATGATTGTTT
Consensus                            (1673) AGGCTGATATAGAAACTTCTGGATTCAAATGATTGTTT
                                                                       Section 46
                                     (1711) 1711     1720      1730             1748
promoter of FCGR1A other transcripts (1677) GGAGGCATTAGCCAGGCATTGAACCAATTATAAAGAGT
promoter of FCGR1A XM_005244958.4    (1694) GGAGGCATTAGCCAGGCATTGAACCAATTATAAAGAGT
promoter of FCGR1B all transcripts   (1677) GGAGGCATTAGCCAGGCATTGAACCAATTATAAAGAGT
promoter of FCGR1CP                  (1677) GGAGGCATTAGCCAGGCATTGAACCAATTATAAAGAGT
Consensus                            (1711) GGAGGCATTAGCCAGGCATTGAACCAATTATAAAGAGT
                                                                       Section 47
                                     (1749) 1749       1760      1770           1786
promoter of FCGR1A other transcripts (1715) GAGGTTTTGCCATATTCTAACCGTAAGAAACAGAAACG
promoter of FCGR1A XM_005244958.4    (1732) GAGGTTTTGCCATATTCTAACCGTAAGAAACAGAAACG
promoter of FCGR1B all transcripts   (1715) GAGGTTTTGCCATATTCTAACCCTAAGAAACAGAAACG
promoter of FCGR1CP                  (1715) GAGGTTTTGCCATATTCTAACCGTAAGAAACAGAAACG
Consensus                            (1749) GAGGTTTTGCCATATTCTAACCGTAAGAAACAGAAACG
                                                                       Section 48
                                     (1787) 1787         1800      1810         1824
promoter of FCGR1A other transcripts (1753) GTAGAGGGTAAAAATAGAATAGAATGTCAATACAACTT
promoter of FCGR1A XM_005244958.4    (1770) GTAGAGGGTAAAAATAGAATAGAATGTCAATACAACTT
promoter of FCGR1B all transcripts   (1753) GTAGAGGGTAAAAATAGAATAGAATGTCAATACAACTT
promoter of FCGR1CP                  (1753) GTAGAGGGTAAAAATAGAATAGAATGTCAACACAACTT
Consensus                            (1787) GTAGAGGGTAAAAATAGAATAGAATGTCAATACAACTT
                                                                       Section 49
                                     (1825) 1825 1830      1840      1850       1862
promoter of FCGR1A other transcripts (1791) TAGCCTTTTCCCTGGATTCTGAGAGTTCATAACCTGAA
promoter of FCGR1A XM_005244958.4    (1808) TAGCCTTTTCCCTGGATTCTGAGAGTTCATAACCTGAA
promoter of FCGR1B all transcripts   (1791) TAGCCTTTTCCCTGGATTCTGAGAGTTCATAACCTGAA
promoter of FCGR1CP                  (1791) TAGCCTTTTCCCTGGATTCTGAGAGTTCATAACCTGAA
Consensus                            (1825) TAGCCTTTTCCCTGGATTCTGAGAGTTCATAACCTGAA
```

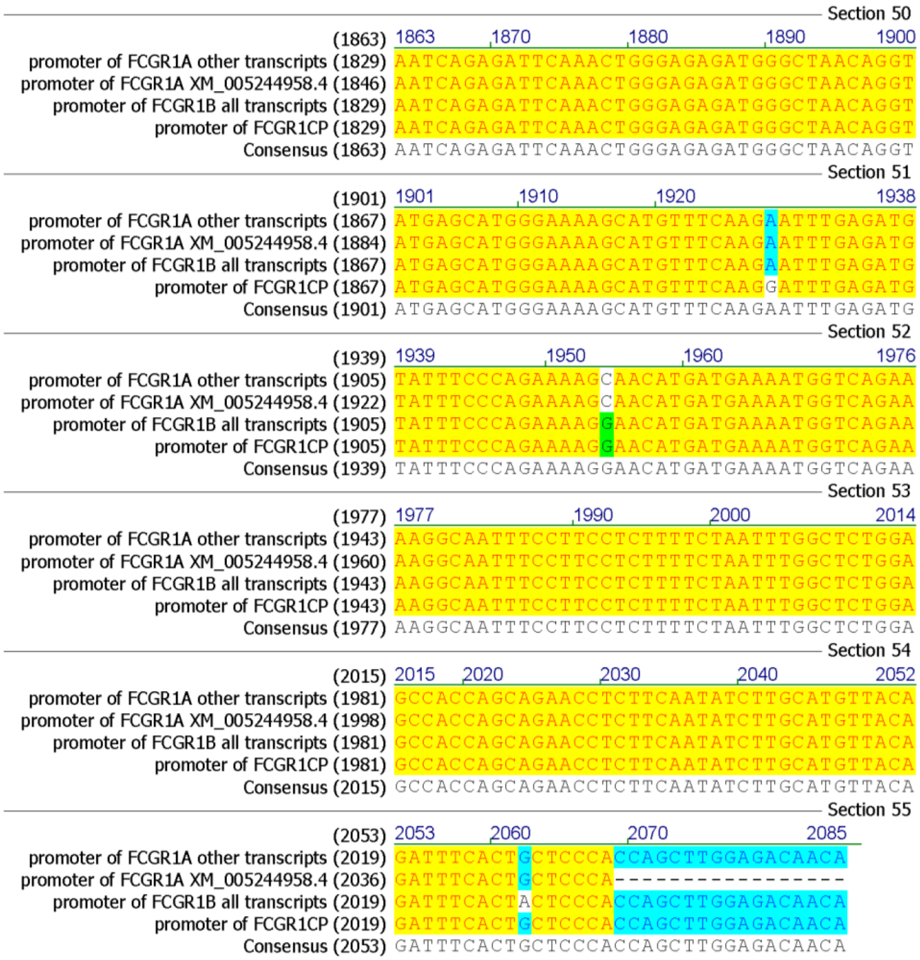

Figure S8: Sequence alignment of the promoters of *FCGR1A*, *FCGR1B*, and *FCGR1CP*.

Sequences from 2000 bp upstream to -50 downstream of transcription start site were used for sequence alignment.

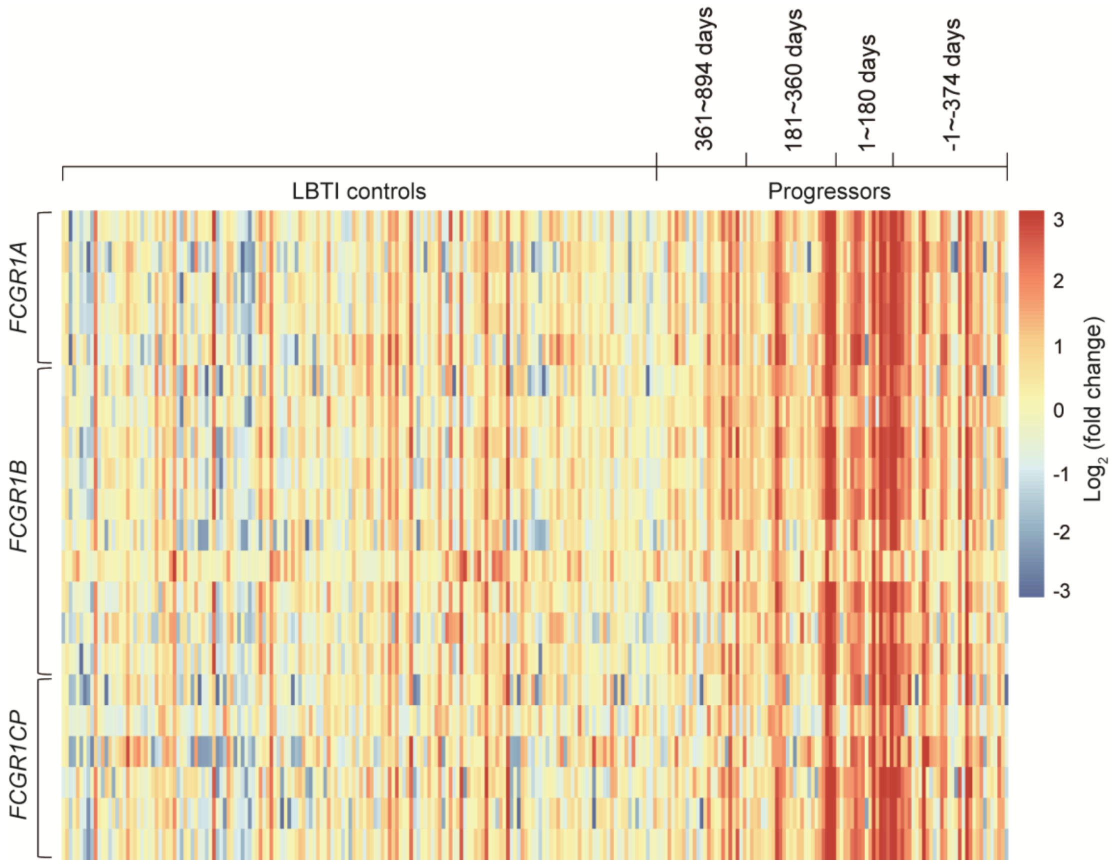

Figure S9: The relative expressions of *FCGR1A*, *FCGR1B*, and *FCGR1CP* in the whole blood of LTBI progressors compared to their expressions in the whole blood of LTBI controls.

This heat map is a visual representation of the data shown in Figures 5a-5c. Each row represents the relative expressions of a specific splice junction (1).

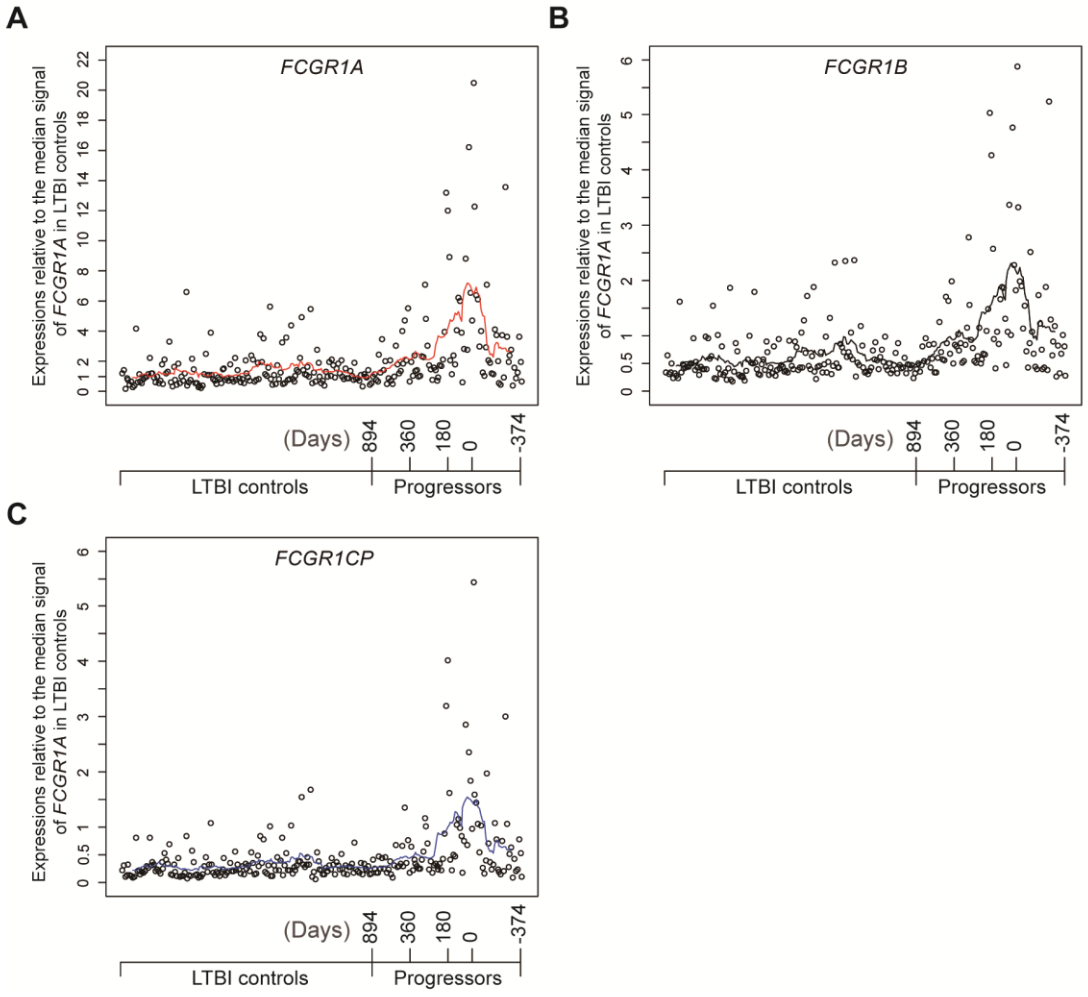

Figure S10: The PSVs of *FCGR1A* (A), *FCGR1B* (B) and *FCGR1CP* (C) relative to the median expression of *FCGR1A* in LTBI controls.

Data were from Figure S9, presented as described in Figure 5.

```
                                                                              Section 1
                      (1) 1           10             20             30             40         50
FCGR1A                (1) MWFLTTLLLWVPVDG-QVDTTKAVITLQPPWVSVFQEETVTLHCEVLHLP
FCGR1A isoform X1     (1) MWFLTTLLLWVPVDGQVVDTTKAVITLQPPWVSVFQEETVTLHCEVLHLP
FCGR1A isoform X2     (1) MWFLTTLLLWG---------------------------------------
FCGR1B isoform 1      (1) MWFLTTLLLWVPVDG-QVDTTKAVITLQPPWVSVFQEETVTLHCEVLHLP
FCGR1B isoform 2      (1) MWFLTTLLLWG---------------------------------------
FCGR1B isoform 3      (1) MWFLTTLLLWVPVDG-QVDTTKAVITLQPPWVSVFQEETVTLHCEVLHLP
FCGR1B isoform X1     (1) MWFLTTLLLWVPVDGQVVDTTKAVITLQPPWVSVFQEETITLHCEVLHLP
FCGR1B isoform X2     (1) MWFLTTLLLWVPVDG-----------------------------------
Consensus             (1) MWFLTTLLLWVPVDG  VDTTKAVITLQPPWVSVFQEETVTLHCEVLHLP
                                                                              Section 2
                     (51) 51          60             70             80             90        100
FCGR1A               (50) GSSSTQWFLNGTATQTSTPSYRITSASVNDSGEYRCQRGLSGRSDPIQLE
FCGR1A isoform X1    (51) GSSSTQWFLNGTATQTSTPSYRITSASVNDSGEYRCQRGLSGRSDPIQLE
FCGR1A isoform X2    (12) --------------------------------------------------
FCGR1B isoform 1     (50) GSSSTQWFLNGTATQTSTPSYRITSASVNDSGEYRCQRGLSGRSDPIQLE
FCGR1B isoform 2     (12) --------------------------------------------------
FCGR1B isoform 3     (50) GSSSTQWFLNGTATQTSTPSYRITSASVNDSGEYRCQRGLSGRSDPIQLE
FCGR1B isoform X1    (51) GSSSTQWFLNGTATQTSTPSYRITSASVNDSGEYRCQRGLSGRSDPIQLE
FCGR1B isoform X2    (16) --------------------------------------------------
Consensus            (51) GSSSTQWFLNGTATQTSTPSYRITSASVNDSGEYRCQRGLSGRSDPIQLE
                                                                              Section 3
                    (101) 101         110            120            130            140        150
FCGR1A              (100) IHRGWLLLQVSSRVFTEGEPLALRCHAWKDKLVYNVLYYRNGKAFKFFHW
FCGR1A isoform X1   (101) IHRGWLLLQVSSRVFTEGEPLALRCHAWKDKLVYNVLYYRNGKAFKFFHW
FCGR1A isoform X2    (12) ----WLLLQVSSRVFTEGEPLALRCHAWKDKLVYNVLYYRNGKAFKFFHW
FCGR1B isoform 1    (100) IHRGWLLLQVSSRVFMEGEPLALRCHAWKDKLVYNVLYYRNGKAFKFFHW
FCGR1B isoform 2     (12) ----WLLLQVSSRVFMEGEPLALRCHAWKDKLVYNVLYYRNGKAFKFFHW
FCGR1B isoform 3    (100) IHRGWLLLQVSSRVFMEGEPLALRCHAWKDKLVYNVLYYRNGKAFKFFHW
FCGR1B isoform X1   (101) IHRGWLLLQVSSRVFMEGEPLALRCHAWKDKLVYNVLYYRNGKAFKFFHW
FCGR1B isoform X2    (16) -QVGWLLLQVSSRVFMEGEPLALRCHAWKDKLVYNVLYYRNGKAFKFFHW
Consensus           (101) IHRGWLLLQVSSRVFMEGEPLALRCHAWKDKLVYNVLYYRNGKAFKFFHW
                                                                              Section 4
                    (151) 151         160            170            180            190        200
FCGR1A              (150) NSNLTILKTNISHNGTYHCSGMGKHRYTSAGISVTVKELFPAPVLNASVT
FCGR1A isoform X1   (151) NSNLTILKTNISHNGTYHCSGMGKHRYTSAGISVTVKELFPAPVLNASVT
FCGR1A isoform X2    (58) NSNLTILKTNISHNGTYHCSGMGKHRYTSAGISVTVKELFPAPVLNASVT
FCGR1B isoform 1    (150) NSNLTILKTNISHNGTYHCSGMGKHRYTSAGIS-----------------
FCGR1B isoform 2     (58) NSNLTILKTNISHNGTYHCSGMGKHRYTSAGIS-----------------
FCGR1B isoform 3    (150) NSNLTILKTNISHNGTYHCSGMGKHRYTSAGIS-----------------
FCGR1B isoform X1   (151) NSNLTILKTNISHNGTYHCSGMGKHRYTSAGIS-----------------
FCGR1B isoform X2    (65) NSNLTILKTNISHNGTYHCSGMGKHRYTSAGIS-----------------
Consensus           (151) NSNLTILKTNISHNGTYHCSGMGKHRYTSAGIS
```

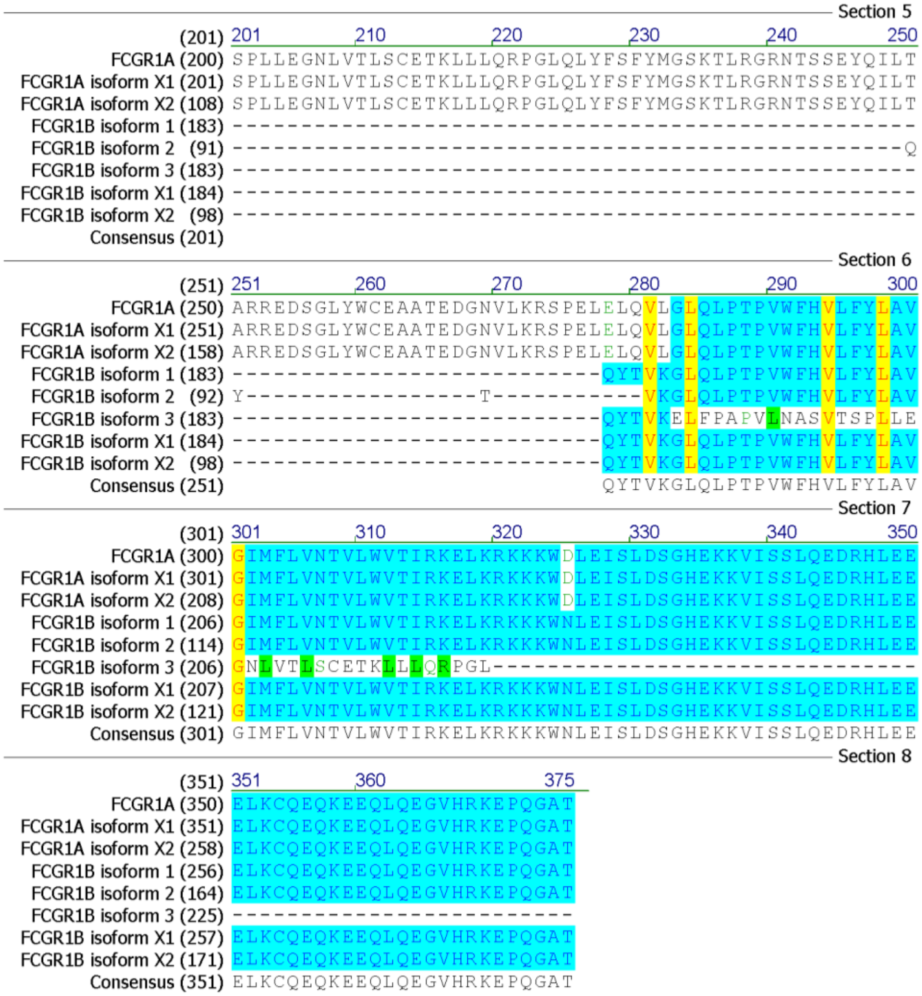

Figure S11: The sequence alignment of proteins of FCGR1A and FCGR1B.

Table S1. Genomic locations of *FCGR1* genes.

Organisms	Gene Symbol	Chromosome	Location	Genome Assembly
Human	*FCGR1A*	1	149782071-149792518	GRCh38.p13
Human	*FCGR1B*	1	121087345-121097161	GRCh38.p13

Human	*FCGR1CP*	1	143874743-143883733	GRCh38.p13
Mouse	*Fcgr1*	3	96282909-96293969, complement	GRCm38.p6

References

1. Zak DE, Penn-Nicholson A, Scriba TJ, Thompson E, Suliman S, Amon LM ,et al. A blood RNA signature for tuberculosis disease risk: a prospective cohort study. Lancet. (2016) 387:2312-2322. DOI:10.1016/S0140-6736(15)01316-1
